# Muscle synergy characteristics during hands-and-knees crawling in children aged 4–6 years

**DOI:** 10.1371/journal.pone.0358587

**Published:** 2026-10-08

**Authors:** Luyao Si, Yi Jia, Xuye Kang

**Affiliations:** 1 School of Sports and Physical Education, North University of China, Taiyuan, Shanxi, China; 2 School of Physical Education, Shanxi University, Taiyuan, China; SPRINT - Sport Physical Activity and Health Research & Innovation Center, PORTUGAL

## Abstract

Hands-and-knees crawling requires complex multi-joint coordination, managed by the central nervous system (CNS) via muscle synergies. While infant crawling is well-studied, neuromotor control in 4–6-year-olds remains under-investigated. This study examined the spatiotemporal consistency and developmental trajectories of muscle synergies during crawling in this age group. Kinematics and surface electromyography (sEMG) from 16 muscles were recorded from 49 healthy children performing self-paced crawling, segmenting ten cycles per participant. Muscle synergies were extracted using non-negative matrix factorization (NMF). Spatiotemporal characteristics were evaluated using cosine similarity (CS) and temporal phase dispersion (Circular SD). An average of 7.82±1.05 muscle synergies were extracted from the 16 recorded muscles, with synergy number increasing significantly with age (*p* < 0.05). Cluster analysis identified 12 group-level synergies. Proximal-dominated core shared synergies (M4–M7, M11) exhibited high prevalence (73.47%–87.76%) and stable spatial organization (mean CS = 0.68–0.86), without significant age-related differences. Conversely, specific synergies for distal adjustments (M1–M3, M8–M10, M12) showed substantial inter-individual variability in activation timing (Circular SD: 20.68%–44.32%). While their internal structures remained stable, their population prevalence exhibited substantial fluctuations across age groups. These fluctuations, characterized by wide inter-individual variability, suggest that rather than undergoing deterministic neural reorganization, the CNS adopts a “proximal freezing” strategy for basic stability and a “distal freeing” strategy via active sensorimotor exploration to manage redundant degrees of freedom in 4- to 6-year-olds.

## Introduction

Crawling can be categorized into several subtypes, including hands-and-knees crawling, hands-and-feet crawling, and belly crawling [1,2]. Among these, hands-and-knees crawling is a developmental milestone in infants and toddlers [3,4]. Characterized by a quadrupedal base of support and diagonal coordination patterns, it serves as a transitional locomotor mode prior to the acquisition of independent bipedal walking [1,4,5]. During hands-and-knees crawling, the limbs support the trunk with the head oriented forward. The central nervous system (CNS) drives rhythmic flexion and extension of the upper and lower limb joints through coordinated contraction of skeletal muscles, supporting the body and propelling it forward [1,4]. Evidence suggests that crawling influences early neural development [6]. In this task, children must continuously integrate multisensory information to adapt to the environment, and such perception–action coupling provides the foundation for acquiring complex motor skills [7,8].

Previous research on crawling has primarily focused on the primitive features of infant crawling [4,9], the dynamic stability of adult crawling patterns [10,11], or its use as a clinical reference standard for evaluating motor function in populations with brain injuries such as cerebral palsy [12,13]. Collectively, these studies establish crawling as a symmetrical, periodic, and multi-limb rhythmic movement, providing a natural paradigm for investigating how the CNS coordinates whole-body degrees of freedom [4,5,10].

How the CNS coordinates the redundant musculoskeletal system to produce smooth movements remains a central issue in biomechanics and neuroscience [14]. Recent advances in multi-limb biomechanics and multi-joint modeling have extended this fundamental question into broader contexts, with wide-ranging applications in cutting-edge fields such as athletic performance optimization, injury prevention, and clinical rehabilitation [15,16]. In response to the degree of freedom (DOF) redundancy problem proposed by Bernstein, the CNS relies on dimensionality-reduction strategies to lower computational demands [17]. The muscle synergy hypothesis posits that the CNS does not control individual muscles independently; rather, it achieves complex motor behaviors by recruiting a limited number of low-dimensional, pre-organized muscle modules [18–23]. This dimensionality-reduction strategy transforms mechanical degrees of freedom into linear combinations of a small set of muscle activation patterns [11,20,24]. Recent advances in modular control theory distinguish between shared synergies, which address absolute biomechanical constraints, and specific synergies, which reflect individualized compensatory and fine-tuning strategies [23,25]. When managing movements with redundancy, the CNS tends to adopt a hierarchical modular control approach: proximal muscle groups freeze core joint degrees of freedom to provide basic mechanical stability, while distal peripheral muscles are freed to maintain adaptive responsiveness to external perturbations, a strategy defined as “proximal freezing and distal freeing” [4,26–28].

Although the muscle synergy framework is validated in automated skilled movements and abnormal motor patterns [29–31], the neuromotor control of hands-and-knees crawling exhibits variability. This variability arises because the CNS lacks precise feedforward internal models tailored to the current body configuration and multi-limb task dynamics. This variability is directly manifested in the spatial muscle weights and temporal activation coefficients of the synergy modules, indicating that the CNS relies more on sensory feedback than predictive feedforward control [4,11,32,33]. Motor control studies suggest that this variability represents an active sensorimotor exploration strategy by the CNS rather than mere system noise [34]. Therefore, analyzing the distribution patterns of this variability across different muscle synergy modules provides direct neuromechanical evidence for understanding how the developing CNS manages multi-joint DOF redundancy.

Automated movements governed by entrenched spinal-level control mechanisms exhibit spatiotemporal consistency [25,35,36]. In contrast, children aged 4–6 years are in a window of gross motor development and neural control reorganization [37]. During this period, hands-and-knees crawling is retained as a strategy for exploration and adaptation in specific physical environments [38,39]. Systematic empirical evidence concerning the neural control mechanisms underlying the regulation of such tasks in this developmental stage remains limited.

Accordingly, the present study quantitatively examines the inter-limb muscle synergy characteristics of 4–6-year-old children during hands-and-knees crawling [11], focusing on the number of synergies, spatial organization of synergy weights, and spatiotemporal variability of activation profiles [4,11]. The aim is to elucidate how the developing CNS achieves dynamic coordination through proximal freezing and distal freeing under complex multi-limb constraints. This study proposes the following three hypotheses:

- **Hypothesis 1:** The total number of recruited muscle synergies will increase significantly with age, reflecting motor control maturation. Within individuals across consecutive crawling cycles, these synergies will maintain relatively stable spatial structures despite temporal activation variability.
- **Hypothesis 2:** The shared synergies that primarily provide basic postural stability and forward propulsion will demonstrate spatial stability and temporal phase consistency across individuals and across age groups.
- **Hypothesis 3:** The specific synergies involved in compensatory adjustments and distal fine-tuning are expected to exhibit greater spatiotemporal variability across individuals and age groups. Their recruitment patterns are expected to fluctuate across the 4–6-year age range, potentially reflecting an active sensorimotor exploration strategy during development.

## Materials and methods

### Subjects

This study recruited 50 typically developing preschool children (25 boys and 25 girls) from multiple kindergartens in Taiyuan, Shanxi Province, China, between June 15 and November 20, 2024. Participants were equally divided by chronological age into five subgroups (*n* = 10 per group, 5 boys and 5 girls): 48–53, 54–59, 60–65, 66–71, and 72–77 months. One participant in the 66–71 months age group was excluded due to electromyographic (EMG) artifacts, leaving a final sample of 49 participants for subsequent analyses. The detailed demographic characteristics of the participants across the five age groups are summarized in Table 1.

**Table 1:** Demographic characteristics of the participants across age groups.

Age Group	Total (*n)*	Boys	Girls
48–53m	10	5	5
54–59m	10	5	5
60–65m	10	5	5
66–71m	9	4	5
72–77m	10	5	5

Inclusion criteria were: (1) age between 48 and 77 months; (2) ability to perform continuous hands-and-knees crawling independently; (3) full-term birth with normal birth weight and no history of brain injury; (4) no recent trauma or orthopedic surgery.

Exclusion criteria included: (1) any systemic disease affecting motor control during hands-and-knees crawling; (2) interrupted movement rhythm or inability to crawl continuously; (3) use of atypical quadrupedal patterns (e.g., belly crawling or hands-and-feet crawling); (4) poor compliance during testing. The study was conducted in accordance with the ethical principles of the Declaration of Helsinki.

The study protocol was reviewed and approved by the Medical Ethics Committee of Shanxi University (Approval No. SXULL2024082). Following a written agreement from the kindergarten director to participate in the project, the parents or legal guardians of all 4- to 6-year-old children were notified about the study objectives, procedures, risks, and timelines using at least two of the following methods: phone call, email, or in person. Prior to data collection, which occurred between June 15 and November 20, 2024, all parents provided written informed consent. Due to their young age, the children were not required to provide written consent; however, all procedures were explained in child-friendly terms, and the children retained the right to refuse to participate at any time.

### Data acquisition

All data were collected in a temperature-controlled biomechanics laboratory to maintain dry skin and minimize skin impedance. Three-dimensional (3D) kinematic trajectories and multi-channel surface electromyography (sEMG) signals were recorded synchronously during self-paced hands-and-knees crawling [4].

Three-dimensional kinematic data were captured using an infrared optical motion capture system (Vicon, Oxford, UK) at a sampling frequency of 100 Hz. A full-body biomechanical model was applied with 39 reflective markers attached to anatomical landmarks. Marker placements included: the head (LFHD, RFHD, LBHD, RBHD), trunk (LSHD, RSHD, CLAV, STRN, C7, RBAK, T10), pelvis (LASI, RASI, LPSI, RPSI), upper limbs (LUPA, RUPA, LFRM, RFRM, LELB, RELB, LWRA, LWRB, RWRA, RWRB, LFIN, RFIN), and lower limbs (LTHI, RTHI, LKNE, RKNE, LTIB, RTIB, LANK, RANK, LHEE, RHEE, LTOE, RTOE).

Surface electromyography signals were acquired simultaneously using a wireless sEMG system (version 3.18, Noraxon, USA) at a sampling rate of 1500 Hz. A specific 16-channel mapping was utilized, recording bilaterally from the left and right pectoralis major (PM), latissimus dorsi (LD), biceps brachii (BB), triceps brachii (TB), rectus femoris (RF), semitendinosus (ST), gastrocnemius (GAS), and tibialis anterior (TA). Prior to electrode placement, target skin areas were shaved and cleaned with alcohol. Bipolar Ag/AgCl surface electrodes were positioned over the muscle bellies.

Following measurements of body height and body mass and a warm-up period, participants performed continuous hands-and-knees crawling along a straight path of at least 2 m at a self-selected speed. A trial was valid if it contained no pauses, no path deviation, and at least three consecutive crawling cycles. Ten continuous and complete hands-and-knees crawling cycles per participant were extracted for analysis.

### Segmentation of cycles

Continuous hands-and-knees crawling data were segmented into individual cycles. A single hands-and-knees crawling cycle was defined as the interval between two consecutive ground contacts of the right wrist marker (RWRB, positioned on the ulnar side of the wrist aligned with the pinky finger) [4]. To effectively isolate authentic motor trajectories from non-physiological temporal noise in complex kinematic data, our signal processing rationale is grounded in established biomechanical filtering paradigms designed to distinguish true physiological signals from instrumental artifacts [40]. Accordingly, an adaptive cycle segmentation algorithm was applied based on kinematic trajectory data [41]. The three-dimensional (3D) trajectory of the reference marker (RWRB) was filtered using a fourth-order zero-phase Butterworth low-pass filter with a 6 Hz cutoff frequency. The squared magnitude of the resultant 3D velocity (*v*^2^) of this marker was then calculated. For adaptive segmentation, specific percentiles (e.g., 15th, 60th, and 90th percentiles) from the probability distribution of each participant’s *v*^2^ time series were extracted to dynamically generate individualized thresholds for touchdown (TD) and liftoff (LO) events [10]. An event was classified as LO when *v*^2^ exceeded the high-percentile threshold, and as TD when *v*^2^ decreased below the low-percentile threshold. Additionally, the algorithm incorporated a minimum cycle frame constraint and a debouncing time window to exclude transient impacts and skin motion artifacts [42].

### Data preprocessing

Surface electromyography (sEMG) signals were first processed with a 50 Hz digital notch filter to remove power-line interference and a 10--400 Hz band-pass filter. The filtered sEMG signals were subsequently full-wave rectified and low-pass filtered (fourth-order zero-phase Butterworth, 10 Hz cutoff frequency) to extract the linear envelope [20]. To eliminate inter-individual differences in absolute muscle activation intensity, the amplitude of each muscle’s envelope was normalized to its global maximum value recorded during the crawling task [43]. Due to natural variations in absolute cycle duration, each single-cycle envelope was time-normalized to 100 equidistant points (0%--100% of the crawling cycle) using piecewise cubic Hermite interpolating polynomial (PCHIP) interpolation [44]. Finally, the ten selected continuous cycles were concatenated to construct a global sEMG matrix, *V*_global_ (16×1000), for each participant. Additionally, ten individual single-cycle matrices, $V_{t}$ (16×100), were retained for subsequent spatiotemporal stability analyses.

### Muscle synergy extraction and non-negative matrix factorization

With the widespread application of advanced sensor technologies and computational algorithms in motor behavior research, establishing a standardized data extraction and signal processing pipeline has become an essential prerequisite for ensuring scientific rigor and reproducibility [45,46]. In response to this methodological initiative and to ensure the robustness of the sEMG data analysis, dimensionality reduction of the preprocessed surface electromyography (sEMG) data was performed using concatenated non-negative matrix factorization (cNMF) [47,48]. The sEMG data matrix, *V*, was decomposed into a spatial weight matrix, *W*, and a temporal activation coefficient matrix, *C*, according to the model:


$$\begin{array}{c} V=W\times C+E \end{array}$$ (1)

where *E* denotes the residual matrix. The *W* matrix represents the modular spatial structure, while the *C* matrix reflects the temporal dynamics of the neural drive [19,49]. To ensure parameter comparability across participants, the column vectors of *W* were normalized to their maximum value during the spatial template construction stage, and the corresponding rows of *C* were inversely scaled [50,51]. Muscle synergy extraction comprised three steps:

#### (1) Determination of the number of synergy modules (*N*) and data quality control.

To extract muscle synergies, non-negative matrix factorization (NMF) was applied to the global EMG matrix (*V*_global_), which was constructed by temporally concatenating all normalized valid crawling cycles from each participant. Because NMF is a non-convex optimization algorithm and is susceptible to converging to local minima, 20 independent runs with randomly initialized factor matrices (sampled from a uniform distribution) were performed for each candidate number of synergies (*N*). The solution yielding the lowest reconstruction error across the 20 runs was retained as the final model for that *N*. The maximum number of iterations was set to 5,000, and convergence was defined as a change in reconstruction error of less than $1\times 10^{-5}$ for 20 consecutive iterations [10,52]. Reconstruction accuracy was evaluated using the uncentered variance accounted for (VAF):


$$\begin{array}{c} VAF=1-\frac{\sum E^{2}}{\sum V^{2}} \end{array}$$ (2)

where *E* represents the residual error matrix and *V* represents the original EMG data matrix.

To ensure that the extracted synergies adequately captured the essential spatiotemporal features of the EMG signals while minimizing overfitting caused by excessive model complexity, the optimal number of muscle synergies was determined using an incremental threshold-based criterion. Specifically, the candidate number of synergies (*N*) was increased stepwise from 1 to 16. For each *N*, the model with the minimum reconstruction error was used to compute the global VAF (VAF_all_). The optimal number of synergies for each individual was defined as the smallest *N* at which VAF_all_ first reached or exceeded 90%. This threshold has been widely adopted in previous muscle synergy studies to balance reconstruction accuracy and model complexity [11].

#### (2) Single-cycle extraction and intra-individual module alignment.

To evaluate the intra-individual stability of the control strategy, NMF was applied to the independent hands-and-knees crawling cycle matrices ($V_{t}$) of each participant. To avoid local optima, the factorization for each independent cycle was randomly initialized and independently repeated 50 times (with a maximum of 400 iterations per run). The solution yielding the minimum reconstruction error was selected as the specific synergy module (*W*_cycle_) for that cycle [53]. Due to the unordered nature of NMF components, the Hungarian matching algorithm was employed to sort and align the single-cycle modules [54]. Specifically, a cost matrix was constructed using 1−CS (where CS represents cosine similarity) to optimally map and align each single-cycle module (*W*_cycle_) to the global template (*W*_global_) extracted from the participant’s concatenated global data matrix. Once the module mapping was completed, the intra-individual spatial control evaluation metric was quantified as follows: Intra-individual spatial consistency (intra-CS): Defined as the average pairwise cosine similarity of the corresponding synergy modules across all independent cycles within the same participant. This metric was employed to quantify the spatial structural stability of the muscle synergies.

#### (3) Cross-individual module clustering and quantification of spatiotemporal characteristics.

To identify group-level muscle synergies, the spatial weighting vectors (*W*) extracted from all eligible participants were pooled to construct a global spatial vector dataset. Prior to clustering, all vectors were subjected to *L*_2_ normalization to eliminate inter-individual differences in EMG amplitude. The Gap Statistic was then applied to objectively determine the optimal number of clusters (*K*) [55]. Subsequently, *K*-means clustering was performed to partition the normalized spatial vectors into *K* representative group-level muscle synergies [56].

To precisely isolate temporal variability, the group-level spatial weighting matrix (*W*_global_) was fixed as the basis matrix. Each participant’s original EMG data from individual crawling cycles ($V_{t}$) were then projected onto this basis using non-negative least squares (NNLS) to obtain the corresponding temporal activation coefficients ($C_{t}$) [56–58]. Prior to group-level statistical analyses, each reconstructed temporal activation profile was normalized to its peak amplitude. Based on the spatial weight vectors and temporal activation profiles, the spatial stability and temporal variability of each group-level synergy module were quantified using three complementary metrics:

- **Inter-individual spatial consistency (**inter-CS**):** Quantified as the mean pairwise cosine similarity among all spatial weighting vectors assigned to the same cluster. At the participant level, the cosine similarity between each individual’s spatial weighting vector and the corresponding cluster centroid was also calculated.
- **Temporal waveform similarity (Pearson’s** *r***):** For each cluster, the temporal activation profiles (*C*) associated with the corresponding spatial vectors were extracted, and the mean pairwise Pearson correlation coefficient was calculated to evaluate the similarity of the activation waveform morphology.
- **Temporal phase dispersion (Circular SD):** The center of activity (CoA) for each temporal activation profile was determined using vector summation in polar coordinates. The temporal phase dispersion (Circular SD) was then computed across all CoA values within each cluster and expressed as a percentage of the movement cycle (SD/2π×100%), providing a robust measure of temporal phase variability.

Finally, to characterize the anatomical composition and facilitate the biomechanical interpretation of each group-level synergy, dominant muscles were identified from the normalized group-level spatial weighting matrix. A muscle was classified as dominant if its relative weight in the normalized cluster centroid matrix exceeded 0.3 [4,59]. Based on this, modules with a population prevalence greater than 70% [58] and a coefficient of variation (CV) of prevalence across age groups of less than 0.3 were defined as shared synergies [56]. Modules that did not simultaneously satisfy both criteria, namely an overall population prevalence >70% and a coefficient of variation (CV) of prevalence <0.30 across age groups, were classified as specific synergies.

### Statistical analysis

The significance level was set at α=0.05. Continuous variables (*N*, CS, and *r*) are reported using descriptive statistics. Temporal phase dispersion is reported as the temporal phase dispersion (Circular SD). The Kruskal-Wallis test was used to evaluate the main effect of age group (G1–G5) on the aforementioned parameters. The effect size ($\eta^{2}$) was calculated according to the model 3:


$$\begin{array}{c} \eta^{2}=\frac{H-k+1}{n-k} \end{array}$$ (3)

where *H* is the Kruskal-Wallis statistic, *k* is the number of groups, and *n* is the total sample size. When a statistically significant main effect was detected, post-hoc pairwise comparisons were performed using the Mann-Whitney *U* test, with *p*-values adjusted via the Benjamini-Hochberg false discovery rate (FDR-BH) method. For these comparisons, the effect size was reported as the rank-biserial correlation (*r*_rb_).

In the inter-individual clustering analysis, the CS, *r*, and Circular SD of each module were reported to quantitatively assess the spatiotemporal stability characteristics of the different synergy modules. Using these individual-level metrics as dependent variables and age group as the independent variable, non-parametric Kruskal-Wallis H tests were performed. If the main effect of age was significant (*p* < 0.05), Mann-Whitney U tests were further applied for post-hoc pairwise comparisons, followed by FDR-BH correction.

To assess module robustness against algorithmic over-segmentation, a repeated split-half validation was conducted. The 49 participants were randomly partitioned into two independent subsets (*n* = 24 and 25). Independent *K*-means clustering (*K* = 12) was applied to each subset, and the resulting centroids were aligned to the full-cohort reference using the Hungarian algorithm. This random partitioning and matching procedure was repeated 100 times. Spatial reliability was quantified as the mean cosine similarity (Split-half CS) between the matched subset centroids across all 100 iterations.

To quantify the sampling uncertainty of module prevalence estimates, participant-level bootstrap resampling was performed within the full cohort and separately within each age group. For each module, 10,000 bootstrap samples were generated with replacement, and the prevalence was recalculated for each resample. The 2.5th and 97.5th percentiles of the resulting bootstrap distribution were used to construct 95% confidence intervals.

## Results

### Number of muscle synergies and intra-individual spatiotemporal control characteristics

Concatenated non-negative matrix factorization (cNMF) was utilized to establish a global reference template, which was combined with independent single-cycle extraction to quantify the spatial weights and temporal variability of muscle synergies during continuous hands-and-knees crawling.

In the final 49 valid participants, the mean number of muscle synergies extracted from the 16-channel sEMG signals was 7.82±1.05 under the VAF ≥90% threshold. The distribution of module numbers was as follows: *N* = 8 in 40.82% (20 participants), *N* = 7 in 24.49% (12 participants), *N* = 9 in 20.41% (10 participants), *N* = 6 in 8.16% (4 participants), *N* = 10 in 4.08% (2 participants), and *N* = 5 in 2.04% (1 participant) (Fig 1A).

**Fig 1:**
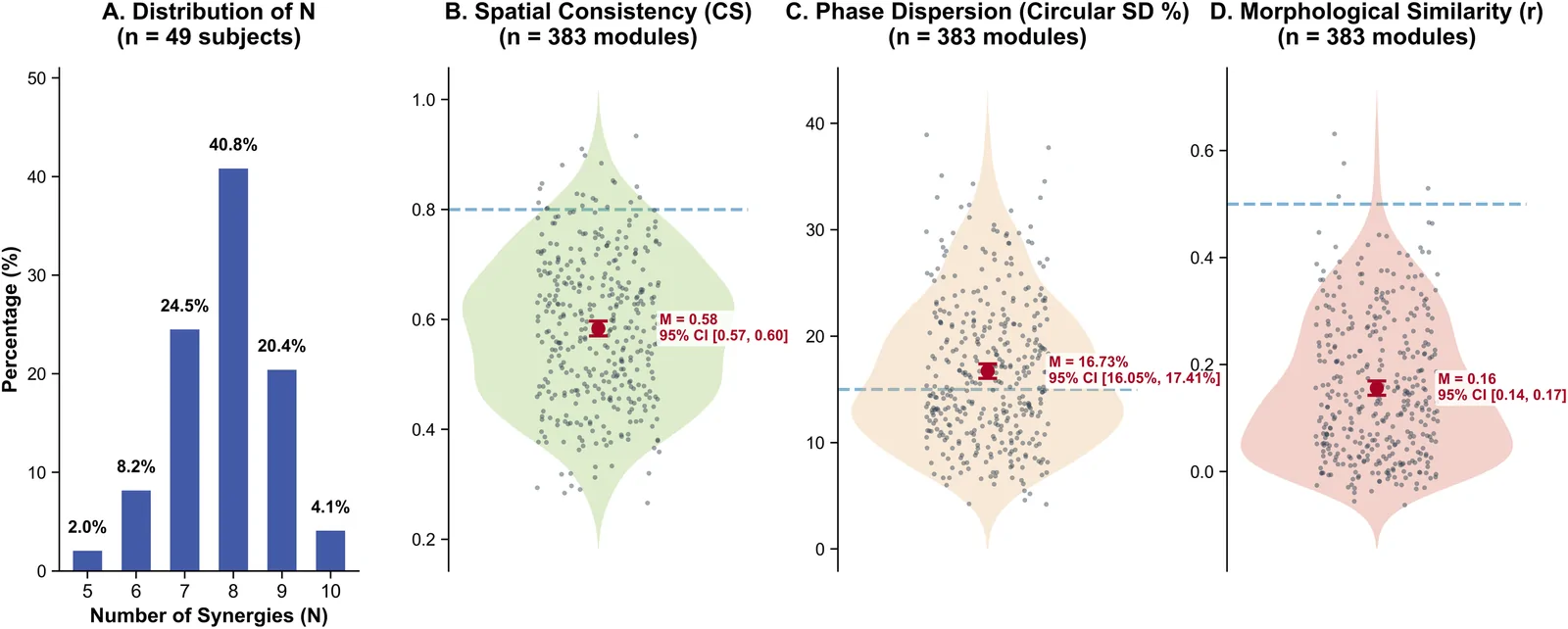
Distribution of muscle synergy module numbers (*N*) and group-level spatiotemporal variability metrics in preschool children during hands-and-knees crawling. (A) Percentage distribution of synergy numbers. (B) Spatial consistency (CS). (C) Temporal phase dispersion (Circular SD). (D) Morphological similarity (*r*).

The mean intra-individual spatial consistency (CS) across continuous hands-and-knees crawling cycles was 0.58 (95% CI: [0.57, 0.60]) (Fig 1B). The mean temporal phase dispersion of activation coefficients (*C*), quantified by the temporal phase dispersion (Circular SD) of the center of activity (CoA), was 16.73% of the crawling cycle (95% CI: [16.05%, 17.41%]) (Fig 1C). The mean temporal pattern similarity, calculated using Pearson’s correlation coefficient (*r*), was 0.16 (95% CI: [0.14, 0.17]) (Fig 1D).Compared with the high intra-individual consistency reported during natural-paced adult walking [25], these relatively low spatial and temporal similarity values indicate greater cycle-to-cycle variability in the present preschool cohort, which will be further contextualized from a developmental perspective in the Discussion section.

A representative sample (*N* = 7) across ten consecutive cycles demonstrated a spatial consistency (CS) (Fig 2).

**Fig 2:**
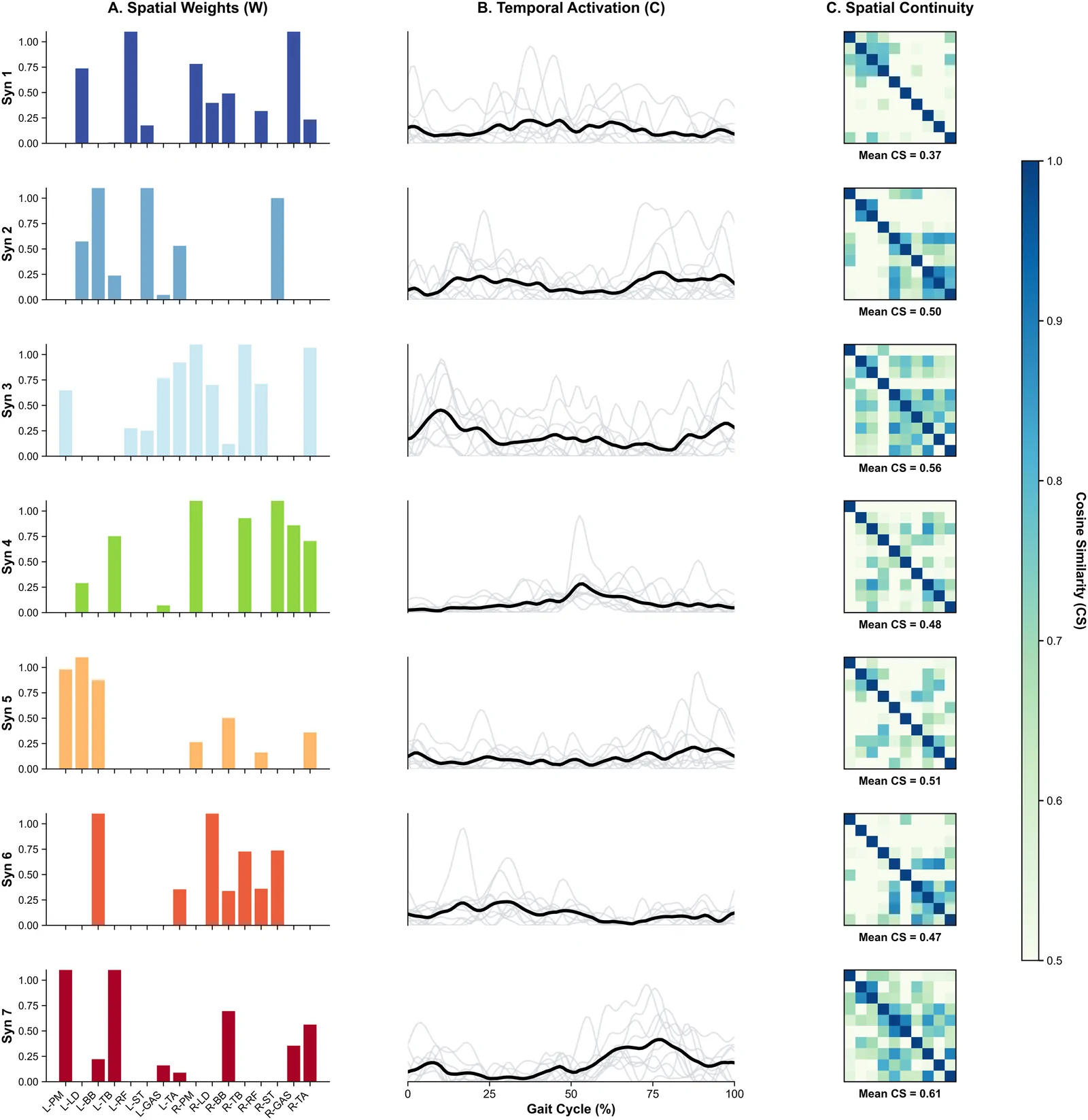
Representative intra-individual spatiotemporal features of muscle synergies (*N* = 7) during hands-and-knees crawling in a typical child. (A) Spatial weight matrix (*W*). (B) Temporal activation curves (*C*). (C) Heatmap of spatial consistency across 10 consecutive crawling cycles.

### Inter-individual clustering and spatiotemporal characteristics analysis of muscle synergies

Global spatial weight matrices from the 49 participants were subjected to K-means clustering, according to the Gap Statistic criterion, the optimal number of clusters was 12 (M1–M12). The representative anatomical structures and average temporal activation waveforms of these modules are shown in Fig 3, and quantitative statistics are presented in Table 2.

**Fig 3:**
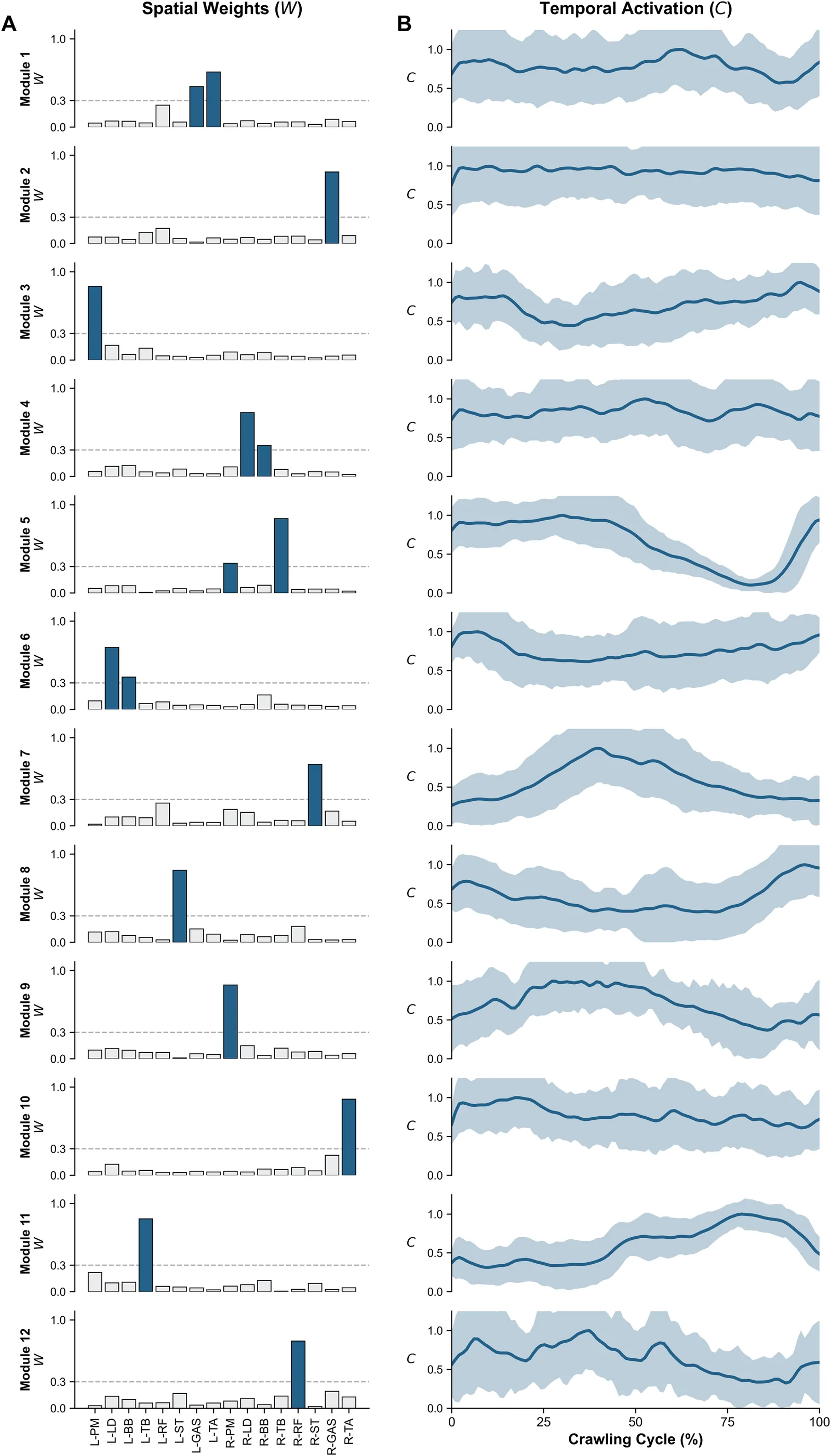
Spatiotemporal organization of group-level muscle synergies (*M* = 12) during hands-and-knees crawling in preschool children. (A) Spatial weight vectors (*W*), showing relative recruitment intensity across the 16 target muscles (the gray dashed line indicates the *W* = 0.3 threshold; dominant muscles are marked in dark colors). (B) Temporal activation patterns (*C*). Solid lines represent mean activation profiles across participants, with shaded areas indicating the standard deviation (±1 SD). The x-axis is normalized to a single hands-and-knees crawling cycle (0%–100%).

**Table 2:** Cross-individual spatiotemporal characteristics and hypothesized biomechanical functions of muscle synergies (*M* = 12) during hands-and-knees crawling in preschool children.

Module	Subjects	Bootstrap	CV of	Split-half CS	Spatial similarity	Phase Dispersion	Dominant Muscles	Hypothesized Biomechanical
	(*n*, %)	95% CI	Prevalence	(Mean ± SD)	(CS, mean ± SD)	(circular SD, %)	(*W* > 0.3)	Function
Module 1	33 (67.35)	[53.1%, 79.6%]	0.22	0.97±0.02	0.70±0.18	39.16%	L-GAS, L-TA	Left lower limb: Distal fine-tuning & toe clearance
Module 2	15 (30.61)	[18.4%, 42.9%]	0.76	0.77±0.22	0.75±0.13	44.32%	R-GAS	Right lower limb: Distal fine-tuning
Module 3	32 (65.31)	[51.0%, 77.6%]	0.17	0.98±0.01	0.78±0.13	22.63%	L-PM	Left upper limb: Postural compensation & stabilization
Module 4	38 (77.55)	[65.3%, 87.8%]	0.25	0.98±0.01	0.71±0.17	35.10%	R-LD, R-BB	Right upper limb: Propulsion & proximal stabilization
Module 5	43 (87.76)	[77.6%, 95.9%]	0.10	0.99±0.005	0.86±0.09	12.42%	R-PM, R-TB	Right upper limb: Anti-gravity support
Module 6	36 (73.47)	[61.2%, 85.7%]	0.18	0.97±0.02	0.69±0.19	28.34%	L-LD, L-BB	Left upper limb: Propulsion & proximal stabilization
Module 7	41 (83.67)	[73.5%, 93.9%]	0.23	0.97±0.03	0.68±0.19	17.36%	R-ST	Right lower limb: Propulsion & pelvic stabilization
Module 8	36 (73.47)	[61.2%, 85.7%]	0.33	0.98±0.01	0.79±0.12	21.36%	L-ST	Left lower limb: Postural compensation
Module 9	18 (36.73)	[24.5%, 51.0%]	0.72	0.95±0.08	0.80±0.10	20.68%	R-PM	Right upper limb: Postural compensation & stabilization
Module 10	27 (55.10)	[40.8%, 69.4%]	0.52	0.98±0.01	0.84±0.10	38.62%	R-TA	Right lower limb: Distal fine-tuning
Module 11	42 (85.71)	[75.5%, 93.9%]	0.16	0.99±0.006	0.80±0.11	13.73%	L-TB	Left upper limb: Anti-gravity support
Module 12	13 (26.53)	[14.3%, 38.8%]	0.50	$0.45\pm 0.29^{*}$	0.72±0.11	22.90%	R-RF	Right lower limb: Flexion-related propulsion

*Note: Module 12 exhibited lower split-half consistency, reflecting higher inter-individual variability.

The 12 group-level muscle synergies exhibited distinct differences in population prevalence and spatial consistency, as quantified by the mean pairwise cosine similarity (Mean CS) within each cluster (Fig 4A and 4B). Five modules (M4, M5, M6, M7, and M11) were identified as core shared synergies, exhibiting high population prevalence ranging from 73.47% to 87.76%, robust developmental stability across the five age groups (all CVs of prevalence < 0.3), and stable spatial organization (Mean CS values ranging from 0.68 to 0.86). Specifically, M11 covered 85.71% of participants (CV = 0.16; Mean CS = 0.80 ± 0.11) and was dominated by the left triceps brachii (L-TB). Modules M4 and M6 covered 77.55% (CV = 0.25; Mean CS = 0.71 ± 0.17) and 73.47% (CV = 0.18; Mean CS = 0.69 ± 0.19) of participants, respectively, and exhibited bilaterally symmetric dominant muscle patterns: M4 was dominated by the right latissimus dorsi and right biceps brachii (R-LD, R-BB), while M6 was dominated by their left counterparts (L-LD, L-BB). M5 showed the highest prevalence, being identified in 87.76% of participants (CV = 0.10; Mean CS = 0.86 ± 0.09), and was primarily driven by the right triceps brachii and right pectoralis major (R-TB, R-PM). Module M7 was present in 83.67% of participants (CV = 0.23; Mean CS = 0.68 ± 0.19) and was dominated by the right semitendinosus (R-ST). In contrast, the remaining specific synergies (M1–M3, M8–M10, and M12) were classified as such either due to lower overall population prevalence (< 70%) or significant developmental fluctuations across age groups (CV ≥ 0.3). To rigorously test the stability of these group-level clusters and address potential over-segmentation concerns regarding low-prevalence modules, a repeated split-half validation was conducted. The results demonstrated that while core shared synergies (M4–M7, M11) and most specific modules (e.g., M1, M3, M8–M10) exhibited exceptionally high spatial reliability (mean CS > 0.95), Module 12 (M12) showed a notably lower mean split-half cosine similarity (0.451 ± 0.295), indicating high sensitivity to sample partitioning.

**Fig 4:**
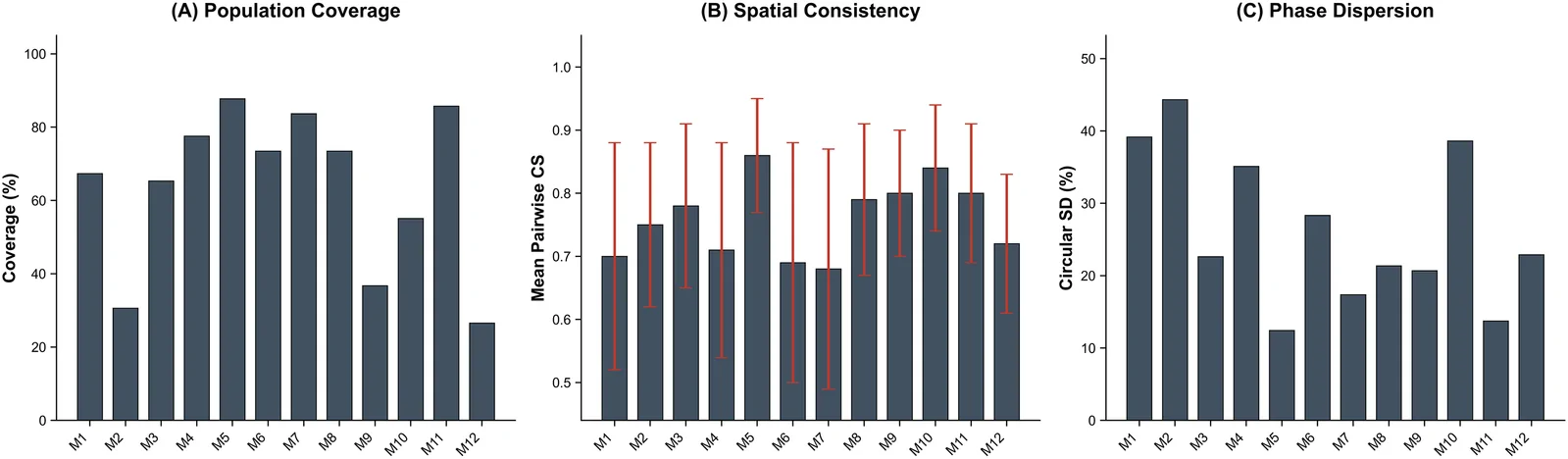
Group-level consistency and spatiotemporal variability of muscle synergies across individuals. (A) Population coverage of each module. (B) Intra-module spatial similarity (mean pairwise CS). (C) Temporal phase dispersion (Circular SD) of temporal activation phases.

Among them, M8 had a prevalence of 73.47% (CV = 0.33; Mean CS = 0.79 ± 0.12) and was dominated by the left semitendinosus (L-ST). Modules M1 and M3 were observed in 67.35% (CV = 0.22) and 65.31% (CV = 0.17) of participants, with Mean CS values of 0.70 ± 0.18 and 0.78 ± 0.13, respectively; M1 was characterized by the left tibialis anterior and left gastrocnemius (L-TA, L-GAS), while M3 was dominated by the left pectoralis major (L-PM). Module M10 covered 55.10% of participants (CV = 0.52; Mean CS = 0.84 ± 0.10) and was dominated by the right tibialis anterior (R-TA). In comparison, M2, M9, and M12 showed relatively low prevalence in the study population, ranging between 26.53% and 36.73% (CVs ranging from 0.50 to 0.76). For example, M2 was observed in 30.61% of participants (CV = 0.76; Mean CS = 0.75 ± 0.13), with its dominant muscle corresponding to the right gastrocnemius (R-GAS).

Analysis of the circular distribution of Center of Activity (CoA) values, aligned using non-negative least squares (NNLS), revealed that all 12 synergy modules exhibited temporal variability (Fig 4C). The core shared synergies hypothesized to contribute to basic body support and propulsion (M4, M5, M6, M7, and M11) exhibited moderate to low inter-individual phase dispersion, with Circular SD values of 35.10%, 12.42%, 28.34%, 17.36%, and 13.73%, respectively. Conversely, among the specific synergies associated with distal fine-tuning and compensatory adjustments, M1, M2, and M3 displayed Circular SD values of 39.16%, 44.32%, and 22.63%, respectively, while the remaining specific modules (M8, M9, M10, and M12) showed Circular SD values of 21.36%, 20.68%, 38.62%, and 22.90%, respectively.

### Age-related differences in muscle synergy control strategies

At the macroscopic level, age group had a significant main effect on the number of extracted muscle synergies (N) (*p* = 0.011). Post-hoc Mann-Whitney U tests with false discovery rate (FDR) correction further revealed that the 6-year-old group (G5) exhibited a significantly greater number of muscle synergies than the 4-year-old group (G1) (adjusted *p* = 0.035, rank-biserial correlation *r*_rb_ = 0.64).

At the modular level (as shown in Fig 5), for the shared synergies (M4, M5, M6, M7, and M11), Kruskal-Wallis *H* tests revealed no statistically significant main effects of age group on spatial consistency (CS), temporal pattern similarity (*r*), or phase dispersion (Circular SD) (all *p* > 0.05). For the specific synergies (M1–M3, M8–M10, and M12), no significant main effect of age group was observed on their internal spatial consistency (Mean CS) across G1–G5 (all *p* > 0.05). Similarly, temporal waveform similarity (Pearson’s *r*) did not differ significantly across age groups for any specific synergy (all *p* > 0.05). Regarding temporal phase dispersion (Circular SD), a significant age effect was detected only for M8 (*p* = 0.034). Post-hoc pairwise comparisons with false discovery rate (FDR) correction revealed a significant difference exclusively between G1 and G4 (adjusted *p* = 0.020, rank-biserial correlation *r*_rb_ = 0.96), with no other comparisons reaching statistical significance. The remaining six specific modules showed no significant age-related differences in Circular SD (all *p* > 0.05).

**Fig 5:**
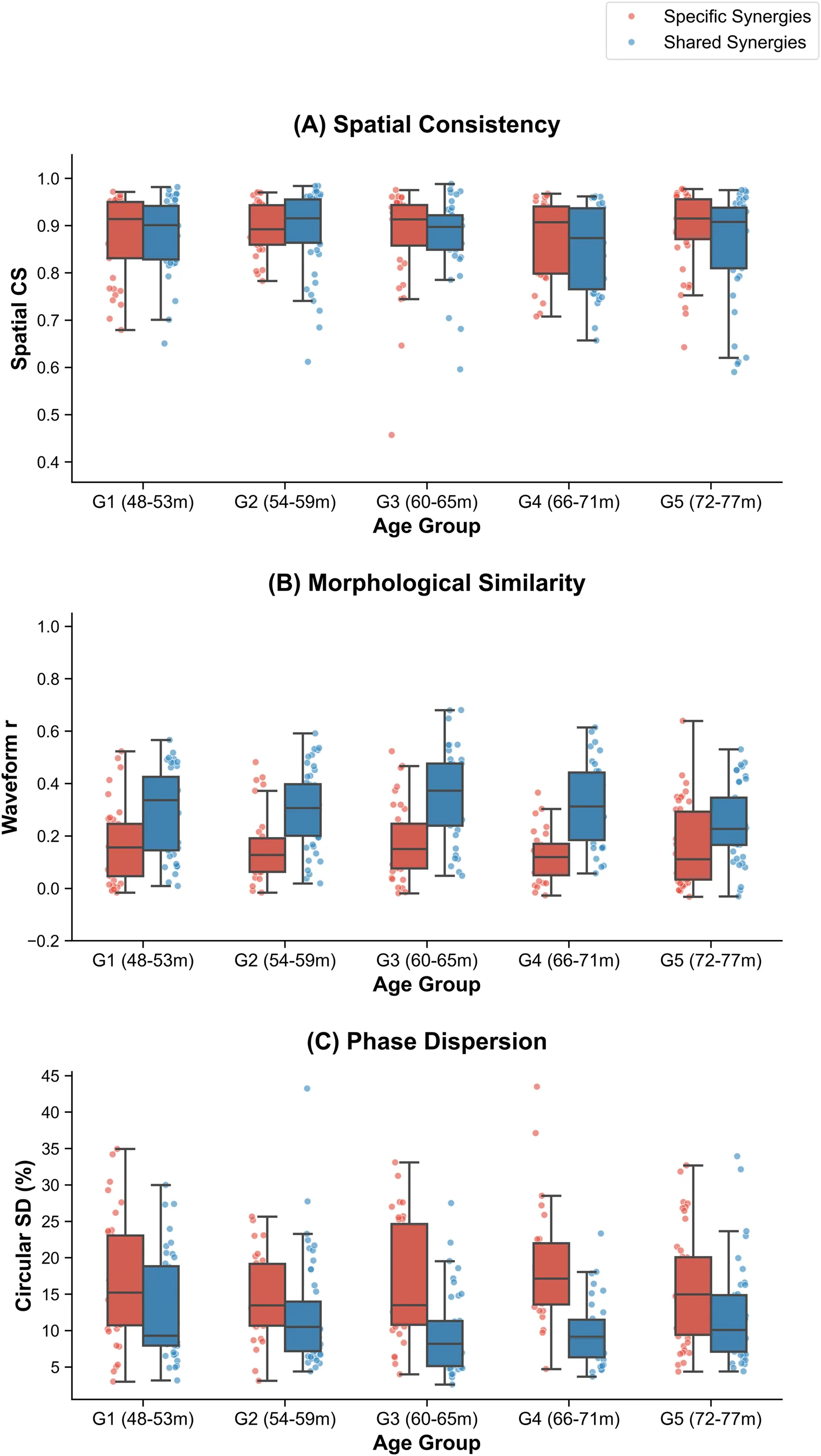
Developmental profiles of shared and specific synergies across age groups. (A) Spatial consistency (CS). (B) Morphological similarity (*r*). (C) Temporal phase dispersion (Circular SD).

Compared with the core shared synergies, the population prevalence (detection rate) of the seven specific synergies exhibited highly heterogeneous trajectories across the G1–G5 age groups. To account for the variance inherent in our sample sizes (n=9--10 per group), we computed non-parametric bootstrap 95% confidence intervals (CIs) for these prevalence estimates. Specifically, the prevalence of M1 demonstrated an overall increasing trend, rising from 50.0% in G1 (5/10, 95% CI: [20.0%, 80.0%]) to 90.0% in G5 (9/10, 95% CI: [70.0%, 100.0%]). M2 displayed a fluctuating pattern: its prevalence was 10.0% in G1 (1/10, 95% CI: [0.0%, 30.0%]), peaked at 66.7% in G4 (6/9, 95% CI: [33.3%, 100.0%]), and appeared at 40.0% in G5 (4/10, 95% CI: [10.0%, 70.0%]). M9 exhibited multi-phasic fluctuations, dropping completely to 0.0% in G4 (0/9, 95% CI: [0.0%, 0.0%]) before partially recovering to 30.0% in G5 (3/10, 95% CI: [0.0%, 60.0%]). M10 initially decreased from 80.0% in G1 to 30.0% in G2, remained relatively low through G4 (33.3%, 95% CI: [0.0%, 66.7%]), and underwent a recovery to 90.0% in G5 (9/10, 95% CI: [70.0%, 100.0%]). The wide confidence intervals associated with these fluctuations indicate substantial inter-individual variability within each age group. In contrast, the prevalence of M3, M8, and M12 fluctuated irregularly without demonstrating any consistent trends.

## Discussion

In the present study, an average of 7.82±1.05 muscle synergies were extracted from children during continuous hands-and-knees crawling. This number is substantially higher than the 4–5 synergy modules typically reported for human bipedal walking [20]. These findings indicate that hands-and-knees crawling, as a quadrupedal locomotor pattern characterized by higher degrees of freedom [10], requires the coordination of diagonal limb support and propulsion between the upper and lower extremities, which may contribute to a more differentiated organization of muscle-activation patterns. Although the mean number of recruited muscle synergies increased modestly from approximately 7–8 (specifically, from 7.70±0.82 in G1 to 8.80±0.79 in G5) over the 24-month span, this between-group difference was associated with a medium-to-large effect size (*r*_rb_ = 0.64). From a neuromotor control perspective, this incremental expansion may reflect a greater differentiation and diversity of muscle-activation patterns during preschool development. It mirrors the progressive transition from rigid, whole-body co-activation toward a more flexible motor repertoire, indicating an evolving capacity of the central nervous system to manage redundant degrees of freedom as sensorimotor integration matures. The relatively high number of extracted synergies at the individual level warrants cautious interpretation of the dimensionality reduction achieved by NMF. In the present study, an average of 7.82±1.05 synergies was required to reconstruct the 16-channel sEMG data under the 90% VAF criterion. This relatively stringent criterion prioritizes reconstruction fidelity and may require additional components to account for residual EMG variance. Therefore, the extracted individual synergies should not be interpreted solely as direct evidence of independently controlled neural commands [60], and the possibility of algorithmic over-decomposition cannot be completely excluded. In addition, the use of concatenated NMF across ten continuous crawling cycles without cycle averaging preserved cycle-to-cycle variability, which may also have contributed to the relatively high individual synergy number.

At the group level, the 12 synergies were derived by clustering spatial weighting patterns across 49 participants and therefore represent a population-level repertoire of recurring muscle-activation patterns. This repertoire was not simply a one-muscle/one-synergy decomposition, as several highly prevalent modules comprised multiple functionally related muscles. Single-muscle-dominant modules were therefore interpreted cautiously as activation patterns potentially contributing to distal fine-tuning rather than as direct evidence of independently controlled neural commands.

Comparison with previous studies on infant crawling reveals that, although both confirm that muscle synergies serve as the core control strategy for quadrupedal locomotion, substantial differences exist in the degree of modular differentiation. For example, Xiong et al. reported that infants with a mean age of approximately 13 months typically recruited fewer synergies during crawling (an average of approximately 3) [4]. In contrast, the present study extracted a significantly larger number of synergy modules (an average of nearly 8) in 4- to 6-year-old children. One possible explanation for this difference is that the immature infant nervous system tends to employ large-scale muscle co-activation to “freeze” whole-body degrees of freedom in exchange for postural stability [61]. By contrast, 4- to 6-year-old children possess more advanced sensorimotor integration capabilities, potentially enabling them to “free” degrees of freedom through greater diversity of muscle-activation patterns [62].

Furthermore, the data revealed that the intra-individual spatial consistency (Mean CS) was 0.58, the temporal waveform similarity (Pearson’s *r*) was only 0.16, and the temporal activation phase dispersion (Circular SD) reached 16.73%. Although the mean intra-individual spatial consistency (CS = 0.58) was lower than values commonly reported for mature locomotor patterns, it should be interpreted as relative rather than absolute spatial stability within the present pediatric sample. This combination of low temporal similarity and moderate spatial consistency suggests that while maintaining the global spatial framework of the crawling pattern, children rely on the continuous fine-tuning of muscle activation phases and weights to ensure locomotor continuity and dynamic postural stability [10]. Importantly, this cycle-to-cycle variability does not necessarily indicate a lack of stability in the underlying muscle coordination, but rather reflects variability in the expression of the identified synergy patterns across repeated crawling cycles.

The 12 group-level muscle synergies (M1–M12) extracted via K-means clustering exhibited a distinct functional division into ’core shared’ and ’specific fine-tuning’ modules [11], demonstrating the dimensionality-reduction control strategy employed by the human central nervous system (CNS) when confronting complex tasks [63]. The results demonstrate that the central nervous system (CNS) adopts a two-layer control architecture when managing the degree of freedom (DOF) redundancy inherent in hands-and-knees crawling.

On one hand, this architecture relies on five spatially stable shared synergies (M4, M5, M6, M7, and M11) characterized by high population prevalence (73.47%–87.76%) and relatively consistent spatial organization (Mean CS > 0.68). Their dominant muscles are primarily concentrated in the trunk and proximal extremities (e.g., latissimus dorsi, pectoralis major, triceps brachii, and semitendinosus), which are hypothesized to contribute to anti-gravity support and forward propulsion, thereby maintaining baseline mechanical stability [10]. On the other hand, seven highly variable specific synergies (M1–M3, M8–M10, and M12) exhibit lower population prevalence, with their dominant muscles predominantly involving distal muscle groups (e.g., bilaterally the tibialis anterior [L/R-TA] and gastrocnemius [L/R-GAS]). These specific synergy modules possess exceptionally high temporal control variability and are hypothesized to contribute to fine-tuning limb trajectories during the swing phase, cushioning-related control during ground contact, and individualized postural compensation [62]. This control strategy supports the proximal freezing and distal freeing dimensionality-reduction principle–-locking specific joint degrees of freedom to simplify control while releasing others to preserve flexibility–-providing quantitative electromyographic evidence for how developing children coordinate multi-joint redundancy.

Each muscle synergy was associated with specific biomechanical functions [25,64,65]. Based on established muscle anatomy and the biomechanical demands of hands-and-knees crawling, the following functional interpretations are proposed [10]. During this locomotor pattern, the triceps brachii, serving as the primary elbow extensor, plays a critical role in counteracting gravity to prevent forelimb collapse during weight-bearing [4]. The pectoralis major not only contributes to shoulder adduction and flexion but also effectively stabilizes the connection between the trunk and upper limbs via its clavicular and sternocostal fibers [66]. Accordingly, the synergy module dominated by the right triceps brachii and pectoralis major (M5) and the module dominated by the left triceps brachii (M11) are likely associated with robust anti-gravity support of the upper limbs [10]. These modules are hypothesized to contribute to elbow extension torque during the stance phase and may also be involved in limiting excessive shoulder abduction and maintaining an elevated trunk posture. Furthermore, the latissimus dorsi, a broad muscle connecting the spine, pelvis, and humerus, is responsible for shoulder extension and adduction, whereas the biceps brachii facilitates forearm flexion and assists in shoulder flexion [67]. Their coordinated action enables forelimb protraction during the swing phase and stabilization of the glenohumeral joint during stance [13]. Consequently, the bilaterally symmetric modules dominated by the latissimus dorsi and biceps brachii (M4 and M6) are hypothesized to contribute to proximal shoulder-girdle stabilization and forelimb propulsion. In the lower limbs, the semitendinosus, a primary hamstring muscle spanning both the hip and knee joints, contributes to hip extension and knee flexion, serving as a core source of propulsive power and pelvic postural regulation [68]. Thus, the shared module dominated by the right semitendinosus (M7) is hypothesized to be closely associated with hindlimb propulsion and dynamic pelvic stabilization during asymmetrical crawling.

In contrast to the “basic support framework” constructed by the core shared synergies, the specific synergies (e.g., M1–M3, M8–M10, and M12), which are characterized by lower prevalence and higher temporal activation variability, are postulated to perform fine-tuning functions to address environmental perturbations and individualized postural compensation. The tibialis anterior (TA) primarily controls ankle dorsiflexion, whereas the gastrocnemius (GAS) governs ankle plantarflexion and assists in knee flexion [69,70]. Although the ankle is not the primary weight-bearing or propulsive joint during hands-and-knees crawling, lower-leg and foot movements are essential for maintaining overall dynamic balance and preventing toe drag [13,62,71]. Therefore, the module co-dominated by the left TA and GAS (M1), along with the modules individually dominated by the right TA (M10) and right GAS (M2), are likely associated with fine motor control of the distal limbs during the swing phase. Additionally, certain specific synergies may reflect asymmetrical center-of-mass shifts and localized compensatory strategies [72]. For instance, the module dominated by the left pectoralis major (M3) and the module dominated by the left semitendinosus (M8, functionally distinct from the core shared right semitendinosus module M7) may play crucial roles in localized fine-tuning to counteract asymmetrical perturbations [72]–-such as the tendency for lateral trunk tilt induced by contralateral limb swing–-thereby assisting in maintaining the horizontal stability of the shoulder girdle and pelvis through the compensatory recruitment of contralateral muscles [73].

This anatomically grounded functional differentiation suggests that the neural control system tends to translate complex biomechanical demands directly into low-dimensional motor modules [18]. By “freezing” the degrees of freedom at joints closer to the trunk (e.g., the shoulder and hip), the CNS establishes a robust support framework. This strategy simplifies the neural control of hands-and-knees crawling by eliminating the need for independent computational control at every individual joint [4,11].

In the spatiotemporal domain, shared and specific synergies exhibited marked differences in control characteristics during hands-and-knees crawling. The five shared synergy modules showed no significant main effects of age group (G1–G5) on either internal spatial consistency (Mean CS) or temporal parameters [74,75]. This stability reflects fundamental control patterns consolidated at the spinal level. In line with the two-level central pattern generator (CPG) theory, these stable proximal shared modules are globally activated during hands-and-knees crawling to implement proximal freezing, providing reliable physical constraints for the body [10,76,77]. In contrast, the specific synergies responsible for distal fine-tuning of the lower leg and ankle (e.g., M1 co-dominated by the left tibialis anterior and gastrocnemius, M2 dominated by the right gastrocnemius, and M10 dominated by the right tibialis anterior) not only exhibited lower population prevalence but also displayed exceptionally high temporal phase variability, with Circular SD values reaching 39.16%, 44.32%, and 38.62%, respectively. Drawing upon motor development and neural control theories, this high variability in distal control is unlikely to represent mere system error or noise. Recent comparative studies investigating distal muscle control mechanisms and crossed neuromuscular features in children versus adults have confirmed that distal motor pathways and inter-limb neural interactions during development possess plasticity and regulatory characteristics fundamentally distinct from those of adults. Applying this developmental neuromechanical perspective to the present study, the pronounced temporal phase shifts observed in the specific synergies responsible for distal fine-tuning may reflect active motor exploration by the central nervous system (CNS) [32,36]. However, this interpretation should be considered cautiously, as part of the observed temporal phase dispersion may also arise from uncontrolled variations in self-paced crawling speed across children and age groups. Differences in crawling speed may alter the relative timing of muscle activation within the crawling cycle and thereby contribute to the observed phase dispersion. Given the potentially limited precision of feedforward internal models tailored to the current quadrupedal posture, the CNS may maintain proximal stability while actively “freeing” redundant degrees of freedom at the distal segments. This proximal-stability and distal-fine-tuning partnership may therefore represent an important strategy for handling complex motor tasks [26].

Unlike the stable proximal shared modules, the prevalence of specific synergies showed heterogeneous recruitment patterns across age groups [63]. Notably, in the G4 age group (66–71 months), the prevalence of the distal regulatory module M2 reached 66.7% whereas M9 was not detected (0%). Concurrently, a statistically significant difference in temporal phase dispersion (Circular SD) for M8 was observed between G1 and G4 (FDR-adjusted *p* = 0.020). However, the wide bootstrap confidence intervals reported in the Results (e.g., the CI for M2 in G4 ranged from 33.3% to 100.0%) indicate substantial uncertainty in these prevalence estimates, particularly given the small sample sizes within individual age groups. Therefore, these fluctuations may reflect substantial inter-individual variability in the recruitment of specific muscle-activation patterns and may be consistent with ongoing sensorimotor exploration during development. To adapt to complex mechanical constraints, the CNS may dynamically adjust the recruitment of specific muscle-activation patterns across individuals to support this developmental process [78,79]. Upon entering G5 (72–77 months), the total number of extracted muscle synergies (*N*) was significantly greater than in G1 (48–53 months) (adjusted *p* = 0.035), accompanied by a high prevalence of distal fine-tuning modules such as M10 (90.0%, 95% CI: [70.0%, 100.0%]). This pattern may indicate increasing differentiation and diversity of muscle-activation patterns with age, potentially allowing greater flexibility in the recruitment of distal muscle synergies during sensorimotor development [80,81].

It is also worth noting that Module 12 (M12, dominated by the right rectus femoris) exhibited lower split-half spatial reliability (mean CS=0.451±0.295). This finding indicates that M12 was sensitive to participant sampling and should therefore be interpreted cautiously. Rather than treating M12 as a robust population-level motor module, its identification may reflect an individual or sample-dependent recruitment pattern that requires further verification in larger and longitudinal cohorts.

### Limitations

A methodological strength of this study lies in the application of concatenated non-negative matrix factorization (cNMF), which avoids the smoothing effect of traditional single-cycle averaging on intra-cycle signal variance [82]. This approach enables the quantification of temporal phase shifts between cycles arising from sensory feedback-mediated fine-tuning [25].

Nevertheless, several limitations should be acknowledged. First, the cross-sectional design restricts causal inferences regarding the longitudinal development of motor control strategies. Additionally, variations in children’s daily physical activity exposure (e.g., climbing habits or frequency of sports training) may act as potential confounding factors. Second, the investigation of neural control strategies relied primarily on surface electromyography (sEMG) signals without synchronous kinetic validation parameters. Consequently, the mechanical contributions of the degrees of freedom release strategy to movement smoothness and energy dissipation could not be directly quantified. Third, a methodological limitation exists regarding the time normalization procedure. The electromyographic (EMG) signals for each complete crawling cycle were uniformly time-normalized to 100 data points. Because the present study primarily focused on global muscle synergy patterns, the crawling cycles were not explicitly partitioned into distinct stance and swing phases. Consequently, this global normalization strategy may obscure potential developmental differences in the stance-to-swing temporal ratio across age groups. Future research should incorporate phase-specific normalization strategies alongside comprehensive spatiotemporal kinematic analyses to further elucidate the temporal characteristics of motor control. Fourth, the current investigation solely monitored major superficial muscles, precluding the inclusion of deep core stabilizing muscles (e.g., the transversus abdominis and multifidus) and shoulder girdle stabilizers (e.g., the serratus anterior). Considering the critical roles these muscles play in maintaining quadrupedal posture and dynamic stability, the muscle synergy patterns extracted herein may not encompass the complete repertoire of neuromotor control strategies employed by the central nervous system (CNS) during children’s crawling. Furthermore, several potentially relevant task- and participant-related covariates were not available for systematic statistical adjustment in the present study. Although body height and body mass were measured, these variables were not incorporated as covariates in the original statistical analyses. More importantly, while crawling was performed at a self-selected speed, key task-specific kinematic variables such as crawling speed and stride length were not systematically quantified. Consequently, the observed age-group differences should not be interpreted as effects that are completely independent of individual differences in body size or motor performance. In particular, differences in self-selected crawling speed may have contributed to the temporal dispersion observed for some specific synergies. Additionally, split-half validation revealed that minor specific modules such as M12 exhibited low spatial reliability, suggesting that low-prevalence modules should be interpreted with caution and verified through larger, longitudinal cohorts in future research. Future studies should systematically quantify crawling speed, stride characteristics, and more comprehensive anthropometric variables and incorporate these factors into appropriately specified multivariable or mixed-effects models in larger samples to better distinguish the contributions of task-related biomechanical factors from developmental changes in neuromotor control.

## Conclusion

This study utilized muscle synergy features extracted via concatenated non-negative matrix factorization (cNMF) to quantitatively reveal the central nervous system (CNS) strategies for motor dimensionality reduction and dynamic reorganization during hands-and-knees crawling in 4–6-year-old children.

Within individuals, muscle synergies during consecutive hands-and-knees crawling cycles in 4- to 6-year-old children exhibited relatively consistent spatial organization (Mean CS = 0.58), alongside considerable temporal variability (Circular SD = 16.73%), indicating relative rather than absolute spatial stability within the present pediatric sample. The total number of recruited muscle synergies (*N*) was significantly higher in the 6-year-old group than in the 4-year-old group. This pattern may reflect a progressive enhancement in the ability to “free” additional motor degrees of freedom as sensorimotor integration matures. These findings support Hypothesis 1.

The core shared synergies associated with basic support and propulsion (M4, M5, M6, M7, and M11) were predominantly dominated by large proximal muscle groups of the trunk and extremities. These modules maintained relatively consistent spatial organization and temporal phase consistency across both individuals and the five age groups (G1–G5), with no significant main effects of age detected on any spatiotemporal parameters. These findings are consistent with a relatively stable foundational coordination framework for hands-and-knees crawling that is maintained across the examined age range. They further suggest that proximal muscle coordination may provide a relatively stable basis for maintaining postural support and propulsion during crawling. These findings support Hypothesis 2.

The specific synergies associated with distal fine-tuning and compensatory adjustments (M1–M3, M8–M10, and M12) differed somewhat from the initial structural variability expectations outlined in the hypotheses. Although their internal spatiotemporal features did not undergo significant age-related shifts across the 4- to 6-year period, they exhibited substantial inter-individual temporal phase dispersion. Furthermore, their population prevalence exhibited substantial fluctuations and wide inter-individual variability during this interval. This pattern may reflect that, due to the lack of precise feedforward internal models specific to the current quadrupedal posture, the developing CNS may rely partly on online sensory feedback. It actively employs distal freeing of degrees of freedom together with dynamic trial-and-error processes to adapt to complex mechanical constraints. The combination of age-independent stability in internal spatiotemporal structures, high inter-individual temporal variability, and variable population recruitment across age groups partially supports Hypothesis 3.

## Supporting information

S1 Dataset Raw electromyography dataset. (ZIP)
