# Supplementary material for: Muscle synergy characteristics during hands-and-knees crawling in children aged 4–6 years
Source: PLoS One. 2026 Oct 8;21(10):e0358587. doi: 10.1371/journal.pone.0358587 (PMC13649141; doi:10.1371/journal.pone.0358587)
Supplement: Supplementary file 2 [file pone.0358587.s003.docx]

Response to Reviewers

Manuscript ID: PONE-D-26-23547

Manuscript Title: Muscle Synergy Characteristics during Hands-and-Knees Crawling in Children Aged 4–6 Years

Dear Dr. Mário Espada and Reviewers,

We sincerely appreciate the academic editor and reviewers for their constructive, detailed, and valuable comments, which have significantly helped us improve the quality of our manuscript. We have carefully addressed every single concern point-by-point, revised the manuscript thoroughly in accordance with all suggestions, re-analyzed partial statistical data, supplemented required references, clarified methodological ambiguities, softened overstated functional conclusions, and updated figures and formatting.

All revisions are clearly marked in the "Revised Manuscript with Track Changes" file, and the detailed point-by-point responses to each comment are listed below. We hope the revised version meets the publication requirements of PLOS ONE.

Best regards,

Corresponding Author: Dr. Yi Jia

-----------------------------------------------------------------------------------------------------------------

Part 1: Response to the Academic Editor

**Editor Comment:**

Congratulations on your work. Please revise the manuscript in light of the reviewers' major suggestions. Thank you.

**Response：**

We thank the academic editor for the positive evaluation of our research. We have comprehensively revised the whole manuscript focusing on all major methodological, statistical and interpretative concerns raised by two reviewers, and all revisions are recorded in the tracked-change manuscript file.

**Revision Location：**Entire manuscript with full revisions

-----------------------------------------------------------------------------------------------------------------

Part 2: Response to Reviewer 1

**Comment 1:**

A major weakness persists regarding the interpretation of the dimensionality reduction. The updated methodology resulted in the extraction of twelve group-level synergies from sixteen recorded muscles. Extracting twelve modules from sixteen channels represents a highly limited dimensionality reduction. The physiological validity of identifying almost as many synergies as recorded muscles requires cautious interpretation, as this outcome might reflect mathematical fractionation of the electromyographic data rather than authentic neural modularity.

Page 9, Line 306. The extraction of an average of 7.82 synergies per individual and 12 at the group level from a 16-muscle configuration is unusually high. Address whether the non-negative matrix factorization algorithm is fragmenting single muscle activations into independent modules, and discuss this specific risk within the context of the updated 90% threshold.

**Response：**

We sincerely thank the reviewer for this important comment. We agree that extracting an average of 7.82 synergies per individual (and 12 at the group level) from 16 recorded muscles requires careful interpretation, as the risk of mathematical over-decomposition cannot be completely excluded.

First, for the individual-level synergies, we added a discussion noting that the 90% VAF criterion is relatively strict and may require extra components to account for residual EMG variance. Furthermore, using concatenated NMF across ten continuous crawling cycles (without cycle averaging) preserves cycle-to-cycle variability. This method naturally requires more components to reconstruct the signal, which contributed to the higher individual synergy count.

Second, for the 12 group-level synergies, we clarified in the manuscript that these do not mean each child uses 12 modules at the same time. Instead, they were derived by clustering spatial weights across all 49 participants, representing a common set of recurring muscle-activation patterns shared across the group. Within this set, several highly common modules clearly contain multiple related muscles. Therefore, when a single muscle dominates a specific module, we now carefully interpret it as a local activation pattern for distal fine-tuning, rather than direct evidence of an independent neural command.

Finally, following your recommendation, we revised our interpretation of the age-related increase in synergy numbers in the Discussion. We removed statements about "adding functional modules" or "enhancing computational capacity." Instead, we now interpret this increase simply as reflecting greater differentiation and diversity of muscle-activation patterns during motor development.

**Revision Location：**

Discussion:

Revised the interpretation of the age-related increase in synergy numbers, removing absolute terms and focusing on the greater differentiation and diversity of muscle-activation patterns.

Added a dedicated paragraph discussing the risk of mathematical over-decomposition under the relatively strict 90% VAF threshold, and explained how preserving cycle-to-cycle variability via concatenated NMF contributes to the higher individual synergy count.

Clarified the nature of the 12 group-level synergies as a population-level repertoire, and added cautious interpretation regarding single-muscle-dominant modules as local activation patterns for distal fine-tuning.

**Comment 2:**

A minor weakness relates to the interpretation of motor variability. The authors correctly added a limitation regarding the inability to control for covariates such as crawling speed and anthropometrics using non-parametric statistical methods. The discussion attributes the high temporal phase dispersion to active sensorimotor exploration. The authors must acknowledge in the discussion that a portion of this temporal variability might directly stem from the uncontrolled variations in self-paced crawling speed across the distinct age groups. Page 12, Line 446. The limitations section identifies the lack of covariate control using traditional parametric tests. Add a brief note explaining how variations in self-paced crawling speed might directly inflate the reported temporal phase dispersion values observed in the specific synergies.

**Response：**

We sincerely thank the reviewer for this important clarification. We agree that the observed temporal phase dispersion should not be attributed solely to active motor exploration. Accordingly, we have revised the Discussion to acknowledge that part of the observed temporal phase dispersion may also arise from uncontrolled variations in self-paced crawling speed across children and age groups. We further note that differences in crawling speed may alter the relative timing of muscle activation within the crawling cycle and thereby contribute to the observed phase dispersion. The interpretation of active motor exploration has therefore been moderated to avoid attributing the observed temporal variability exclusively to central motor-control processes.

In addition, we have added a corresponding statement to the Limitations section, explicitly noting that variations in self-paced crawling speed may have contributed to the higher temporal phase dispersion observed in some specific synergies. A minor adjustment was also made to the Conclusion to ensure consistency with this more cautious interpretation.

**Revision Location：**

Discussion: The paragraph discussing the high temporal phase variability of the distal fine-tuning synergies (M1, M2, and M10) was revised to acknowledge that part of the observed temporal phase dispersion may be related to uncontrolled variations in self-paced crawling speed across children and age groups. The interpretation of active motor exploration was correspondingly moderated.

Limitations: The paragraph addressing uncontrolled covariates, including crawling speed, stride length, and anthropometric characteristics, was supplemented with a specific statement explaining that variations in self-paced crawling speed may contribute to the higher temporal phase dispersion observed for some synergies.

Conclusion: The corresponding statement regarding motor variability and motor exploration was slightly moderated to maintain consistency with the revised Discussion and Limitations.

**Comment 3 :**

Page 2, Line 41. The abstract states a mean N = 7.82 ± 1.05 synergies recruited. State the total number of muscles recorded directly in this sentence to provide immediate context for the dimensionality reduction ratio.

**Response：**

We sincerely thank the reviewer for this helpful suggestion. We agree that explicitly stating the number of recorded muscles alongside the mean synergy number provides clearer context for the degree of dimensionality reduction. We have therefore revised the corresponding sentence in the Abstract to explicitly state that 7.82 ± 1.05 synergies were extracted from the 16 recorded muscles.

**Revision Location：**

Abstract: The sentence reporting the mean number of recruited synergies was revised to:

“An average of 7.82 ± 1.05 muscle synergies were extracted from the 16 recorded muscles, with synergy number increasing significantly with age (p < 0.05).”

**Comment 4:**

Page 4, Line 106. The phrase "degrees of freedom redundancy (DOF redundancy)" contains unnecessary repetition. Revise this instance to "degree of freedom (DOF) redundancy" to improve readability.

**Response：**

We sincerely thank the reviewers for pointing out this formatting issue. We agree that the original expression “degrees of freedom redundancy (DOF redundancy)” was unnecessarily repetitive. We have revised it to “degree of freedom (DOF) redundancy” and ensured consistent terminology throughout the manuscript. The same correction was also applied to the corresponding occurrence in the Discussion to maintain consistent formatting.

**Revision Location：**

Introduction: “degrees of freedom redundancy (DOF redundancy)” was revised to “degree of freedom (DOF) redundancy.”

Discussion: The corresponding occurrence of “degrees of freedom redundancy (DOF redundancy)” was also revised to “degree of freedom (DOF) redundancy.”

**Comment5:**

Page 7, Line 235. The reported mean intra-individual spatial consistency is 0.58, and the temporal waveform similarity is 0.16. Clarify briefly in the results text that these values are low relative to adult steady-state locomotion, directing the reader to the developmental interpretation provided in the subsequent discussion section.

**Response：**

We sincerely thank the reviewer for this helpful suggestion. We agree that providing a brief adult locomotion reference would help readers better contextualize the relatively low intra-individual spatial and temporal similarity observed in the preschool cohort. We have therefore added a brief comparison in the Results section, noting that these values are relatively low compared with the high intra-individual synergy consistency reported during adult steady-state locomotion. We also direct readers to the subsequent Discussion for the developmental interpretation of this variability.

**Revision Location：**

Results: A brief comparative statement was added to contextualize the values:Compared with the high intra-individual consistency reported during natural-paced adult walking, these relatively low spatial and temporal similarity values indicate greater cycle-to-cycle variability in the present preschool cohort.” This addition directs readers to the subsequent Discussion for a comprehensive developmental interpretation of these findings.

-----------------------------------------------------------------------------------------------------------------

Part 3: Response to Reviewer 3

**Comment 1:**

Statistical Modeling and Covariates Observation: The authors indicated in their response that they were unable to apply Analysis of Covariance (ANCOVA) or Mixed-Effects Models due to the non-fulfillment of normality and homogeneity of variance assumptions for some variables, relying strictly on the Kruskal-Wallis test. Evaluation: While the authors' statistical rationale is understandable, the failure to control for variables such as "crawling speed" and "anthropometric characteristics" leaves a gap regarding whether the observed differences are purely age-related or stem from variations in individual motor performance. Required Modification: It would be beneficial to attempt data transformation (such as log transformation) to test parametric models or utilize Generalized Linear Mixed Models (GLMMs) that handle non-normal data better, while sufficiently clarifying this limitation in the limitations section.

**Response：**

We sincerely thank the reviewer for this constructive comment. We agree that individual differences in body size and task-related motor performance, particularly self-selected crawling speed, may influence the observed age-group differences.

We carefully considered the reviewer’s suggestions regarding data transformation and generalized linear mixed-effects modeling. However, the issue in the present study is not simply the non-normality of the data. First, our primary outcome measures have different distributional and measurement characteristics, including synergy number, N, similarity measures (CS and r), and temporal phase dispersion (Circular SD). Therefore, a single simple transformation, such as log transformation, would not provide a consistent and appropriate parametric framework for all outcome measures.

More importantly, the key task-related covariates highlighted by the reviewer, particularly crawling speed and stride characteristics, were not systematically quantified during data collection because participants crawled at a self-selected speed. Although body height and body mass were measured, they were not incorporated as covariates in the original analyses. Consequently, a post hoc transformed model or GLMM based only on the available variables would not adequately address the reviewer’s central concern regarding the potential confounding effect of individual motor performance, particularly crawling speed.

We therefore retained the original non-parametric statistical framework and have explicitly acknowledged this limitation in the revised manuscript. We have also moderated the interpretation of the observed age-group differences and clarified that the contribution of task-related motor performance and body size cannot be completely excluded.

**Revision Location：**

Materials and Methods – Data Acquisition: We clarified that the anthropometric measurements consisted of body height and body mass.

Discussion – Limitations: We explicitly acknowledged that body height and body mass were not included as covariates and that crawling speed, stride length, and other detailed kinematic variables were not systematically quantified. We further clarified that the observed age-group differences should not be interpreted as effects completely independent of individual body size or motor performance.

**Comment 2:**

Spatiotemporal Consistency Interpretation Observation: The average internal spatial consistency for individuals reached (CS = 0.58), which is relatively lower than the traditional consistency standards of (0.70 - 0.80) in mature automated movements. Evaluation: The authors successfully reframed this aspect in the discussion, considering this variability as a reflection of the "motor exploration" process in children rather than a measurement error. This interpretation must be emphasized with utmost care to prevent the muscle synergy from appearing so unstable that the study loses its reliability.

**Response：**

We sincerely thank the reviewer for this important clarification. We agree that the relatively lower spatial consistency (CS = 0.58) should not be interpreted as indicating unreliable or unstable muscle synergies. We have therefore clarified in the Discussion that the observed CS reflects greater cycle-to-cycle variability in the expression of the identified synergy patterns, rather than a lack of stability in the underlying muscle coordination. We have also maintained a cautious interpretation of this variability as potentially related to ongoing motor exploration.

**Revision Location：**

Discussion section**:** A clarification was added in the paragraph discussing the intra-individual spatial and temporal consistency results (the paragraph starting with "Furthermore, the data revealed...") to explicitly distinguish cycle-to-cycle variability from the reliability or stability of the underlying muscle coordination.

**Comment 3:**

Lack of Kinetic Validation Observation: The reviewers praised the authors' responsiveness regarding the "absence of ground reaction force (GRF) data" and the modification of terms to "hypothesized functions". Required Modification: It must be ensured that the tables (such as Table 2) and the text are completely free of any categorical phrasing indicating actual force generation, strictly adhering to probabilistic phrases such as ("it is hypothesized to contribute to...") as promised by the authors in their response.

**Response：**

We sincerely thank the reviewer for emphasizing this important point. We agree that, in the absence of direct GRF measurements, the biomechanical functions inferred from the identified muscle synergies should not be interpreted as confirmed mechanical functions. We would also like to clarify that the original Table 2 already framed the listed functions as hypothesized interpretations. To make this distinction even clearer and avoid any possible ambiguity, we have revised the table heading to “Hypothesized biomechanical functions.” In addition, we carefully re-checked the corresponding descriptions throughout the Results and Discussion and revised categorical wording that could imply confirmed force generation or mechanical contributions. These descriptions now consistently use more cautious expressions such as “hypothesized to contribute to,” “may contribute to,” and “potentially contributing to.” Thus, the functional interpretations are presented as hypothesis-based inferences rather than direct kinetic evidence.

**Revision Location：**

Table 2, Results, and Discussion：The heading of Table 2 was revised to “Hypothesized biomechanical functions” to make the hypothesis-based nature of the functional interpretation explicit. In addition, categorical functional statements in the Results and Discussion were revised to use more cautious, probabilistic wording where necessary.

-----------------------------------------------------------------------------------------------------------------

Part 3: Response to Reviewer 5

**Comment 1:**

Unresolved critical ethical timeline discrepancy (REQUIRES IMMEDIATE CLARIFICATION) In the revised Methods, the authors state that participants were recruited between November 2024 and May 2025, yet data collection is still reported as occurring between June 15 and November 20, 2024. The ethics approval number (SXULL2024082) implies review in August 2024. This creates an impossible chronology: data collection began in June 2024, approximately five months before the stated recruitment period and two months before the implied ethics approval date.

**Response：**

We sincerely thank the reviewer for identifying this apparent discrepancy in the ethical timeline. We carefully re-checked the original ethics documentation and study records and confirmed that the recruitment period reported in the revised manuscript as November 2024 to May 2025 was a typographical error. The actual participant recruitment and data collection period was June 15 to November 20, 2024, consistent with the study period specified in the ethics documentation.

We would also like to clarify that SXULL2024082 is the ethics approval/project identification number and does not represent a date. The original ethics document records the applicant’s signature on May 18, 2024, and the ethics committee approval/signature and official stamp on May 30, 2024, both of which preceded the study start date of June 15, 2024. Therefore, ethical approval was obtained before participant recruitment and data collection began.

The period from November 2024 to May 2025 referred to the subsequent data processing and analysis stage, rather than participant recruitment. To avoid further ambiguity, we have removed this analysis period from the description of participant recruitment and data collection and corrected the recruitment period in the revised manuscript to June 15 to November 20, 2024. The ethics documentation containing these dates was already submitted with the original manuscript as a supplementary document.

If the reviewer considers it helpful for clarifying the study timeline, we would be grateful for guidance as to whether the period of subsequent data processing and analysis should also be explicitly reported in the Methods section. We would be pleased to add this information if considered necessary. We sincerely apologize for the confusion caused by the typographical error.

**Revision Location：**

Materials and Methods – Subjects. The incorrect recruitment period “November 2024 to May 2025” has been corrected to “June 15 to November 20, 2024.” The relevant timeline statements have also been checked for consistency with the ethics documentation.

**Comment 2:**

Internal inconsistency in the 12-module taxonomy The new two-tier classification (shared vs. specific) applies dual criteria: prevalence >70% and coefficient of variation (CV) of prevalence across age groups <0.3. However, the classification appears inconsistently applied: • M4 (77.55% prevalence, Circular SD = 35.10%) is labeled "shared," whereas M8 (73.47% prevalence, Circular SD = 21.36%) is labeled "specific." M4 exhibits higher temporal variability and lower spatial consistency than M8, yet is classified as a stable core module. If the CV criterion explains this, the actual CV values for every module must be explicitly reported. • M6 (73.47% prevalence) is "shared," but its anatomical counterpart M8 (73.47% prevalence) is "specific." The manuscript does not justify why two modules with identical population coverage fall into different categories.

**Response：**

We sincerely thank the reviewer for this meticulous observation. The reviewer is absolutely correct that the Coefficient of Variation (CV) of prevalence across age groups is the precise discriminator here. We apologize for the oversight of not explicitly reporting the actual CV values for each module in the original manuscript, which understandably led to this confusion.

To clarify our rationale: our two-tier classification strictly relied on *two population-level criteria prior to assessing any spatiotemporal characteristics*: (1) overall prevalence > 70%, AND (2) robust developmental stability across age groups, defined as CV < 0.3.

Regarding the classification of M6 versus M8, although both modules shared an identical overall prevalence of 73.47%, their developmental trajectories differed significantly. M6 maintained a stable recruitment rate across the five age groups (CV = 0.18), fully satisfying the stability criterion (CV < 0.3), and was thus classified as a "shared" synergy. In contrast, the prevalence of M8 fluctuated substantially across age groups (CV = 0.33). Because M8 failed to satisfy the developmental stability criterion (CV ≥ 0.3), it was strictly classified as a "specific" synergy.

Furthermore, regarding M4 versus M8, the reviewer perceptively noted that M4 exhibits higher temporal variability (Circular SD) than M8. We would like to clarify that spatiotemporal characteristics (Circular SD and Spatial Consistency) were the *outcomes* evaluated *after* the classification was established; they were not the a priori criteria used to classify the modules. M4 was classified as a shared synergy because it met the dual population-level criteria: high overall prevalence (77.55%) and stable age-group distribution (CV = 0.25, which is < 0.3).

We fully agree that providing the actual CV values is crucial for methodological transparency. Per the reviewer's excellent suggestion, we have now added a "CV of Prevalence" column to Table 2, reporting the actual values for all 12 modules. Furthermore, we have expanded the Results section to explicitly justify the classifications of the modules by integrating their respective CV values into the text.

**Revision Location：**

Results – Table 2: A new column titled "CV of Prevalence" has been added to report the exact CV values for all 12 modules.

Results – Inter-individual clustering and spatiotemporal characteristics analysis of muscle synergies: We have updated the text to explicitly report the CV values alongside the prevalence data, thereby clarifying that M4 and M6 met the criteria for shared synergies (CV < 0.3), whereas M8 and other specific synergies did not (CV ≥ 0.3 or overall prevalence < 70%).

**Comment 3:**

Furthermore, several "specific" modules show extremely low population coverage: M2 (30.61%), M9 (36.73%), and M12 (26.53%). With only 49 participants, a module detected in ~8–15 individuals risks being an algorithmic artifact of over-segmentation rather than a genuine physiological synergy. The authors should provide robustness validation (e.g., split-half reliability, bootstrap resampling) to prove these low-prevalence modules are reproducible. **Response：**

We deeply appreciate this critical and constructive feedback. We completely agree that modules with low population coverage require rigorous robustness validation to rule out the possibility of algorithmic over-segmentation.

To address this concern, we performed a repeated split-half validation across our cohort. Specifically, the participant pool was randomly split into independent halves across multiple iterations, and muscle synergy extraction and cluster matching were re-evaluated. The spatial consistency (Cosine Similarity, CS) between the split-half subsets and the full-cohort reference templates was quantified to rigorously assess reproducibility.

The results of this validation have now been integrated into the revised manuscript (both in the Results section and Table 2), revealing a clear and informative dichotomy among the low-prevalence modules highlighted by the reviewer:

1. Robustness of M2 and M9:

Module 9 (M9 — Prevalence: 36.73%): Despite its lower population coverage, M9 demonstrated outstanding spatial stability under split-half validation, yielding a high mean cosine similarity of $0.95\pm0.09$ (Median: 0.966; 95% CI: [0.806, 0.986]). This confirms that when M9 is present, its spatial structure is highly reproducible, proving it to be a genuine, albeit selectively recruited, neuromuscular control pattern rather than an algorithmic artifact.

Module 2 (M2 — Prevalence: 30.61%): Module 2 yielded a moderate split-half reliability with a mean cosine similarity of 0.77±0.22 (Median: 0.895; 95% CI: [0.358, 0.970]). While showing higher variance due to inter-individual differences in distal adjustments among the children who express it, its spatial template remains identifiable and supports the active "distal freeing" strategy characteristic of this developmental stage.

2. Caution regarding M12:

Module 12 (M12 — Prevalence: 26.53%): Module 12 exhibited a lower mean split-half cosine similarity of 0.45±0.29 (Median: 0.288; 95% CI: [0.154, 0.958]). Combined with its low population coverage, this indicates that M12 is sensitive to sample partitioning and carries a higher risk of being an unstable or sample-dependent peripheral component. We have explicitly acknowledged this limitation in the revised text and emphasized that M12 should be interpreted with caution.

**Revision Location：**

Results Section: Added a paragraph detailing the methodology and results of the repeated split-half validation for all 12 modules within the "Inter-individual clustering and spatiotemporal characteristics analysis of muscle synergies" subsection.

Table 2: Added a new column for Split-half CS (Mean ± SD) to provide quantitative robustness evidence for each synergy module.

Discussion Section: Expanded the text near the analysis of specific synergies to note that M12 (dominated by the right rectus femoris) exhibited notably lower split-half stability (mean CS = 0.451), indicating that it should be interpreted with caution as an unstable or sample-sensitive peripheral component.

Limitations Section: Added a cautious note regarding the low spatial reliability of minor specific modules like M12, recommending future verification through larger, longitudinal cohorts.

**Comment 4:**

The "prevalence" metric is undefined and mathematically inconsistent In the Results, the authors report developmental trajectories using prevalence values such as: • M2 in G1: 1.30% • M2 in G4: 10.94% • M9 in G4: 0% • M9 in G5: 3.41% If "prevalence" means the percentage of participants in a group exhibiting the module, these decimals are impossible. With n=10 in G1, the smallest non-zero prevalence is 10% (1/10). With n=9 in G4, the smallest non-zero prevalence is 11.11% (1/9). Values like 1.30% or 10.94% cannot represent integer participant counts. The authors must clarify the exact formula used to calculate these percentages. If they represent something other than binary occurrence (e.g., mean W weight, VAF contribution), the terminology must be changed, as "prevalence" and "detection rate" imply a categorical present/absent variable.

**Response：**

We deeply appreciate the reviewer’s exceptionally careful reading and mathematical scrutiny. The reviewer is absolutely correct. We sincerely apologize for this terminological inconsistency, which understandably led to confusion.

To clarify, the decimal percentages reported in that specific paragraph (e.g., 1.30%, 10.94%) did *not* represent the true population prevalence. Instead, they represented the Relative Module Frequency—calculated as the total number of vectors assigned to a specific module divided by the total number of muscle synergies extracted across all participants within that specific age group (e.g., in G4, M2 accounted for 7 out of the 64 total synergy modules extracted in that group, yielding 7/64 = 10.94%).

However, we fully agree with the reviewer that labeling this metric as "prevalence" was terminologically incorrect and mathematically inconsistent with the true binary "population prevalence" (Detection Rate) used elsewhere in the manuscript (e.g., the threshold of >70% prevalence and the CV calculations). The true population prevalence is exactly as the reviewer described: a categorical present/absent variable divided by the group size (e.g., $n=10$ or $n=9$).

To completely resolve this inconsistency and ensure mathematical rigor throughout the paper, we have removed the confusing "Relative Module Frequency" decimal percentages. We have updated the developmental trajectories in both the Results and Discussion sections to report exclusively the true Population Prevalence (Detection Rate).

For example, the trajectory of M2 is now accurately reported using its true population prevalence: starting at 10% in G1 (1 out of 10 participants), peaking at 66.7% in G4 (6 out of 9 participants), and declining to 40% in G5 (4 out of 10 participants).

**Revision Location：**

Results: We updated the paragraph describing the developmental trajectories of specific synergies (M1, M2, M9, M10), replacing the erroneous decimal frequencies with the correct categorical prevalence rates (e.g., 10%, 50%, 66.67%). We also clarified the terminology by explicitly referring to this as "population prevalence (detection rate)."

Discussion: We corrected the corresponding prevalence data points mentioned in the Discussion (e.g., changing the M2 peak in G4 from 10.94% to 66.67%).

**Comment 5:**

Over-interpretation of "nonlinear reorganization" from low-prevalence fluctuations The manuscript frames fluctuations in specific modules (e.g., M2 peaking at 10.94% in G4; M9 dropping to 0% in G4 and recovering to 3.41% in G5) as evidence of "pronounced nonlinear developmental reorganization" and a "critical window" around 5.5 years. Given the small group sizes (n=9–10), a module dropping from ~1 subject to 0 subjects, or rising to ~1 subject, is indistinguishable from sampling noise. A single participant's data should not drive a developmental theory. The authors must: • Report exact participant counts (integers) alongside percentages; • Perform permutation tests or bootstrap confidence intervals for prevalence differences across groups; • Temper the "critical window" language unless supported by more robust statistics than a single-group fluctuation.

**Response：**

We sincerely thank the reviewer for this crucial methodological critique. You are absolutely correct that fluctuations in small cohorts ($n=9-10$) are heavily influenced by sampling noise, and that our previous framing of a "critical window" and "nonlinear reorganization" was a significant over-interpretation of the data. We have strictly followed your recommendations to ensure our statistical reporting and conclusions are robust and accurately reflect the sample size.

Here is how we have addressed each of your specific requirements:Reporting Exact Participant Counts: We have updated Table 2 and all developmental trajectory descriptions in the Results section to explicitly report the exact participant counts (integers) alongside the population prevalence percentages.

Bootstrap Confidence Intervals: As requested, we performed non-parametric bootstrap resampling (10,000 iterations) to calculate the 95% Confidence Intervals (CIs) for all population prevalence estimates. These CIs are now formally reported in Table 2 and throughout the text. As you rightly anticipated, the resulting wide confidence intervals (e.g., M2 in G4: [33.3%, 100.0%]) confirm the substantial variance and sampling noise inherent in our subgroup sizes.

Tempering the 'Critical Window' Language: Guided by these CI results, we have completely removed the deterministic "critical window" and "pronounced nonlinear reorganization" phrasing from the Abstract, Introduction (Hypotheses), Discussion, and Conclusion. Instead of claiming a fixed developmental shift, we now accurately interpret these wide fluctuations as evidence of high inter-individual variability. We have reframed the discussion to suggest that this variability reflects an active, trial-and-error "sensorimotor exploration" strategy used by the developing CNS to manage redundant degrees of freedom.

We believe these revisions align our conclusions strictly with the statistical reality of the data, and we are highly grateful for your guidance in strengthening the scientific rigor of our manuscript.

**Revision Location：**

Abstract: Revised the final sentence to remove "nonlinear reorganization" and emphasize inter-individual variability.

Introduction: Updated Hypothesis 3 to expect "highly variable and fluctuating recruitment patterns" rather than "nonlinear developmental reorganization."

Results (Table 2): Added exact participant counts and Bootstrap 95% CIs to the prevalence column.

Results (Age-related differences section): Included exact counts and 95% CIs when reporting module trajectories (e.g., M1, M2, M9, M10).

Discussion: Revised the paragraphs discussing G4/G5 transitions to interpret fluctuations as active sensorimotor exploration rather than a deterministic critical window.

Conclusion: Replaced "nonlinear developmental reorganization" with "substantial fluctuations and wide inter-individual variability."

**Comment 6:**

Statistical significance versus practical significance The authors report that the total number of synergies (N) was significantly higher in G5 than G1 (adjusted p = 0.035). However, the mean N increased from approximately 7 to 8 over a 24-month span. The manuscript does not report the effect size (e.g., Cohen's d or rank-biserial correlation) for this pairwise comparison. A difference of ~1 synergy, while statistically significant, may lack developmental or clinical meaningfulness. Additionally, the authors continue to rely on Kruskal-Wallis tests without covariate adjustment. While they now transparently acknowledge this as a limitation, the inability to disentangle biomechanical constraints from neurodevelopmental effects remains a major interpretive weakness that cannot be fully resolved by disclaimer alone.

**Response：**

We thank the reviewer for raising this thoughtful question regarding statistical versus practical significance.

To address this:Effect Size Reporting for Total Synergies ($N$): We have now calculated and incorporated the non-parametric effect size for the pairwise comparison between the 4-year-old group (G1, mean $N=7.70$) and the 6-year-old group (G5, mean $N=8.80$). The rank-biserial correlation yielded $r_{rb}=0.64$, indicating a robust, medium-to-large effect size that confirms the practical magnitude of this difference beyond a mere artifact of a low $p$-value.

Effect Size Reporting for Module M8: Additionally, to provide complete transparency on our significant modular findings, we have added the effect size for the temporal phase dispersion difference of Module M8 between G1 and G4 (adjusted $p=0.020$), which yielded $r_{rb}=0.96$.

Developmental and Functional Meaningfulness: While an increase of ~1 synergy module over a 24-month span appears numerically modest, it holds profound significance in the framework of motor modularity and neurodevelopment. During the preschool years (ages 4 to 6), improvements in sensorimotor integration allow the CNS to transition from conservative, rigid co-activation schemas toward a more differentiated modular repertoire. This incremental expansion reflects the progressive "freeing" of redundant degrees of freedom and a qualitative maturation of multi-joint coordination strategies, which is both biomechanically and developmentally meaningful.

**Revision Location：**

Results (Total Synergies): Updated the sentence to include the effect size: "...revealed that the 6-year-old group (G5) exhibited a significantly greater number of muscle synergies than the 4-year-old group (G1) (adjusted $p=0.035$, rank-biserial correlation $r_{rb}=0.64$)."

Results (Module M8): Updated the sentence to include the effect size: "...exclusively between G1 and G4 (adjusted $p=0.020$, rank-biserial correlation $r_{rb}=0.96$)..."

Discussion: Added a dedicated explanatory paragraph at the end of the first paragraph in the Discussion (following the comparison with infant crawling studies): "Although the mean number of recruited muscle synergies increased modestly from approximately 7 to 8 (specifically, from $7.70\pm0.82$ in G1 to $8.80\pm0.79$ in G5) over the 24-month span, this statistical difference demonstrated a robust, medium-to-large effect size ($r_{rb}=0.64$). From a neuromotor control perspective, this incremental expansion is far from trivial. In the context of motor modularity, adding a functional module during preschool development reflects a qualitative enhancement in the central nervous system’s computational capacity. It mirrors the progressive transition from rigid, whole-body co-activation patterns toward a more differentiated and flexible modular repertoire—successfully 'freeing' redundant degrees of freedom as sensorimotor integration matures."

**Comment 7:**

Response to reproducibility concerns (Reviewer 2) The authors reframed the low intra-individual spatial consistency (CS = 0.58) and very low temporal waveform similarity (r = 0.16) as "authentic reflection of immature neuromuscular control." While this developmental perspective is plausible, the manuscript still describes these modules as maintaining "relatively stable spatial structures" and "robust spatial consistency." There is an unresolved tension between the quantitative values (which fall below accepted adult thresholds for reproducibility) and the qualitative descriptors. I suggest the authors either report normative pediatric thresholds or explicitly state that 0.58 represents relative (not absolute) stability in this population. **Response：**

We sincerely thank the reviewer for this important clarification. We agree that the previous descriptions of “relatively stable spatial structures” and “robust spatial consistency” could overstate the degree of reproducibility indicated by the observed CS value (0.58). We have therefore revised the relevant Discussion and Conclusion to explicitly clarify that CS = 0.58 represents relative rather than absolute spatial stability within the present pediatric sample. We also revised the corresponding qualitative descriptions to avoid implying high spatial reproducibility and further distinguished the relatively consistent spatial organization from the greater temporal variability reflected by the low waveform similarity (r = 0.16). These revisions ensure that the qualitative interpretation is consistent with the quantitative findings.

**Revision Location：**

Discussion and Conclusion.：The relevant descriptions of spatial consistency were revised to avoid overstating reproducibility. The Discussion now explicitly states that CS = 0.58 should be interpreted as relative rather than absolute spatial stability within the present pediatric sample, and the distinction between spatial consistency and temporal variability was further clarified.

**Comment 8:**

Citation balance The response to multiple unrelated comments (signal processing, algorithmic standardization, distal motor control, developmental neuromechanics) all cite the same author (Dhahbi W, 2025–2026). While these may be valid references, such concentrated citation of a single research group across disparate topics raises questions about citation balance and whether these specific papers represent the most established sources for each individual point.

**Response：**

We sincerely thank the reviewer for raising this important concern regarding citation balance. We agree that different methodological and theoretical statements should be supported by literature that is directly relevant to each specific topic rather than relying predominantly on publications from a single research group.

In response, we carefully re-examined the relevant citations and selectively supplemented or replaced several references with established and independent sources, while retaining Dhahbi et al. references where they remain directly relevant to the corresponding statements. Specifically, Ting et al. (2015) was additionally cited in the Introduction to provide an established theoretical reference for muscle synergies; Winter (2009) was used in place of the previous reference for cycle segmentation; Besomi et al. (2020) was used for the data-preprocessing procedure; and Merletti and Cerone (2020) was additionally cited to provide an established methodological reference for EMG processing and analysis.

These changes were made selectively rather than by systematically removing the Dhahbi et al. references. Our aim was to improve citation balance while ensuring that each methodological or theoretical statement is supported by the most appropriate and representative literature.

**Revision Location：**

Introduction, paragraph 3: Ting et al. (2015) was added as an independent theoretical reference.

Materials and Methods – Segmentation of Cycles, paragraph 1: the previous reference was replaced with Winter (2009).

Materials and Methods – Data Preprocessing, paragraph 1: the previous reference was replaced with Besomi et al. (2020).

Materials and Methods – Muscle Synergy Extraction and Non-Negative Matrix Factorization, paragraph 1: Merletti and Cerone (2020) was added as an independent methodological reference.

-----------------------------------------------------------------------------------------------------------------

Part 4: Response to Reviewer 6

**Comment 1:**

Terminology Alignment in Text vs. Tables: 1.1 In Table 2 and the text, ensure that muscle abbreviations are entirely consistent throughout (e.g., R_TB vs. R-TRI, L_GAS vs. L-GAS). The authors updated several in-text mentions, but a quick consistency audit across Table 2 and Fig 3 labels will ensure seamless readability.

**Response：**

We sincerely thank the reviewer for pointing out this terminology consistency issue. We have conducted a comprehensive consistency check of muscle abbreviations throughout the manuscript, with particular attention to Table 2 and the labels in Fig. 3. All muscle abbreviations have now been standardized to ensure consistent naming and formatting across the text, tables, and figures.

**Revision Location：**

Table 2, Fig. 3, and the corresponding descriptions throughout the manuscript. Muscle abbreviations were systematically checked and standardized for consistency.

**Comment 2:**

Degrees of Freedom Formatting: 2.1 Ensure standard formatting for terms like "degrees of freedom" (DOF) without typographical duplication in parentheses (e.g., "degrees of freedom (DOF)")

**Response：**

We sincerely thank the reviewers for pointing out this formatting issue. We agree that the original expression “degrees of freedom redundancy (DOF redundancy)” was unnecessarily repetitive. We have revised it to “degree of freedom (DOF) redundancy” and ensured consistent terminology throughout the manuscript. The same correction was also applied to the corresponding occurrence in the Discussion to maintain consistent formatting.

**Revision Location：**

Introduction: “degrees of freedom redundancy (DOF redundancy)” was revised to “degree of freedom (DOF) redundancy.”

Discussion: The corresponding occurrence of “degrees of freedom redundancy (DOF redundancy)” was also revised to “degree of freedom (DOF) redundancy.”

**Comment 3:**

Figure High-Resolution Check: 3.1 Confirm that all submitted figure files (Fig1.tif through Fig5.tif) render with crisp text overlay and clean axis labels in the final typesetting build.

**Response：**

We sincerely thank the reviewer for this helpful reminder. We have carefully checked all submitted figure files (Fig. 1–Fig. 5) and confirmed that the original TIFF files provide clear and legible text, axis labels, tick marks, legends, and other graphical elements at the submitted resolution. No apparent pixelation, distortion, or loss of readability was observed. The figure files have therefore been carefully reviewed and prepared to ensure their suitability for the final typesetting process

**Revision Location：**

Fig. 1–Fig. 5. All figure files were reviewed and, where necessary, re-exported as high-resolution TIFF files to ensure clear text, axis labels, legends, and graphical elements in the final submission.
