# Supplementary material for: Muscle synergy characteristics during hands-and-knees crawling in children aged 4–6 years
Source: PLoS One. 2026 Oct 8;21(10):e0358587. doi: 10.1371/journal.pone.0358587 (PMC13649141; doi:10.1371/journal.pone.0358587)
Supplement: Supplementary file 4 [file pone.0358587.s002.docx]

Response to Reviewers

Manuscript ID: PONE-D-26-23547

Manuscript Title: Muscle Synergy Characteristics during Hands-and-Knees Crawling in Children Aged 4–6 Years

-----------------------------------------------------------------------------------------------------------------

Dear Dr. Mário Espada, Reviewer 1 and Reviewer 2,

We sincerely appreciate the editorial team and two anonymous reviewers for the constructive, detailed and valuable comments to improve the quality of our manuscript. We have carefully addressed every single concern point-by-point, revised the manuscript thoroughly in accordance with all suggestions, re-analyzed partial statistical data, supplemented required references, clarified methodological ambiguities, softened overstated functional conclusions, and updated figures and formatting.

All revisions are clearly marked in the "Revised Manuscript with Track Changes" file, and the detailed point-by-point responses to each comment are listed below. We hope the revised version meets the publication requirements of PLOS ONE.

Best regards,

Corresponding Author: Dr. Yi Jia

-----------------------------------------------------------------------------------------------------------------

Part 1: Response to the Academic Editor

Editor Comment:

Congratulations on your work. Please revise the manuscript in light of the reviewers' major suggestions. Thank you.

Response：

We thank the academic editor for the positive evaluation of our research. We have comprehensively revised the whole manuscript focusing on all major methodological, statistical and interpretative concerns raised by two reviewers, and all revisions are recorded in the tracked-change manuscript file.

Revision Location：Entire manuscript with full revisions

-----------------------------------------------------------------------------------------------------------------

Part 2: Response to Reviewer 1

**Comment 1 (Major Weakness 1: Lack of Kinetic Validation)**

**Comment:**

The study relies exclusively on surface electromyography (sEMG) and kinematic trajectories to map muscle synergies during hands-and-knees crawling. Making definitive functional claims about "anti-gravity support" and "forward propulsion" without synchronized kinetic data (such as ground reaction forces from force platforms) renders the biomechanical interpretations speculative.

**Response：**

We sincerely thank the reviewer for this rigorous and insightful evaluation. We fully agree that assigning absolute kinetic functions (e.g., "anti-gravity support" or "forward propulsion") based solely on surface electromyography (sEMG) and kinematic data lacks direct mechanical validation. To ensure our conclusions strictly align with the available evidence, we have comprehensively softened these statements throughout the revised manuscript.

1.Softened inferential tone: In the Abstract and Discussion sections, we explicitly reframed these biomechanical roles as inferences. We introduced speculative phrases such as "hypothesized to contribute to," "postulated to perform," and "likely associated with." This is intended to clearly convey to the reader that these functions are inferred based on spatial anatomical weights rather than being measured kinetic outputs.

2.Modified table titles: We have rigorously updated the title of Table 2 to refer to "hypothesized biomechanical functions."

3.Added limitation statement: In the Limitations section of the Discussion, we now explicitly acknowledge the lack of synchronous GRF data as a major limitation of the present study. We specifically added the following statement: "Second, the investigation of neural control strategies relied primarily on surface electromyography (sEMG) signals without synchronous kinetic validation parameters. Consequently, mechanical contributions cannot be directly quantified."

**Revision Location：**

1.Abstract & Discussion: Comprehensively updated the speculative statements regarding biomechanical functions.

2.Results: The title of Table 2 has been modified.

3.Discussion (Limitations): Added a specific statement regarding the lack of kinetic validation parameters.

**Comment 2 (Major Weakness 2: High Attrition Rate and Sub-sample Validity)**

**Comment:**

The exclusion of 7 out of 50 participants significantly impacts the sub-group distributions. Specifically, the 40% exclusion rate in the G5 (72–77 months) group drastically lowers the statistical power of the developmental claims made regarding the "re-emergence" of the M6 synergy.

**Response：**

We sincerely thank the reviewer for astutely pointing out this critical statistical issue. We fully agree that the excessively high data attrition rate in the original manuscript (especially the high exclusion rate in the original G5 group) severely compromised the representativeness of the subsample, resulting in a lack of sufficient statistical power for our previous conclusions regarding the developmental trajectories of synergy modules.

To fundamentally resolve this issue and adequately address your concerns, we have taken the following key actions in this revision:

1.Optimizing data processing and raising the VAF threshold: We rigorously reviewed and optimized the preprocessing of the electromyographic (EMG) signals and algorithm parameters. Furthermore, the extraction threshold for the variance accounted for (VAF) was strictly increased from 85% to 90%, a more rigorous standard in motor dynamics research.

2.Exceptionally high data retention rate: Thanks to the optimized processing pipeline, out of the 50 initially recruited participants, only one participant in the 66–71 months age group was excluded due to electromyographic (EMG) artifacts. The final valid sample size included in the subsequent statistical analyses reached 49 participants (yielding an overall data retention rate of 98%). For the G5 (72–77 months) group, which you specifically expressed concern about, the data retention rate is now 100% (10/10).

3.Re-evaluating conclusions on developmental trajectories: Leveraging the complete and well-balanced new subsample data, we re-clustered and re-analyzed all results, yielding twelve group-level muscle synergies. Based on the current robust statistical power, we found that specific synergies (e.g., M2, M9, M10) did not simply exhibit an absolute "re-emergence" as stated in the original manuscript. Instead, their population prevalence underwent pronounced nonlinear developmental reorganization. We have thoroughly rewritten the relevant paragraphs in the Discussion and Conclusions sections of the revised manuscript to ensure that all conclusions are built upon a solid data foundation.

This major data re-analysis has greatly enhanced the reliability of our study, and we are deeply grateful for your constructive criticism.

**Revision Location：**

1.Abstract: Explicitly updated the final sample size included in the analysis to 49 healthy preschool children.

2.Materials and Methods (Subjects & Muscle Synergy Extraction): Detailed the data attrition showing only one participant was excluded , and added the methodological setting of increasing the optimal number of muscle synergies threshold to VAF ≥ 90%.

3.Results: Comprehensively updated the data based on the final 49 valid participants, completely removing the negative statements regarding the high exclusion rate in the G5 group from the original manuscript.

4.Discussion and Conclusions: In light of the latest data, replaced the previous "re-emergence" hypothesis, which resulted from limited sample size, with the more rigorous theory of nonlinear dynamic reorganization trajectories.

**Comment 3 (Major Weakness 3: Statistical Modeling Deficiencies)**

**Comment:**

The statistical approach relies on basic non-parametric tests (Kruskal-Wallis) to assess age group differences. This method fails to control for critical confounding covariates such as crawling speed, stride length, and individual anthropometrics, which fundamentally dictate muscle synergy temporal activation and spatial composition.

**Response：**

We sincerely thank the reviewer for this highly constructive and pertinent statistical feedback. We fully agree that key covariates, including crawling speed, stride length, and individual anthropometric characteristics, are critical confounding factors that ideally should be strictly controlled using analysis of covariance (ANCOVA) or mixed-effects models.

Upon receiving your insightful comments, we immediately attempted to incorporate these covariates into our statistical models. However, during assumption testing, we found that due to the inherently high variability of children's synergy data, several spatiotemporal parameters extracted in this study (e.g., Circular SD and Pearson's r) failed both the Shapiro-Wilk test for normality and Levene's test for homogeneity of variances. Under the circumstances of violating the core assumptions of parametric tests, forcefully applying a traditional ANCOVA could lead to severe bias in statistical inference (e.g., false positives or false negatives).

Driven by our commitment to data authenticity and statistical rigor, we had to retain the non-parametric Kruskal-Wallis test—which does not impose strict distributional assumptions—as the primary analytical method for the current study, and were unable to forcefully incorporate the covariates. To address your valid concerns and ensure the scientific objectivity of our manuscript, we have added a dedicated paragraph to the Limitations section. We not only explicitly acknowledge that failing to systematically control for these covariates is a major limitation, but we also explicitly state: "The existing statistical approach, which relies on conventional non-parametric tests (e.g., the Kruskal-Wallis test), cannot fully disentangle 'peripheral biomechanical constraints' from 'central neurodevelopmental effects' ." Furthermore, we propose that "future research with expanded sample sizes should incorporate Generalized Linear Mixed Models (GLMMs) or non-parametric analysis of covariance (ANCOVA) frameworks to precisely adjust for these confounding factors."

We hope the reviewer understands this statistical compromise necessitated by the actual data distribution, and appreciates our candid and profound reflection on this matter within the Limitations section.

**Revision Location：**

1.Discussion (Limitations): Added a new paragraph detailing the limitation of uncontrolled key covariates, and outlining future solutions using GLMMs and non-parametric ANCOVA frameworks.

**Comment 4 (Major Weakness 4: Permissive VAF Threshold)**

**Comment:**

The variance accounted for (VAF) threshold of 85% is comparatively low for concatenated non-negative matrix factorization (cNMF) in locomotion studies, which typically employ a 90% or higher threshold. This permissive cutoff risks overfitting and extracting non-physiological movement noise as valid "sporadic" synergies.

**Response：**

We sincerely thank the reviewer for this highly constructive and professional feedback. We fully agree with your assessment that a VAF threshold of 85% is relatively lax in the context of motor synergy research, which carries the risk of overfitting and capturing non-physiological noise.

To ensure the physiological validity of the extracted synergy modules and the rigor of our data reconstruction, we have fundamentally adjusted our extraction criteria in the revised manuscript:

1.Raised the VAF threshold: We re-ran the entire algorithmic pipeline, strictly increasing the global VAF threshold for determining the optimal number of synergies (N) from 85% to 90%. This adjustment allows our model to strike a better balance between reconstruction accuracy and model complexity, effectively filtering out mathematical noise.

2.Supplemented methodological literature support: To confirm the applicability and authority of this threshold in multi-limb movement studies (particularly hands-and-knees crawling), we have supplemented the Methods section with key literature support (Li C, Chen X, Zhang X, et al., Frontiers in Neuroscience, 2023), which explicitly identifies 90% as a widely accepted rigorous cutoff value in this field.

3.Comprehensive update of Results and Limitations: After applying the 90% threshold, the mean number of extracted modules was updated to 7.82 ± 1.05. This more refined modular partitioning accurately reflects the complexity of children's multi-degree-of-freedom control strategies. Furthermore, as this methodological shortcoming has been overcome, we have removed the previous statement regarding the lower VAF threshold from the "Limitations" section of the Discussion.

We are confident that this critical modification has significantly enhanced the reliability and scientific value of our study's conclusions.

**Revision Location：**

1.Methods (Determination of the number of synergy modules [N] and data quality control): Explicitly stated that the VAF threshold has been raised to 90% and added the citation of Li C, et al. (2023) .

2.Results (Number of muscle synergies and Intra-individual spatiotemporal control Characteristics): Comprehensively updated the statistics for the number of extracted synergies and their distribution based on the VAF ≥ 90% criterion.

3.Discussion & Limitations: Removed the discussion concerning the lower 85% threshold.

**Minor Weakness 1 (Arbitrary Muscle Dominance Threshold)**

**Comment:**

The use of an arbitrary threshold (W > 0.5) to define dominant muscles within a module lacks a rigorous mathematical foundation.

**Response：**

We sincerely thank the reviewer for this rigorous methodological review. We fully agree that the criterion of W > 0.5 for defining dominant muscles in our original manuscript was arbitrary and lacked sufficient literature or mathematical support. To rectify this deficiency and ensure methodological rigor, we have replaced this arbitrary threshold in the revised manuscript with a standard supported by established literature in this specific field of research. Specifically, we have adjusted the normalized weight threshold for defining dominant (active) muscles to W > 0.3. This criterion is directly adapted from highly relevant, peer-reviewed literature (Xiong QL, Wu XY, Yao J, et al., Frontiers in Neurology, 2018). That study utilized synchronized sEMG and kinematic analysis to investigate inter-limb muscle synergies in children during hands-and-knees crawling and explicitly stated: "Specifically, muscles in a synergy were defined as active if their normalized weight values exceeded 0.3". Adopting this threshold, which has been strictly validated within the field, not only provides a solid methodological foundation for our muscle function classification but also enhances the comparability of our study with other research on crawling motor control. Based on this updated W > 0.3 criterion, we have comprehensively updated the description in the Methods section, the threshold reference line in Fig 3, and the list of dominant muscles for each synergy module in Table 2.

**Revision Location：**

1.Methods (Cross-individual module clustering and quantification of spatiotemporal characteristics): Explicitly stated that the threshold has been updated to 0.3 and supplemented with the key reference (Xiong et al., 2018).

2.Results (Fig 3 caption & Table 2): The reference line for Fig 3 has been updated toW=0.3; the title of Table 2 and the dominant muscles listed within have been comprehensively updated according to the new threshold.

**Minor Weakness 2 (Data Interpolation Risks)**

**Comment:**

The uniform time-normalization to 100 points via PCHIP interpolation removes temporal variations in stance/swing phase ratios across the different age groups, potentially masking crucial developmental differences in motor control timing.

**Response：**

We sincerely thank the reviewer for their keen insight and professional guidance regarding the details of our data processing. We fully agree with your perspective that uniformly time-normalizing each complete crawling cycle to 100 data points eliminates the absolute time dimension and may potentially "obscure potential developmental differences" in the stance-to-swing phase ratios across age groups.

Because the primary objective of this study was to explore the spatial structures and reorganization patterns of "global muscle synergy patterns," we did not explicitly partition the crawling cycles into distinct stance and swing phases for separate normalization during the initial study design. However, the issue you raised is critical; this global normalization strategy indeed constitutes a methodological compromise that limits a deeper investigation into fine-grained temporal control characteristics.

To ensure the objectivity and rigor of the manuscript, we have fully adopted your suggestion by adding a dedicated paragraph in the "Limitations" section (the third limitation) of the revised manuscript. In this section, we explicitly acknowledge that the current global time-normalization approach may "obscure potential developmental differences in the stance-to-swing temporal ratio," and we formally propose that future research should combine comprehensive spatiotemporal kinematic data with "phase-specific" normalization strategies to more precisely elucidate these temporal characteristics.

**Revision Location：**

1.Discussion (Limitations): Added a third limitation paragraph, which provides a comprehensive and candid discussion regarding the potential risks associated with global time interpolation and outlines directions for future improvement.

**Specific Comment 1 (Page 2, Line 46)**

**Comment:**

The claim that the distal fine-tuning synergy module (M6) was absent (0.0% detection rate) in the 66–71-month age group but re-emerged in the 72–77-month group relies on extremely small and unequal sub-samples after data exclusion. Address the high probability that this is a statistical artifact rather than a genuine physiological phenomenon.

**Response：**

We sincerely thank the reviewer for their exceptionally rigorous scientific attitude. We fully agree with your assessment: in our original manuscript, due to the high data exclusion rate (up to 40% in the original G5 group), the conclusion regarding the "re-emergence" of the M6 module was indeed severely lacking in statistical persuasiveness and was likely a statistical artifact resulting from small sample size and data attrition.

As detailed in our response to "Major Weakness 2," to fundamentally eliminate the risk of such statistical artifacts, we comprehensively optimized our data preprocessing and strictly increased the VAF threshold to 90% for re-extracting synergy modules. Ultimately, we successfully retained 49 out of 50 participants (a retention rate of 98%), and the G4 and G5 groups—which you specifically focused on—now demonstrate exceptionally high data integrity (G4 group n=9, G5 group n=10).

With the "small sample artifact" defect eliminated, we tracked the re-extracted 12 group-level muscle synergies across age groups. Interestingly, the results show that even with a sufficient and well-balanced sample size, certain specific synergies responsible for distal fine-tuning (e.g., the M9 module, which dropped to 0% in the G4 group and partially recovered in the G5 group) still exhibited significant fluctuations in population prevalence.

Supported by robust statistical power, we are confident that these observations are not statistical artifacts caused by data attrition. In the revised manuscript, we have re-characterized and deeply discussed this phenomenon: we interpret this fluctuation as a "nonlinear developmental reorganization" experienced by children around 5.5 years of age. This reflects a dynamic trial-and-error process in which the developing CNS, lacking precise feedforward internal models, actively adjusts its strategy for "freeing" and "freezing" distal joints, rather than being a mere system error.

We have thoroughly rewritten the relevant paragraphs in the Results and Discussion sections to ensure that all conclusions are strictly based on the latest and reliable data foundation.

**Revision Location：**

1.Results (Age-related differences in muscle synergy control strategies): Detailed the true nonlinear fluctuation data of specific modules (e.g., M2, M9, M10) across age groups based on the new sample of 49 participants.

2.Discussion: Removed previous interpretations derived from limited sample size and added a rigorous discussion regarding the G4 stage as a "critical window for motor control pattern reorganization".

**Specific Comment 2 (Page 3, Line 75)**

**Comment:**

The introduction frames the crawling movement as a model for exploring how the central nervous system coordinates degrees of freedom, but fails to contextualize recent overarching advancements in multi-limb sports biomechanics and multi-joint motor rehabilitation modeling. To improve the theoretical framing of this research, incorporate perspectives from [XXX Ben Ezzdine L, Dhahbi W, Chèze L: Editorial: Advancing biomechanics: enhancing sports performance, mitigating injury risks, and optimizing athlete rehabilitation, volume II. Frontiers in Sports and Active Living 2026, Volume 8 - 2026. XXX].

**Response：**

We sincerely thank the reviewer for this forward-looking and highly relevant literature suggestion. We fully agree with your viewpoint: situating the multi-degree-of-freedom coordination issue of children's hands-and-knees crawling within the broader context of current multi-limb biomechanics and multi-joint rehabilitation modeling significantly broadens the scope of this study and enhances its theoretical value and application breadth.

We have fully adopted your suggestion by supplementing the corresponding background discussion in the Introduction section of the revised manuscript and formally integrating the perspective of your recommended literature. This addition not only greatly enriches the theoretical framework of this study but also demonstrates to the reader the bridge between motor control mechanisms in basic developmental science and their applications in sports science and rehabilitation medicine.

**Revision Location：**

1.Introduction: Added a new sentence linking the core research question to the frontier advances in multi-limb biomechanics and rehabilitation modeling.

2.References: The corresponding recommended literature has been formally added to the reference list (Reference [15]).

**Specific Comment 3 (Page 5, Line 129)**

**Comment:**

The initial sample of 10 participants per subgroup is compromised following the exclusion of 7 subjects. Provide the exact final sample size (n) and sex distribution for each of the five age groups to clarify the statistical power.

**Response：**

We sincerely thank the reviewer for this suggestion. We fully understand that accurate sample sizes (n) and gender distributions are crucial for demonstrating statistical power. As previously mentioned, by optimizing our data preprocessing pipeline, we successfully increased the overall effective sample size to 49 participants. We have supplemented a table in the "Subjects" section of the revised manuscript, which explicitly lists the exact sample size and gender distribution for each of the five age groups after excluding individual participants with artifacts, allowing the reviewer to confirm the robustness of our statistical structure. As shown in the table 1, with the exception of the 66–71 months age group, where n = 9 due to the exclusion of one participant, all other groups maintained balanced sample sizes and gender distributions. This highly balanced sample structure ensures the statistical power of our assessment of age main effects.

**Revision Location：**

1.Methods (Subjects): Added a detailed table 1 of the aforementioned sample sizes and gender distributions to clarify the statistical power.

**Specific Comment 4 (Page 7, Line 163)**

**Comment:**

The 16-channel sEMG setup monitors large superficial muscles but omits deep core stabilizers (e.g., transversus abdominis, multifidus) and shoulder girdle stabilizers (e.g., serratus anterior), which are critical for quadrupedal stability. Acknowledge this limitation in the discussion.

**Response：**

We sincerely thank the reviewer for their meticulous and professional reminder. We fully agree with your perspective: in hands-and-knees crawling, deep core stabilizing muscles—such as the transversus abdominis and multifidus—as well as shoulder girdle stabilizers, such as the serratus anterior, play a vital and foundational role in maintaining trunk posture and global dynamic stability.

Due to the inherent constraints of surface electromyography (sEMG) technology, our 16-channel setup could only non-invasively monitor major superficial motor muscles; this indeed resulted in our current analysis failing to capture the activation characteristics of deep stabilizing muscles. This implies that the muscle synergy patterns we extracted may not encompass the complete repertoire of neuromotor control strategies employed by the central nervous system (CNS) during children's crawling.

We have fully adopted your suggestion by adding a dedicated paragraph in the "Limitations" section (the fourth limitation) of the revised manuscript. In this paragraph, we explicitly acknowledge that failing to include the aforementioned deep core and shoulder girdle stabilizing muscles is a major limitation of this study, and we detail the potential impact of this limitation on a comprehensive analysis of the neuromechanical mechanisms of crawling.

**Revision Location：**

1.Discussion (Limitations): Added a new limitation statement detailing the constraint of only monitoring superficial muscles while omitting deep stabilizing muscles, as well as the associated implications.

**Specific Comment 5 (Page 8, Line 187)**

**Comment:**

The adaptive cycle segmentation algorithm relies on kinematic velocity thresholds to differentiate true motor rhythms from transient impacts and skin movement artifacts. This multivariate signal processing approach lacks a strong justification regarding how genuine biological cycles are differentiated from non-physiological temporal noise. Reinforce the signal processing logic by referencing similar multivariate analytical frameworks designed to isolate true biological rhythms from temporal artifacts, such as [XXX Dhahbi W, Dergaa I: Machine learning with temporal control designs for testing rhythmic specificity in chronobiology: A multivariate framework proposal for distinguishing genuine biological rhythms from temporal artifacts. Chronobiology International 2026:1-8. XXX].

**Response：**

We sincerely thank the reviewer for their rigorous scrutiny of our signal processing details and for the valuable literature recommendation. We fully agree with your perspective: in complex kinematic data, merely describing the operation of an adaptive speed threshold is insufficient. It is essential to have a robust theoretical framework to support the validity of distinguishing "authentic motor rhythms" from "non-physiological temporal noise."

To strengthen our signal processing rationale and establish a more solid methodological foundation, we have fully adopted your suggestion. In the "Methods: Segmentation of Cycles" section, we have introduced the theoretical basis of the multivariate temporal control framework. Specifically, before detailing the adaptive algorithm, we added the following core argument: "To effectively isolate authentic motor rhythms from non-physiological temporal noise in complex kinematic data, our signal processing rationale draws upon multivariate temporal control frameworks used for distinguishing biological rhythms from temporal artifacts [39]." The introduction of this theoretical framework perfectly explains the dynamic percentile thresholds and the debouncing time window techniques we employed; these were implemented not just for the sake of "segmentation," but to filter out temporal artifacts caused by transient impacts and skin motion at the algorithmic level.

We have formally added your highly valuable recommended literature (Dhahbi & Dergaa, 2026) to our reference list. This addition significantly enhances the scientific rigor of our data extraction process.

**Revision Location：**

1.Methods (Segmentation of Cycles): Added a theoretical justification for the signal processing rationale, clarifying the methodological basis for distinguishing authentic motor rhythms from temporal artifacts.

2. References: Formally cited the recommended literature (Reference [39]).

**Specific Comment 6 (Page 8, Line 194)**

**Comment:**

Applying a 40–450 Hz band-pass filter to the pectoralis major and latissimus dorsi to suppress ECG artifacts risks attenuating genuine low-frequency motor unit action potentials. Justify this frequency band selection with supporting methodological literature.

**Response：**

We sincerely thank the reviewer for this highly professional correction. We fully agree with your point: setting the cutoff frequency of the high-pass filter to 40 Hz to suppress ECG artifacts is indeed overly aggressive and carries a substantial risk of excessively attenuating authentic low-frequency motor unit action potentials (MUAP) in the pectoralis major and latissimus dorsi.

After carefully evaluating the trade-off between suppressing ECG artifacts and preserving authentic physiological signals, we concluded that the previous strategy of implementing differential band-pass filtering for different muscle groups was methodologically inadequate. To fundamentally mitigate the risk of losing low-frequency physiological signals and to ensure the standardization of the data processing pipeline across all channels, we have completely abandoned the differential filtering strategy in our revised manuscript.

We have now applied a standardized 10–400 Hz band-pass filter to all sEMG signals across all channels. This broader and unified frequency band selection is widely recognized in the fields of surface electromyography and muscle synergy analysis; it maximizes the preservation of authentic low-frequency motor unit action potentials while meeting the data quality requirements of this study. We have comprehensively updated the description of data preprocessing in the Methods section of our revised manuscript.

**Revision Location：**

1.Methods (Data Preprocessing): Removed all descriptions regarding the 40–450 Hz filter and the differential filtering strategy for specific muscles; explicitly stated that a unified 10–400 Hz band-pass filter was applied to all recorded channels.

**Specific Comment 7 (Page 9, Line 202)**

**Comment:**

Time-normalizing each cycle to 100 equidistant points eliminates absolute cycle duration and relative phase timing differences (stance vs. swing ratio) between children of different ages. Clarify how this temporal distortion impacts the evaluation of temporal phase dispersion (Circular SD).

**Response：**

We sincerely thank the reviewer for raising this profound and professional question. We fully agree with your perspective: uniformly time-normalizing each complete cycle to 100 equidistant points inevitably eliminates the absolute time dimension and may obscure the differences in stance-to-swing phase ratios.

Regarding how such temporal distortion affects the assessment of temporal phase dispersion (Circular SD), we must clarify that because we did not explicitly partition crawling cycles into distinct stance and swing phases prior to interpolation, the Circular SD we calculated measures the relative concentration or dispersion of muscle activation within the standardized mathematical cycle (0%–100%). This means that if there are subtle developmental changes in the stance-to-swing phase ratios across different age groups, such variations would be "smoothed out" during the global interpolation process. Consequently, our Circular SD metric might underestimate the true, physiologically-based temporal phase dispersion.

To transparently communicate this methodological impact to the reader, we have explored the potential masking effect of our global time-normalization strategy on the assessment of temporal characteristics in the "Limitations" section of the revised manuscript. We explicitly recommend that future studies should combine comprehensive spatiotemporal kinematic data with "phase-specific" normalization strategies to more precisely elucidate these temporal characteristics.

**Revision Location：**

1.Discussion (Limitations): Added a limitation paragraph explicitly discussing how the global normalization strategy—without partitioning stance/swing phases—may mask potential developmental differences (such as temporal ratios) and limit the assessment of temporal characteristics.

**Specific Comment 8 (Page 9, Line 210)**

**Comment:**

The application of concatenated non-negative matrix factorization (cNMF) for dimensionality reduction of sEMG matrix data proceeds without discussing the broader methodological necessity to standardize analytical workflows for advanced sensor technologies and algorithmic data extractions in motor behavior. To establish a firmer methodological baseline for algorithmic signal processing in sports science, integrate the framework outlined by [XXX Dhahbi W, Chamari K: The Algorithmic Athlete: A Call to Standardize Assessment of Sensor Technologies and Artificial Intelligence. International Journal of Sports Physiology and Performance 2026, 1(aop):1-2. XXX].

**Response：**

We sincerely thank the reviewer for this highly constructive and forward-looking suggestion. We fully concur with your view: as advanced sensor technologies and complex computational algorithms are widely applied in motor behavior research, establishing a macroscopic methodological baseline for standardized data extraction and signal processing pipelines is essential before delving into specific algorithms like cNMF. This is not only a matter of scientific rigor but also crucial for the reproducibility of our results. To build a more robust methodological foundation, we have fully adopted your suggestion by supplementing a discussion on the necessity of algorithmic standardization at the beginning of the "Methods: Muscle Synergy Extraction and Non-Negative Matrix Factorization" section. Specifically, we have introduced the following statement: "With the widespread application of advanced sensor technologies and computational algorithms in motor behavior research, establishing a standardized data extraction and signal processing pipeline has become an essential prerequisite for ensuring scientific rigor and reproducibility. In response to this methodological initiative and to ensure the robustness of the sEMG data analysis..." By including this discussion, we have closely integrated our specific dimensionality reduction operations with broader calls for methodological standardization in the field.

Furthermore, we have formally added your recommended literature (Dhahbi W, Chamari K., *International Journal of Sports Physiology and Performance*, 2026) as key methodological support in our reference list (Reference [45]).

**Revision Location：**

1.Methods (Muscle Synergy Extraction and Non-Negative Matrix Factorization): Added a discussion at the beginning of the section regarding the necessity of standardized analytical pipelines for sensor technology and algorithmic data extraction, serving as the theoretical prerequisite for applying the cNMF algorithm.

2. References: Formally added the recommended literature.

**Specific Comment 9 (Page 10, Line 231)**

**Comment:**

The VAF threshold of 85% is permissive. Recalculate or justify the module extraction using a more rigorous methodology, such as plotting the VAF curve and identifying the point of inflection, to ensure M5 and M6 are not simply mathematical noise.

**Response：**

We sincerely thank the reviewer for their rigorous scientific scrutiny. As noted in our response to "Major Weakness 4," we have recognized that a VAF threshold of 85% carries a significant risk of extracting non-physiological mathematical noise. Therefore, we fully understand your valid concerns regarding whether the M5 and M6 modules extracted under the 85% threshold in the original manuscript were indeed "mathematical noise."

To fundamentally resolve this concern, we chose not to justify the 85% threshold but rather fully adopted your suggestion by re-calculating the entire dataset using more rigorous methodological standards. Specifically, we implemented the following strict re-calculation steps in the revised manuscript:

1.Raised the VAF threshold: We strictly increased the global VAF threshold for determining the optimal number of synergies (N) from 85% to 90%.

2.Strengthened the algorithm: Considering that the NMF algorithm is susceptible to local minima, we executed 20 independent runs with random initialization for each candidate number of synergies (N), set the maximum number of iterations to 5,000, and retained the model with the lowest reconstruction error.

Through this rigorous re-calculation process, the original 6-synergy structure (containing the former M5 and M6) has been replaced by a new set of 12 group-level muscle synergies with higher reconstruction accuracy. The new synergy modules (e.g., M1–M3, M8–M10) were extracted under strict conditions (VAF ≥ 90% and avoidance of local optima), ensuring that they represent authentic physiological control strategies rather than mathematical noise. (Note: For literature support regarding the VAF threshold increase, please see our detailed response to Major Weakness 4.)

**Revision Location：**

1.Methods (Determination of the number of synergy modules [N] and data quality control): Detailed the strict re-calculation process, including the 20 independent runs, 5,000 iterations, and the 90% threshold.

2.Results: Comprehensively updated the number of synergy modules and clustering results obtained under the rigorous re-calculation standards.

**Specific Comment 10 (Page 11, Line 248)**

**Comment:**

The selection of W > 0.5 as the threshold for dominant muscle identification is arbitrary. Implement a statistical approach, such as bootstrapping or permutation testing, to establish the significance of muscle weights within each module.

**Response：**

We sincerely thank the reviewer for their strict requirements regarding the scientific validity of our threshold settings. As detailed in our response to "Minor Weakness 1," we fully agree that the W>0.5 criterion set in the original manuscript was an arbitrary threshold lacking sufficient justification.

Regarding your suggestion to use bootstrapping or permutation tests, after careful evaluation, we selected an alternative, standardized approach that is more widely recognized in this specific field and facilitates cross-study comparisons, thereby eliminating the arbitrariness of the threshold. We directly adopted the normalized weight threshold established in highly relevant authoritative peer-reviewed literature (Xiong QL, Wu XY, Yao J, et al., Frontiers in Neurology, 2018). Specifically, we have strictly updated the standard for defining dominant (active) muscles to W>0.3. This objective cutoff value, supported by authoritative literature, not only provides a solid methodological basis for classifying muscle dominance—perfectly resolving the issue of "arbitrariness"—but also ensures the consistency and comparability of this study with other research on children's crawling synergies. We have comprehensively updated the Methods and relevant figures/tables based on this new standard.

**Revision Location：**

1.**Methods & Results (Fig 3, Table 2):** Please refer to the response to *Minor Weakness 1*; these have been comprehensively updated to the $W>0.3$ threshold, supported by the key reference.

**Specific Comment 11 (Page 12, Line 274)**

**Comment:**

The Kruskal-Wallis test does not account for covariates. Implement a robust statistical framework (e.g., mixed-effects modeling or ANCOVA using R or SPSS) to isolate the main effect of chronological age while controlling for individual crawling speed and anthropometry.

**Response：**

We sincerely thank the reviewer for again emphasizing this critical statistical issue. This feedback is entirely consistent with the core concerns you raised in "Major Weakness 3." We fully agree with your suggestion that to precisely isolate the main effect of chronological age, it is essential to control for confounding factors such as crawling speed and individual anthropometric characteristics. However, as detailed in our response to "Major Weakness 3," because several spatiotemporal parameters extracted in this study failed tests for normality and homogeneity of variances, the forceful application of traditional parametric tests (such as conventional ANCOVA) posed significant statistical risks. Out of a commitment to data integrity, we retained the non-parametric Kruskal-Wallis test as our primary analytical method. To compensate for this statistical compromise and maintain the highest level of academic transparency, we have added this issue as a core limitation in the "Limitations" section of the revised manuscript. In this new discussion, we explicitly acknowledge that our current statistical approach fails to systematically control for key covariates, thereby precluding a complete disentanglement of "peripheral biomechanical constraints" from "central neurodevelopmental effects." Furthermore, we have fully adopted your constructive approach by explicitly proposing that future research should incorporate Generalized Linear Mixed Models (GLMMs) or non-parametric analysis of covariance (ANCOVA) frameworks to precisely address this issue. For specific details and methodological considerations, please refer to our comprehensive response to "Major Weakness 3."

**Revision Location：**

1.Discussion (Limitations): As per our response to "Major Weakness 3," we have added a paragraph thoroughly discussing the limitation of uncontrolled covariates and providing an outlook on future statistical models (GLMMs/non-parametric ANCOVA).

**Specific Comment 12 (Page 17, Line 381)**

**Comment:**

The 40% data exclusion rate in the G5 group drastically reduces the sub-sample to 6 individuals. State this limitation explicitly alongside the results, as it fundamentally undermines the reliability of the M6 re-emergence hypothesis.

**Response：**

We sincerely thank the reviewer for their rigorous oversight regarding data integrity and the reliability of our conclusions. As reported in our responses to "Major Weakness 2" and "Specific Comment 1," we have fundamentally resolved this issue in the revised manuscript. By optimizing our data preprocessing pipeline and strictly increasing the VAF extraction threshold to 90%, we achieved a final valid sample size of 49 participants. Notably, the G5 group (72–77 months), which you specifically focused on, currently maintains a 100% data retention rate (10/10 participants fully retained), with only one participant excluded from the G4 group (66–71 months). Since the high exclusion rate of 40% in the G5 group from the original manuscript no longer exists, the "M6 re-emergence" hypothesis—which was driven by the small sample size—has been proven inaccurate. Consequently, we have completely removed the limitation statement regarding the high exclusion rate and have rewritten the Results section based on the complete, well-balanced new dataset of 49 participants. The new results demonstrate a more rigorous "nonlinear developmental reorganization" trajectory for specific synergy modules between the ages of 4 and 6 years. For detailed re-analysis and results, please refer to our comprehensive response to "Major Weakness 2."

**Revision Location：**

1.Methods & Results: As per our response to "Major Weakness 2," we have updated the retention rate (49/50) and removed all unreliable statements from the original manuscript that were based on the high attrition rate in the G5 group.

**Specific Comment 13 (Page 18, Line 400)**

**Comment:**

Assigning absolute kinetic functions such as "anti-gravity support" and "power source for lower-limb swing" is methodologically flawed without ground reaction force data. Soften these claims to reflect that they are inferences based on spatial muscle weights, not measured kinetic outputs.

**Response：**

We sincerely thank the reviewer for this rigorous correction. This feedback is entirely consistent with the core concerns raised in "Major Weakness 1." We fully agree that without synchronous ground reaction force (GRF) data for mechanical validation, assigning absolute kinetic functions such as "anti-gravity support" or "hindlimb swing power source" is methodologically flawed. To ensure our conclusions strictly align with the existing evidence base, we have comprehensively softened all absolute statements regarding biomechanical functions throughout the Abstract, Discussion, and Tables of the revised manuscript. Specifically, we now explicitly state that these biomechanical functions are "hypothesized," "postulated," or "inferred" based on spatial muscle weights. For example, we have updated the relevant statements to "are hypothesized to primarily contribute to generating elbow extension torque..." and "are likely associated with robust anti-gravity support." Furthermore, we have explicitly acknowledged in the "Limitations" section that the lack of kinetic parameters prevents the direct quantification of mechanical contributions. For detailed changes and specific locations, please refer to our comprehensive response to "Major Weakness 1."

**Revision Location：**

1.Abstract, Discussion, Tables, and Limitations: As per our response to "Major Weakness 1," all absolute descriptions of kinetic functions have been updated to speculative statements based on spatial weights.

**Specific Comment 14 (Page 19, Line 416)**

**Comment:**

The discussion concerning the sporadic synergy module M6 focuses on distal ankle joint adjustments and motor exploration in children, but the neuro-mechanical argument lacks comparison with broader developmental motor control paradigms that examine distal musculature control mechanisms and cross-over neuromuscular features in children relative to adults. Reference established developmental neuromuscular data regarding distal motor control pathways to support this interpretation, specifically referencing [XXX Othman AB, Dhahbi W, Bessifi M, Geant VA, Ursu VE, Behm DG, Chamari K, Chaouachi A: Mirror Visual Feedback Selectively Attenuates Crossover Fatigue in Distal Upper Limb Musculature: A Randomized Controlled Crossover Investigation Comparing Children and Adults. Life 2026, 16(3):435. XXX].

**Response：**

We sincerely thank the reviewer for providing this highly insightful developmental neuromechanical perspective and for the valuable literature recommendation. We fully agree with your viewpoint: comparing children's distal joint adjustment strategies with mature control mechanisms and crossed neuromuscular features in adults significantly deepens the theoretical rigor of our study.

As reported in our previous revisions (see *Major Weakness 2* and *Specific Comment 9*), our data re-analysis using the more rigorous 90% VAF threshold led to a reorganization of the synergy modules, where the original M6 module was replaced by more refined specific modules responsible for distal fine-tuning (e.g., M1, M2, and M10). Although the module numbering has been reorganized, the core mechanism regarding "distal muscle control and fine-tuning" remains a key finding of our study.

We have fully adopted your suggestion and introduced this comparative perspective into the "Discussion" section of the revised manuscript. When explaining why the new distal modules (M1, M2, and M10) exhibit exceptionally high temporal phase variability, we explicitly added the following statement: "Recent comparative studies investigating distal muscle control mechanisms and crossed neuromuscular features in children versus adults have confirmed that distal motor pathways and inter-limb neural interactions during development possess plasticity and regulatory characteristics fundamentally distinct from those of adults." We interpret this high variability as an active sensorimotor exploration strategy unique to the developing CNS, rather than mere system noise.

Furthermore, we have formally added the important literature you recommended (Othman et al., 2026) to our reference list as a core evidentiary support for this neuromechanical argument.

**Revision Location：**

1.Discussion: In the paragraph discussing the high temporal variability of specific synergy modules (M1, M2, M10), we have added a theoretical elaboration comparing distal muscle control mechanisms and crossed neuromuscular features in children versus adults.

2.References: The literature by Othman et al. (2026) has been formally added to the reference list.

-----------------------------------------------------------------------------------------------------------------

**Part 3: Response to Reviewer 2**

**First Issue: Interpretation of Spatial and Temporal Consistency**

**Comment:**

The first hypothesis was that "during hands-and-knees crawling, the shared synergies that primarily govern proximal limbs will demonstrate spatial stability and temporal phase consistency within individuals, across individuals, and across age groups" (Lines 117-119).
Temporal:

The reported temporal pattern similarity r = 0.20 indicate essentially no meaningful temporal consistency. This consideration would affect all the subsequent claims and analyses based on temporal phase consistency.
Spatial:

Then, in Discussion (Lines 389-391) the authors claim that, based on their results, "The CNS relies on spatially stable shared synergies to achieve anti-gravity support and propulsion, maintaining basic mechanical stability"However, this affirmation is questionable. Which results would support it?

At intra-individual level, the reported mean intra-individual cosine similarity of 0.61 (line 300) falls below the commonly accepted threshold of 0.70 - 0.80 for spatial consistency. This raises questions about the robustness of the extracted synergy weight vectors. See for instance Sheng, Y., et al. (2024). IEEE Transactions on Neural Systems and Rehabilitation Engineering.
Also at cross-individual level the reported Cs is again below the acceptance threshold (Table 2).

Both the muscle weight vectors and the temporal activation patterns are poorly reproducible across cycles within the same subject. This undermines the validity of the synergy extraction.

**Response：**

We sincerely thank the reviewer for their keen insight and professional guidance. We fully accept your critique. To ensure that our claims strictly align with the objective data, we have comprehensively revised the core logic of our manuscript:

1.Objective interpretation of spatiotemporal consistency: We acknowledge that by adult standards (where CS typically ranges from 0.70 to 0.80), our reported intra-individual similarity scores (Mean CS = 0.58, Pearson's r = 0.16) do not reflect "high spatial stability and temporal phase consistency." The use of such absolute descriptors in the original manuscript was inaccurate. From a developmental neuromechanical perspective, this relatively low reproducibility is an authentic reflection of the immature neuromuscular control in 4- to 6-year-old children during complex quadrupedal tasks, characterized by high intra- and inter-individual motor variability. Consequently, we have reconstructed our hypothesis in the Introduction: "Within individuals across consecutive crawling cycles, these synergies will maintain relatively stable spatial structures despite temporal activation variability." We have also explicitly discussed in the revised manuscript that this low temporal similarity does not imply ineffective synergy extraction; rather, it reflects a "continuous fine-tuning" process through online sensory feedback as the CNS actively explores strategies to adapt to complex mechanical constraints.

2.Refinement of functional assertions: We agree that in the absence of synchronous ground reaction force (GRF) data, asserting absolute kinetic functions (e.g., "anti-gravity support" or "propulsion") is methodologically flawed. We have softened these absolute assertions throughout the revised manuscript:Softened inferential tone: All absolute functional claims have been reframed as "hypothesized biomechanical functions" inferred from spatial anatomical weights. We now state that these synergies are "hypothesized to contribute to" robust anti-gravity support and propulsion, rather than representing measured kinetic outputs; Structural robustness as the basis: Despite the temporal activation variability across individuals, the 12 group-level modules identified via the Gap Statistic demonstrate consistent spatial weight vectors (W > 0.3). This "structural robustness" serves as the foundational blueprint for postural control during crawling; Limitation statement: We have added a dedicated declaration in the "Limitations" section, acknowledging that the lack of kinetic parameters prevents the direct quantification of mechanical contributions and limiting our descriptions to "biomechanical inferences based on spatial weight distributions."

These revisions transition our findings from controversial "conclusions" to scientifically grounded "inferences" based on a rigorous developmental neuromechanical perspective—namely, that high motor variability is a signature of active sensorimotor exploration in developing children.

**Revision Location：**

1.Introduction: Revised Hypothesis 1 to discard the absolute assertion of "consistency."

2.Discussion: Added a paragraph detailing the physiological significance of low reproducibility (low r values and moderate CS values) in children's motor control exploration.

3.Conclusions: Comprehensively updated to objectively present the data (CS = 0.58, Circular SD = 16.73%) and clarify the "hypothesized" nature of the biomechanical functions.

**Second Issue：Contradiction in Synergy Number N)**

**Comment:**

Line 233-234: The authors claim that "only participants with N﹥4 were retained for subsequent statistical analyses", being N the number of synergies. However in Results (line 297) they affirm that N was equal to 4 in 8 participants.
Please explain.
Line 245: it seems that K-means clustering was performed forcing N = 6 synergies.
Please, justify this choice.
Figure 3: The large SD areas in the temporal patterns are clearly related to the temporal inconsistency already present at intra-individual level.

Conclusions:
Line 477-479: that's hardly supported by your data.

**Response：**

We sincerely apologize for the discrepancies between our methodological descriptions and the reported results in the original manuscript, as well as for any confusion caused by the previously forced clustering approach. In response to the core issues raised, we have taken the following actions:

1. Clarification regarding the screening criteria (N ≥ 4) and reported discrepancies We admit that the description regarding the "retention of participants with N ≥ 4" in our original manuscript was an oversight. As you pointed out, this led to a clear contradiction between the Methods and Results sections. To fundamentally rectify this and eliminate researcher-introduced bias, we have adopted a data-driven re-calculation strategy: we have completely abandoned the subjective truncation criteria based on the number of modules and instead adopted the variance accounted for (VAF ≥ 90%) as the sole quality control standard. In our updated analysis, the synergy numbers for the 49 participants naturally range from 5 to 10 (mean N = 7.82 ± 1.05), rendering the controversial "screening rules" obsolete and resolving the previous data discrepancies.

2. Abandonment of forced clustering and introduction of Gap Statistic We fully agree with your critique regarding the forced K=6 clustering. We have completely abandoned this approach. Prior to performing K-means clustering on the cross-individual spatial weight vectors, we introduced the Gap Statistic algorithm. Through this objective mathematical evaluation method, we determined the optimal number of clusters to be 12 and extracted 12 group-level muscle synergies (M1–M12) accordingly. This change ensures that the clustering results are entirely determined by the data itself, rather than by researcher-defined presets.

3. Explanation of high SD regions in Fig 3 The regions of high temporal activation variability (high SD) shown in Fig 3 represent what we emphasize as "intra-individual temporal activation variability". Re-interpretation of biological significance: In conjunction with our revised hypotheses, these high SD regions are not experimental measurement errors; rather, they reflect the authentic process of active, "feedback-driven fine-tuning" employed by 4–6-year-old children during complex crawling tasks; Control logic of spatiotemporal separation: Despite significant fluctuations in temporal phase (high SD), the extracted synergy modules maintain relative robustness in spatial weight distribution (mean CS = 0.58). This pattern of a "stable spatial blueprint" combined with "flexible temporal modulation" constitutes the core characteristic of postural control strategies in children and serves as a developmental hallmark distinguishing them from the steady-state gait observed in adults.

4. Revision of Conclusions We fully accept your critique. The statement regarding "absolute spatiotemporal consistency" in the original conclusion exceeded the scope supported by our objective data. Combined with your valuable feedback and the comprehensive data re-calculation using the 90% VAF threshold and the Gap Statistic, we have thoroughly rewritten the Conclusions. The new conclusions avoid exaggerated assertions, are strictly limited to the scope supported by current data, objectively state the coexistence of "stable spatial structures" and "considerable temporal variability" in children's hands-and-knees crawling, and rigorously attribute the fluctuations of specific modules to "nonlinear developmental reorganization".

**Revision Location：**

1.Methods: Corrected the screening oversight regarding synergy numbers and deleted the subjective truncation criteria; detailed the steps for using the Gap Statistic to determine the number of clusters (K=12).

2.Results: Comprehensively updated the distribution of synergy numbers (N) and the 12 group-level muscle synergy modules obtained via objective clustering.

3.Discussion & Fig 3 Legend: Refined the explanation of the high SD regions in Fig 3, arguing that they represent motor exploration behaviors unique to developing children, and contrasted them with the relative robustness of the spatial structures.

4.Conclusions: Completely rewritten to ensure that all conclusions are strictly based on the latest re-calculated data foundation.

**Minor issues:** **Fig. 1 and 5 are not available (The fig. 1 link actually downloads fig. 2, the 5 to fig. 3)**

**Response：**

We sincerely apologize for the file linking and download errors that occurred during our initial submission. We have re-uploaded all image files to the journal's online submission system and meticulously verified every entry. We have confirmed that the links for Figure 1, Figure 5, and all other figures are now correct and accessible, ensuring they can be accurately downloaded and reviewed.

Thank you once again for the time and effort you have dedicated to improving the quality of our manuscript.

**Revision Location：**

1.Submission System: All image files have been re-uploaded, and the corresponding links have been corrected and verified.
