# Supplementary material for: Muscle synergy characteristics during hands-and-knees crawling in children aged 4–6 years
Source: PLoS One. 2026 Oct 8;21(10):e0358587. doi: 10.1371/journal.pone.0358587 (PMC13649141; doi:10.1371/journal.pone.0358587)
Supplement: Peer review history

# Peer review: Decision Letter 0

**DOI:** 10.1371/journal.pone.0358587.r001

**Authors:** Mário Espada

28 Jun 2026

Dear Dr. Jia,

Thank you for submitting your manuscript to PLOS ONE. After careful consideration, we feel that it has merit but does not fully meet PLOS ONE’s publication criteria as it currently stands. Therefore, we invite you to submit a revised version of the manuscript that addresses the points raised during the review process.

Please submit your revised manuscript by Aug 25 2026 11:59PM. If you will need more time than this to complete your revisions, please reply to this message or contact the journal office at plosone@plos.org. When you're ready to submit your revision, log on to https://www.editorialmanager.com/pone/ and select the 'Submissions Needing Revision' folder to locate your manuscript file.

- A letter that responds to each point raised by the academic editor and reviewer(s). You should upload this letter as a separate file labeled 'Response to Reviewers'.
- A marked-up copy of your manuscript that highlights changes made to the original version. You should upload this as a separate file labeled 'Revised Manuscript with Track Changes'.
- An unmarked version of your revised paper without tracked changes. You should upload this as a separate file labeled 'Manuscript'.

If you would like to make changes to your financial disclosure, please include your updated statement in your cover letter. Guidelines for resubmitting your figure files are available below the reviewer comments at the end of this letter.

If applicable, we recommend that you deposit your laboratory protocols in protocols.io to enhance the reproducibility of your results. Protocols.io assigns your protocol its own identifier (DOI) so that it can be cited independently in the future. For instructions see: https://journals.plos.org/plosone/s/submission-guidelines#loc-laboratory-protocols. Additionally, PLOS ONE offers an option for publishing peer-reviewed Lab Protocol articles, which describe protocols hosted on protocols.io. Read more information on sharing protocols at https://plos.org/protocols?utm_medium=editorial-email&utm_source=authorletters&utm_campaign=protocols.

As the corresponding author, your ORCID iD is verified in the submission system and will appear in the published article. PLOS supports the use of ORCID, and we encourage all coauthors to register for an ORCID iD and use it as well. Please encourage your coauthors to verify their ORCID iD within the submission system before final acceptance, as unverified ORCID iDs will not appear in the published article. *Only*  the individual author can complete the verification step; PLOS staff *cannot*  verify ORCID iDs on behalf of authors.

We look forward to receiving your revised manuscript.

Kind regards,

Mário Espada, PhD

Academic Editor

PLOS One

Journal Requirements:

When submitting your revision, we need you to address these additional requirements.

1. Please ensure that your manuscript meets PLOS ONE's style requirements, including those for file naming. The PLOS ONE style templates can be found at

https://journals.plos.org/plosone/s/file?id=wjVg/PLOSOne_formatting_sample_main_body.pdf and

https://journals.plos.org/plosone/s/file?id=ba62/PLOSOne_formatting_sample_title_authors_affiliations.pdf

2. Please note that PLOS One has specific guidelines on code sharing for submissions in which author-generated code underpins the findings in the manuscript. In these cases, all author-generated code must be made available without restrictions upon publication of the work. Please review our guidelines at https://journals.plos.org/plosone/s/materials-and-software-sharing#loc-sharing-code and ensure that your code is shared in a way that follows best practice and facilitates reproducibility and reuse.

3. Please update your submission to use the PLOS LaTeX template. The template and more information on our requirements for LaTeX submissions can be found at http://journals.plos.org/plosone/s/latex.

4. If the reviewer comments include a recommendation to cite specific previously published works, please review and evaluate these publications to determine whether they are relevant and should be cited. There is no requirement to cite these works unless the editor has indicated otherwise.

Additional Editor Comments:

Dear Authors,

Congratulations on your work.

Please revise the manuscript in light of the reviewers' major suggestions.

Thank you.

Best regards.

[Note: HTML markup is below. Please do not edit.]

Reviewers' comments:

Reviewer's Responses to Questions

**Comments to the Author**

1. Is the manuscript technically sound, and do the data support the conclusions?

Reviewer #1: Partly

Reviewer #2: Partly

**********

2. Has the statistical analysis been performed appropriately and rigorously?

Reviewer #1: Yes

Reviewer #2: Yes

**********

3. Have the authors made all data underlying the findings in their manuscript fully available?

The PLOS Data policy

Reviewer #1: Yes

Reviewer #2: Yes

**********

4. Is the manuscript presented in an intelligible fashion and written in standard English?

Reviewer #1: Yes

Reviewer #2: Yes

**********

Reviewer #1: General Comments

Major Weaknesses

Lack of Kinetic Validation: The study relies exclusively on surface electromyography (sEMG) and kinematic trajectories to map muscle synergies during hands-and-knees crawling. Making definitive functional claims about "anti-gravity support" and "forward propulsion" without synchronized kinetic data (such as ground reaction forces from force platforms) renders the biomechanical interpretations speculative.

High Attrition Rate and Sub-sample Validity: The exclusion of 7 out of 50 participants significantly impacts the sub-group distributions. Specifically, the 40% exclusion rate in the G5 (72–77 months) group drastically lowers the statistical power of the developmental claims made regarding the "re-emergence" of the M6 synergy.

Statistical Modeling Deficiencies: The statistical approach relies on basic non-parametric tests (Kruskal-Wallis) to assess age group differences. This method fails to control for critical confounding covariates such as crawling speed, stride length, and individual anthropometrics, which fundamentally dictate muscle synergy temporal activation and spatial composition.

Permissive VAF Threshold: The variance accounted for (VAF) threshold of 85% is comparatively low for concatenated non-negative matrix factorization (cNMF) in locomotion studies, which typically employ a 90% or higher threshold. This permissive cutoff risks overfitting and extracting non-physiological movement noise as valid "sporadic" synergies.

Minor Weaknesses

Arbitrary Muscle Dominance Threshold: The use of an arbitrary threshold (W > 0.5) to define dominant muscles within a module lacks a rigorous mathematical foundation.

Data Interpolation Risks: The uniform time-normalization to 100 points via PCHIP interpolation removes temporal variations in stance/swing phase ratios across the different age groups, potentially masking crucial developmental differences in motor control timing.

Specific Comments

Page 2, Line 46: The claim that the distal fine-tuning synergy module (M6) was absent (0.0% detection rate) in the 66–71-month age group but re-emerged in the 72–77-month group relies on extremely small and unequal sub-samples after data exclusion. Address the high probability that this is a statistical artifact rather than a genuine physiological phenomenon.

Page 3, Line 75: The introduction frames the crawling movement as a model for exploring how the central nervous system coordinates degrees of freedom, but fails to contextualize recent overarching advancements in multi-limb sports biomechanics and multi-joint motor rehabilitation modeling. To improve the theoretical framing of this research, incorporate perspectives from [XXX Ben Ezzdine L, Dhahbi W, Chèze L: Editorial: Advancing biomechanics: enhancing sports performance, mitigating injury risks, and optimizing athlete rehabilitation, volume II. Frontiers in Sports and Active Living 2026, Volume 8 - 2026. XXX].

Page 5, Line 129: The initial sample of 10 participants per subgroup is compromised following the exclusion of 7 subjects. Provide the exact final sample size ($n$) and sex distribution for each of the five age groups to clarify the statistical power.

Page 7, Line 163: The 16-channel sEMG setup monitors large superficial muscles but omits deep core stabilizers (e.g., transversus abdominis, multifidus) and shoulder girdle stabilizers (e.g., serratus anterior), which are critical for quadrupedal stability. Acknowledge this limitation in the discussion.

Page 8, Line 187: The adaptive cycle segmentation algorithm relies on kinematic velocity thresholds to differentiate true motor rhythms from transient impacts and skin movement artifacts. This multivariate signal processing approach lacks a strong justification regarding how genuine biological cycles are differentiated from non-physiological temporal noise. Reinforce the signal processing logic by referencing similar multivariate analytical frameworks designed to isolate true biological rhythms from temporal artifacts, such as [XXX Dhahbi W, Dergaa I: Machine learning with temporal control designs for testing rhythmic specificity in chronobiology: A multivariate framework proposal for distinguishing genuine biological rhythms from temporal artifacts. Chronobiology International 2026:1-8. XXX].

Page 8, Line 194: Applying a 40–450 Hz band-pass filter to the pectoralis major and latissimus dorsi to suppress ECG artifacts risks attenuating genuine low-frequency motor unit action potentials. Justify this frequency band selection with supporting methodological literature.

Page 9, Line 202: Time-normalizing each cycle to 100 equidistant points eliminates absolute cycle duration and relative phase timing differences (stance vs. swing ratio) between children of different ages. Clarify how this temporal distortion impacts the evaluation of temporal phase dispersion (Circular SD).

Page 9, Line 210: The application of concatenated non-negative matrix factorization (cNMF) for dimensionality reduction of sEMG matrix data proceeds without discussing the broader methodological necessity to standardize analytical workflows for advanced sensor technologies and algorithmic data extractions in motor behavior. To establish a firmer methodological baseline for algorithmic signal processing in sports science, integrate the framework outlined by [XXX Dhahbi W, Chamari K: The Algorithmic Athlete: A Call to Standardize Assessment of Sensor Technologies and Artificial Intelligence. International Journal of Sports Physiology and Performance 2026, 1(aop):1-2. XXX].

Page 10, Line 231: The VAF threshold of 85% is permissive. Recalculate or justify the module extraction using a more rigorous methodology, such as plotting the VAF curve and identifying the point of inflection, to ensure M5 and M6 are not simply mathematical noise.

Page 11, Line 248: The selection of W > 0.5 as the threshold for dominant muscle identification is arbitrary. Implement a statistical approach, such as bootstrapping or permutation testing, to establish the significance of muscle weights within each module.

Page 12, Line 274: The Kruskal-Wallis test does not account for covariates. Implement a robust statistical framework (e.g., mixed-effects modeling or ANCOVA using R or SPSS) to isolate the main effect of chronological age while controlling for individual crawling speed and anthropometry.

Page 17, Line 381: The 40% data exclusion rate in the G5 group drastically reduces the sub-sample to 6 individuals. State this limitation explicitly alongside the results, as it fundamentally undermines the reliability of the M6 re-emergence hypothesis.

Page 18, Line 400: Assigning absolute kinetic functions such as "anti-gravity support" and "power source for lower-limb swing" is methodologically flawed without ground reaction force data. Soften these claims to reflect that they are inferences based on spatial muscle weights, not measured kinetic outputs.

Page 19, Line 416: The discussion concerning the sporadic synergy module M6 focuses on distal ankle joint adjustments and motor exploration in children, but the neuro-mechanical argument lacks comparison with broader developmental motor control paradigms that examine distal musculature control mechanisms and cross-over neuromuscular features in children relative to adults. Reference established developmental neuromuscular data regarding distal motor control pathways to support this interpretation, specifically referencing [XXX Othman AB, Dhahbi W, Bessifi M, Geantă VA, Ursu VE, Behm DG, Chamari K, Chaouachi A: Mirror Visual Feedback Selectively Attenuates Crossover Fatigue in Distal Upper Limb Musculature: A Randomized Controlled Crossover Investigation Comparing Children and Adults. Life 2026, 16(3):435. XXX].

Reviewer #2: The paper address an interesting topic. Hands and knees crawling is biomechanically different from other gaits, and yet is a crucial stage in development.

The authors employed an appropriate and consistent methodology, in a well designed set-up.

However, I have some concerns about the interpretation of some results, that would affect all the subsequent choice and further interpretation and conclusions.

I resumed my concerns in two main issues:

First Issue

The first hypothesis was that "during hands-and-knees crawling, the shared synergies that primarily govern proximal limbs will demonstrate spatial stability and temporal phase consistency within individuals, across individuals, and across age groups" (Lines 117-119).

Temporal:

The reported temporal pattern similarity r = 0.20 indicate essentially no meaningful temporal consistency. This consideration would affect all the subsequent claims and analyses based on temporal phase consistency.

Spatial:

Then, in Discussion (Lines 389-391) the authors claim that, based on their results, "The CNS relies on spatially stable shared synergies to achieve anti-gravity support and propulsion, maintaining basic mechanical stability".

However, this affirmation is questionable. Which results would support it?

At intra-individual level, the reported mean intra-individual cosine similarity of 0.61 (line 300) falls below the commonly accepted threshold of 0.70 - 0.80 for spatial consistency. This raises questions about the robustness of the extracted synergy weight vectors. See for instance Sheng, Y., et al. (2024). IEEE Transactions on Neural Systems and Rehabilitation Engineering.

Also at cross-individual level the reported Cs is again below the acceptance threshold (Table 1).

Both the muscle weight vectors and the temporal activation patterns are poorly reproducible across cycles within the same subject. This undermines the validity of the synergy extraction.

Second Issue

Line 233-234: The authors claim that "only participants with N﹥4 were retained for subsequent statistical analyses", being N the number of synergies. However in Results (line 297) they affirm that N was equal to 4 in 8 participants.

Please explain.

Line 245: it seems that K-means clustering was performed forcing N = 6 synergies.

Please, justify this choice.

Figure 3: The large SD areas in the temporal patterns are clearly related to the temporal inconsistency already present at intra-individual level.

Conclusions:

Line 477-479: that's hardly supported by your data.

Minor issues:

Fig. 1 and 5 are not available (The fig. 1 link actually downloads fig. 2, the 5 to fig. 3)

**********

what does this mean?). If published, this will include your full peer review and any attached files.

If you choose “no”, your identity will remain anonymous but your review may still be made public.

**Do you want your identity to be public for this peer review?**  For information about this choice, including consent withdrawal, please see our Privacy Policy

Reviewer #1: **Yes:**  Wissem Dhahbi

Reviewer #2: No

**********

[NOTE: If reviewer comments were submitted as an attachment file, they will be attached to this email and accessible via the submission site. Please log into your account, locate the manuscript record, and check for the action link "View Attachments". If this link does not appear, there are no attachment files.]

To ensure your figures meet our technical requirements, please review our figure guidelines: https://journals.plos.org/plosone/s/figures

You may also use PLOS’s free figure tool, NAAS, to help you prepare publication quality figures: https://journals.plos.org/plosone/s/figures#loc-tools-for-figure-preparation.

NAAS will assess whether your figures meet our technical requirements by comparing each figure against our figure specifications.

# Author comment: Author response to Decision Letter 1

**DOI:** 10.1371/journal.pone.0358587.r002

13 Jul 2026

Part 2: Response to Reviewer 1

Comment 1 (Major Weakness 1: Lack of Kinetic Validation)

Comment:

The study relies exclusively on surface electromyography (sEMG) and kinematic trajectories to map muscle synergies during hands-and-knees crawling. Making definitive functional claims about "anti-gravity support" and "forward propulsion" without synchronized kinetic data (such as ground reaction forces from force platforms) renders the biomechanical interpretations speculative.

Response：

We sincerely thank the reviewer for this rigorous and insightful evaluation. We fully agree that assigning absolute kinetic functions (e.g., "anti-gravity support" or "forward propulsion") based solely on surface electromyography (sEMG) and kinematic data lacks direct mechanical validation. To ensure our conclusions strictly align with the available evidence, we have comprehensively softened these statements throughout the revised manuscript.

1.Softened inferential tone: In the Abstract and Discussion sections, we explicitly reframed these biomechanical roles as inferences. We introduced speculative phrases such as "hypothesized to contribute to," "postulated to perform," and "likely associated with." This is intended to clearly convey to the reader that these functions are inferred based on spatial anatomical weights rather than being measured kinetic outputs.

2.Modified table titles: We have rigorously updated the title of Table 2 to refer to "hypothesized biomechanical functions."

3.Added limitation statement: In the Limitations section of the Discussion, we now explicitly acknowledge the lack of synchronous GRF data as a major limitation of the present study. We specifically added the following statement: "Second, the investigation of neural control strategies relied primarily on surface electromyography (sEMG) signals without synchronous kinetic validation parameters. Consequently, mechanical contributions cannot be directly quantified."

Revision Location：

1.Abstract & Discussion: Comprehensively updated the speculative statements regarding biomechanical functions.

2.Results: The title of Table 2 has been modified.

3.Discussion (Limitations): Added a specific statement regarding the lack of kinetic validation parameters.

Comment 2 (Major Weakness 2: High Attrition Rate and Sub-sample Validity)

Comment:

The exclusion of 7 out of 50 participants significantly impacts the sub-group distributions. Specifically, the 40% exclusion rate in the G5 (72–77 months) group drastically lowers the statistical power of the developmental claims made regarding the "re-emergence" of the M6 synergy.

Response：

We sincerely thank the reviewer for astutely pointing out this critical statistical issue. We fully agree that the excessively high data attrition rate in the original manuscript (especially the high exclusion rate in the original G5 group) severely compromised the representativeness of the subsample, resulting in a lack of sufficient statistical power for our previous conclusions regarding the developmental trajectories of synergy modules.

To fundamentally resolve this issue and adequately address your concerns, we have taken the following key actions in this revision:

1.Optimizing data processing and raising the VAF threshold: We rigorously reviewed and optimized the preprocessing of the electromyographic (EMG) signals and algorithm parameters. Furthermore, the extraction threshold for the variance accounted for (VAF) was strictly increased from 85% to 90%, a more rigorous standard in motor dynamics research.

2.Exceptionally high data retention rate: Thanks to the optimized processing pipeline, out of the 50 initially recruited participants, only one participant in the 66–71 months age group was excluded due to electromyographic (EMG) artifacts. The final valid sample size included in the subsequent statistical analyses reached 49 participants (yielding an overall data retention rate of 98%). For the G5 (72–77 months) group, which you specifically expressed concern about, the data retention rate is now 100% (10/10).

3.Re-evaluating conclusions on developmental trajectories: Leveraging the complete and well-balanced new subsample data, we re-clustered and re-analyzed all results, yielding twelve group-level muscle synergies. Based on the current robust statistical power, we found that specific synergies (e.g., M2, M9, M10) did not simply exhibit an absolute "re-emergence" as stated in the original manuscript. Instead, their population prevalence underwent pronounced nonlinear developmental reorganization. We have thoroughly rewritten the relevant paragraphs in the Discussion and Conclusions sections of the revised manuscript to ensure that all conclusions are built upon a solid data foundation.

This major data re-analysis has greatly enhanced the reliability of our study, and we are deeply grateful for your constructive criticism.

Revision Location：

1.Abstract: Explicitly updated the final sample size included in the analysis to 49 healthy preschool children.

2.Materials and Methods (Subjects & Muscle Synergy Extraction): Detailed the data attrition showing only one participant was excluded , and added the methodological setting of increasing the optimal number of muscle synergies threshold to VAF ≥ 90%.

3.Results: Comprehensively updated the data based on the final 49 valid participants, completely removing the negative statements regarding the high exclusion rate in the G5 group from the original manuscript.

4.Discussion and Conclusions: In light of the latest data, replaced the previous "re-emergence" hypothesis, which resulted from limited sample size, with the more rigorous theory of nonlinear dynamic reorganization trajectories.

Comment 3 (Major Weakness 3: Statistical Modeling Deficiencies)

Comment:

The statistical approach relies on basic non-parametric tests (Kruskal-Wallis) to assess age group differences. This method fails to control for critical confounding covariates such as crawling speed, stride length, and individual anthropometrics, which fundamentally dictate muscle synergy temporal activation and spatial composition.

Response：

We sincerely thank the reviewer for this highly constructive and pertinent statistical feedback. We fully agree that key covariates, including crawling speed, stride length, and individual anthropometric characteristics, are critical confounding factors that ideally should be strictly controlled using analysis of covariance (ANCOVA) or mixed-effects models.

Upon receiving your insightful comments, we immediately attempted to incorporate these covariates into our statistical models. However, during assumption testing, we found that due to the inherently high variability of children's synergy data, several spatiotemporal parameters extracted in this study (e.g., Circular SD and Pearson's r) failed both the Shapiro-Wilk test for normality and Levene's test for homogeneity of variances. Under the circumstances of violating the core assumptions of parametric tests, forcefully applying a traditional ANCOVA could lead to severe bias in statistical inference (e.g., false positives or false negatives).

Driven by our commitment to data authenticity and statistical rigor, we had to retain the non-parametric Kruskal-Wallis test—which does not impose strict distributional assumptions—as the primary analytical method for the current study, and were unable to forcefully incorporate the covariates. To address your valid concerns and ensure the scientific objectivity of our manuscript, we have added a dedicated paragraph to the Limitations section. We not only explicitly acknowledge that failing to systematically control for these covariates is a major limitation, but we also explicitly state: "The existing statistical approach, which relies on conventional non-parametric tests (e.g., the Kruskal-Wallis test), cannot fully disentangle 'peripheral biomechanical constraints' from 'central neurodevelopmental effects' ." Furthermore, we propose that "future research with expanded sample sizes should incorporate Generalized Linear Mixed Models (GLMMs) or non-parametric analysis of covariance (ANCOVA) frameworks to precisely adjust for these confounding factors."

We hope the reviewer understands this statistical compromise necessitated by the actual data distribution, and appreciates our candid and profound reflection on this matter within the Limitations section.

Revision Location：

1.Discussion (Limitations): Added a new paragraph detailing the limitation of uncontrolled key covariates, and outlining future solutions using GLMMs and non-parametric ANCOVA frameworks.

Comment 4 (Major Weakness 4: Permissive VAF Threshold)

Comment:

The variance accounted for (VAF) threshold of 85% is comparatively low for concatenated non-negative matrix factorization (cNMF) in locomotion studies, which typically employ a 90% or higher threshold. This permissive cutoff risks overfitting and extracting non-physiological movement noise as valid "sporadic" synergies.

Response：

We sincerely thank the reviewer for this highly constructive and professional feedback. We fully agree with your assessment that a VAF threshold of 85% is relatively lax in the context of motor synergy research, which carries the risk of overfitting and capturing non-physiological noise.

To ensure the physiological validity of the extracted synergy modules and the rigor of our data reconstruction, we have fundamentally adjusted our extraction criteria in the revised manuscript:

1.Raised the VAF threshold: We re-ran the entire algorithmic pipeline, strictly increasing the global VAF threshold for determining the optimal number of synergies (N) from 85% to 90%. This adjustment allows our model to strike a better balance between reconstruction accuracy and model complexity, effectively filtering out mathematical noise.

2.Supplemented methodological literature support: To confirm the applicability and authority of this threshold in multi-limb movement studies (particularly hands-and-knees crawling), we have supplemented the Methods section with key literature support (Li C, Chen X, Zhang X, et al., Frontiers in Neuroscience, 2023), which explicitly identifies 90% as a widely accepted rigorous cutoff value in this field.

3.Comprehensive update of Results and Limitations: After applying the 90% threshold, the mean number of extracted modules was updated to 7.82 ± 1.05. This more refined modular partitioning accurately reflects the complexity of children's multi-degree-of-freedom control strategies. Furthermore, as this methodological shortcoming has been overcome, we have removed the previous statement regarding the lower VAF threshold from the "Limitations" section of the Discussion.

We are confident that this critical modification has significantly enhanced the reliability and scientific value of our study's conclusions.

Revision Location：•

1.Methods (Determination of the number of synergy modules [N] and data quality control): Explicitly stated that the VAF threshold has been raised to 90% and added the citation of Li C, et al. (2023) .

2.Results (Number of muscle synergies and Intra-individual spatiotemporal control Characteristics): Comprehensively updated the statistics for the number of extracted synergies and their distribution based on the VAF ≥ 90% criterion.

3.Discussion & Limitations: Removed the discussion concerning the lower 85% threshold.

Minor Weakness 1 (Arbitrary Muscle Dominance Threshold)

Comment:

The use of an arbitrary threshold (W > 0.5) to define dominant muscles within a module lacks a rigorous mathematical foundation.

Response：

We sincerely thank the reviewer for this rigorous methodological review. We fully agree that the criterion of W > 0.5 for defining dominant muscles in our original manuscript was arbitrary and lacked sufficient literature or mathematical support. To rectify this deficiency and ensure methodological rigor, we have replaced this arbitrary threshold in the revised manuscript with a standard supported by established literature in this specific field of research. Specifically, we have adjusted the normalized weight threshold for defining dominant (active) muscles to W > 0.3. This criterion is directly adapted from highly relevant, peer-reviewed literature (Xiong QL, Wu XY, Yao J, et al., Frontiers in Neurology, 2018). That study utilized synchronized sEMG and kinematic analysis to investigate inter-limb muscle synergies in children during hands-and-knees crawling and explicitly stated: "Specifically, muscles in a synergy were defined as active if their normalized weight values exceeded 0.3". Adopting this threshold, which has been strictly validated within the field, not only provides a solid methodological foundation for our muscle function classification but also enhances the comparability of our study with other research on crawling motor control. Based on this updated W > 0.3 criterion, we have comprehensively updated the description in the Methods section, the threshold reference line in Fig 3, and the list of dominant muscles for each synergy module in Table 2.

Revision Location：•

1.Methods (Cross-individual module clustering and quantification of spatiotemporal characteristics): Explicitly stated that the threshold has been updated to 0.3 and supplemented with the key reference (Xiong et al., 2018).

2.Results (Fig 3 caption & Table 2): The reference line for Fig 3 has been updated to W=0.3; the title of Table 2 and the dominant muscles listed within have been comprehensively updated according to the new threshold.

Minor Weakness 2 (Data Interpolation Risks)

Comment:

The uniform time-normalization to 100 points via PCHIP interpolation removes temporal variations in stance/swing phase ratios across the different age groups, potentially masking crucial developmental differences in motor control timing.

Response：

We sincerely thank the reviewer for their keen insight and professional guidance regarding the details of our data processing. We fully agree with your perspective that uniformly time-normalizing each complete crawling cycle to 100 data points eliminates the absolute time dimension and may potentially "obscure potential developmental differences" in the stance-to-swing phase ratios across age groups.

Because the primary objective of this study was to explore the spatial structures and reorganization patterns of "global muscle synergy patterns," we did not explicitly partition the crawling cycles into distinct stance and swing phases for separate normalization during the initial study design. However, the issue you raised is critical; this global normalization strategy indeed constitutes a methodological compromise that limits a deeper investigation into fine-grained temporal control characteristics.

To ensure the objectivity and rigor of the manuscript, we have fully adopted your suggestion by adding a dedicated paragraph in the "Limitations" section (the third limitation) of the revised manuscript. In this section, we explicitly acknowledge that the current global time-normalization approach may "obscure potential developmental differences in the stance-to-swing temporal ratio," and we formally propose that future research should combine comprehensive spatiotemporal kinematic data with "phase-specific" normalization strategies to more precisely elucidate these temporal characteristics.

Revision Location：

1.Discussion (Limitations): Added a third limitation paragraph, which provides a comprehensive and candid discussion regarding the potential risks associated with global time interpolation and outlines directions for future improvement.

Specific Comment 1 (Page 2, Line 46)

Comment:

The claim that the distal fine-tuning synergy module (M6) was absent (0.0% detection rate) in the 66–71-month age group but re-emerged in the 72–77-month group relies on extremely small and unequal sub-samples after data exclusion. Address the high probability that this is a statistical artifact rather than a genuine physiological phenomenon.

Response：

We sincerely thank the reviewer for their exceptionally rigorous scientific attitude. We fully agree with your assessment: in our original manuscript, due to the high data exclusion rate (up to 40% in the original G5 group), the conclusion regarding the "re-emergence" of the M6 module was indeed severely lacking in statistical persuasiveness and was likely a statistical artifact resulting from small sample size and data attrition.

As detailed in our response to "Major Weakness 2," to fundamentally eliminate the risk of such statistical artifacts, we comprehensively optimized our data preprocessing and strictly increased the VAF threshold to 90% for re-extracting synergy modules. Ultimately, we successfully retained 49 out of 50 participants (a retention rate of 98%), and the G4 and G5 groups—which you specifically focused on—now demonstrate exceptionally high data integrity (G4 group n=9, G5 group n=10).

With the "small sample artifact" defect eliminated, we tracked the re-extracted 12 group-level muscle synergies across age groups. Interestingly, the results show that even with a sufficient and well-balanced sample size, certain specific synergies responsible for distal fine-tuning (e.g., the M9 module, which dropped to 0% in the G4 group and partially recovered in the G5 group) still exhibited significant fluctuations in population prevalence.

Supported by robust statistical power, we are confident that these observations are not statistical artifacts caused by data attrition. In the revised manuscript, we have re-characterized and deeply discussed this phenomenon: we interpret this fluctuation as a "nonlinear developmental reorganization" experienced by children around 5.5 years of age. This reflects a dynamic trial-and-error process in which the developing CNS, lacking precise feedforward internal models, actively adjusts its strategy for "freeing" and "freezing" distal joints, rather than being a mere system error.

We have thoroughly rewritten the relevant paragraphs in the Results and Discussion sections to ensure that all conclusions are strictly based on the latest and reliable data foundation.

Revision Location：

1.Results (Age-related differences in muscle synergy control strategies): Detailed the true nonlinear fluctuation data of specific modules (e.g., M2, M9, M10) across age groups based on the new sample of 49 participants.

2.Discussion: Removed previous interpretations derived from limited sample size and added a rigorous discussion regarding the G4 stage as a "critical window for motor control pattern reorganization".

Specific Comment 2 (Page 3, Line 75)

Comment:

The introduction frames the crawling movement as a model for exploring how the central nervous system coordinates degrees of freedom, but fails to contextualize recent overarching advancements in multi-limb sports biomechanics and multi-joint motor rehabilitation modeling. To improve the theoretical framing of this research, incorporate perspectives from [XXX Ben Ezzdine L, Dhahbi W, Chèze L: Editorial: Advancing biomechanics: enhancing sports performance, mitigating injury risks, and optimizing athlete rehabilitation, volume II. Frontiers in Sports and Active Living 2026, Volume 8 - 2026. XXX].

Response：

We sincerely thank the reviewer for this forward-looking and highly relevant literature suggestion. We fully agree with your viewpoint: situating the multi-degree-of-freedom coordination issue of children's hands-and-knees crawling within the broader context of current multi-limb biomechanics and multi-joint rehabilitation modeling significantly broadens the scope of this study and enhances its theoretical value and application breadth.

We have fully adopted your suggestion by supplementing the corresponding background discussion in the Introduction section of the revised manuscript and formally integrating the perspective of your recommended literature. This addition not only greatly enriches the theoretical framework of this study but also demonstrates to the reader the bridge between motor control mechanisms in basic developmental science and their applications in sports science and rehabilitation medicine.

Revision Location：

1.Introduction: Added a new sentence linking the core research question to the frontier advances in multi-limb biomechanics and rehabilitation modeling.

2.References: The corresponding recommended literature has been formally added to the reference list (Reference [15]).

Specific Comment 3 (Page 5, Line 129)

Comment:

The initial sample of 10 participants per subgroup is compromised following the exclusion of 7 subjects. Provide the exact final sample size (n) and sex distribution for each of the five age groups to clarify the statistical power.

Response：

We sincerely thank the reviewer for this suggestion. We fully understand that accurate sample sizes (n) and gender distributions are crucial for demonstrating statistical power. As previously mentioned, by optimizing our data preprocessing pipeline, we successfully increased the overall effective sample size to 49 participants. We have supplemented a table in the "Subjects" section of the revised manuscript, which explicitly lists the exact sample size and gender distribution for each of the five age groups after excluding individual participants with artifacts, allowing the reviewer to confirm the robustness of our statistical structure. As shown in the table 1, with the exception of the 66–71 months age group, where n = 9 due to the exclusion of one participant, all other groups maintained balanced sample sizes and gender distributions. This highly balanced sample structure ensures the statistical power of our assessment of age main effects.

Revision Location：

1.Methods (Subjects): Added a detailed table 1 of the aforementioned sample sizes and gender distributions to clarify the statistical power.

Specific Comment 4 (Page 7, Line 163)

Comment:

The 16-channel sEMG setup monitors large superficial muscles but omits deep core stabilizers (e.g., transversus abdominis, multifidus) and shoulder girdle stabilizers (e.g., serratus anterior), which are critical for quadrupedal stability. Acknowledge this limitation in the discussion.

Response：

We sincerely thank the reviewer for their meticulous and professional reminder. We fully agree with your perspective: in hands-and-knees crawling, deep core stabilizing muscles—such as the transversus abdominis and multifidus—as well as shoulder girdle stabilizers, such as the serratus anterior, play a vital and foundational role in maintaining trunk posture and global dynamic stability.

Due to the inherent constraints of surface electromyography (sEMG) technology, our 16-channel setup could only non-invasively monitor major superficial motor muscles; this indeed resulted in our current analysis failing to capture the activation characteristics of deep stabilizing muscles. This implies that the muscle synergy patterns we extracted may not encompass the complete repertoire of neuromotor control strategies employed by the central nervous system (CNS) during children's crawling.

We have fully adopted your suggestion by adding a dedicated paragraph in the "Limitations" section (the fourth limitation) of the revised manuscript. In this paragraph, we explicitly acknowledge that failing to include the aforementioned deep core and shoulder girdle stabilizing muscles is a major limitation of this study, and we detail the potential impact of this limitation on a comprehensive analysis of the neuromechanical mechanisms of crawling.

Revision Location：

1.Discussion (Limitations): Added a new limitation statement detailing the constraint of only monitoring superficial muscles while omitting deep stabilizing muscles, as well as the associated implications.

Specific Comment 5 (Page 8, Line 187)

Comment:

The adaptive cycle segmentation algorithm relies on kinematic velocity thresholds to differentiate true motor rhythms from transient impacts and skin movement artifacts. This multivariate signal processing approach lacks a strong justification regarding how genuine biological cycles are differentiated from non-physiological temporal noise. Reinforce the signal processing logic by referencing similar multivariate analytical frameworks designed to isolate true biological rhythms from temporal artifacts, such as [XXX Dhahbi W, Dergaa I: Machine learning with temporal control designs for testing rhythmic specificity in chronobiology: A multivariate framework proposal for distinguishing genuine biological rhythms from temporal artifacts. Chronobiology International 2026:1-8. XXX].

Response：

We sincerely thank the reviewer for their rigorous scrutiny of our signal processing details and for the valuable literature recommendation. We fully agree with your perspective: in complex kinematic data, merely describing the operation of an adaptive speed threshold is insufficient. It is essential to have a robust theoretical framework to support the validity of distinguishing "authentic motor rhythms" from "non-physiological temporal noise."

To strengthen our signal processing rationale and establish a more solid methodological foundation, we have fully adopted your suggestion. In the "Methods: Segmentation of Cycles" section, we have introduced the theoretical basis of the multivariate temporal control framework. Specifically, before detailing the adaptive algorithm, we added the following core argument: "To effectively isolate authentic motor rhythms from non-physiological temporal noise in complex kinematic data, our signal processing rationale draws upon multivariate temporal control frameworks used for distinguishing biological rhythms from temporal artifacts [39]." The introduction of this theoretical framework perfectly explains the dynamic percentile thresholds and the debouncing time window techniques we employed; these were implemented not just for the sake of "segmentation," but to filter out temporal artifacts caused by transient impacts and skin motion at the algorithmic level.

We have formally added your highly valuable recommended literature (Dhahbi & Dergaa, 2026) to our reference list. This addition significantly enhances the scientific rigor of our data extraction process.

Revision Location：•

1.Methods (Segmentation of Cycles): Added a theoretical justification for the signal processing rationale, clarifying the methodological basis for distinguishing authentic motor rhythms from temporal artifacts.

2. References: Formally cited the recommended literature (Reference [39]).

Specific Comment 6 (Page 8, Line 194)

Comment:

Applying a 40–450 Hz band-pass filter to the pectoralis major and latissimus dorsi to suppress ECG artifacts risks attenuating genuine low-frequency motor unit action potentials. Justify this frequency band selection with supporting methodological literature.

Response：

We sincerely thank the reviewer for this highly professional correction. We fully agree with your point: setting the cutoff frequency of the high-pass filter to 40 Hz to suppress ECG artifacts is indeed overly aggressive and carries a substantial risk of excessively attenuating authentic low-frequency motor unit action potentials (MUAP) in the pectoralis major and latissimus dorsi.

After carefully evaluating the trade-off between suppressing ECG artifacts and preserving authentic physiological signals, we concluded that the previous strategy of implementing differential band-pass filtering for different muscle groups was methodologically inadequate. To fundamentally mitigate the risk of losing low-frequency physiological signals and to ensure the standardization of the data processing pipeline across all channels, we have completely abandoned the differential filtering strategy in our revised manuscript.

We have now applied a standardized 10–400 Hz band-pass filter to all sEMG signals across all channels. This broader and unified frequency band selection is widely recognized in the fields of surface electromyography and muscle synergy analysis; it maximizes the preservation of authentic low-frequency motor unit action potentials while meeting the data quality requirements of this study. We have comprehensively updated the description of data preprocessing in the Methods section of our revised manuscript.

Revision Location：

1.Methods (Data Preprocessing): Removed all descriptions regarding the 40–450 Hz filter and the differential filtering strategy for specific muscles; explicitly stated that a unified 10–400 Hz band-pass filter was applied to all recorded channels.

Specific Comment 7 (Page 9, Line 202)

Comment:

Time-normalizing each cycle to 100 equidistant points eliminates absolute cycle duration and relative phase timing differences (stance vs. swing ratio) between children of different ages. Clarify how this temporal distortion impacts the evaluation of temporal phase dispersion (Circular SD).

Response：

We sincerely thank the reviewer for raising this profound and professional question. We fully agree with your perspective: uniformly time-normalizing each complete cycle to 100 equidistant points inevitably eliminates the absolute time dimension and may obscure the differences in stance-to-swing phase ratios.

Regarding how such temporal distortion affects the assessment of temporal phase dispersion (Circular SD), we must clarify that because we did not explicitly partition crawling cycles into distinct stance and swing phases prior to interpolation, the Circular SD we calculated measures the relative concentration or dispersion of muscle activation within the standardized mathematical cycle (0%–100%). This means that if there are subtle developmental changes in the stance-to-swing phase ratios across different age groups, such variations would be "smoothed out" during the global interpolation process. Consequently, our Circular SD metric might underestimate the true, physiologically-based temporal phase dispersion.

To transparently communicate this methodological impact to the reader, we have explored the potential masking effect of our global time-normalization strategy on the assessment of temporal characteristics in the "Limitations" section of the revised manuscript. We explicitly recommend that future studies should combine comprehensive spatiotemporal kinematic data with "phase-specific" normalization strategies to more precisely elucidate these temporal characteristics.

Revision Location：

1.Discussion (Limitations): Added a limitation paragraph explicitly discussing how the global normalization strategy—without partitioning stance/swing phases—may mask potential developmental differences (such as temporal ratios) and limit the assessment of temporal characteristics.

Specific Comment 8 (Page 9, Line 210)

Comment:

The application of concatenated non-negative matrix factorization (cNMF) for dimensionality reduction of sEMG matrix data proceeds without discussing the broader methodological necessity to standardize analytical workflows for advanced sensor technologies and algorithmic data extractions in motor behavior. To establish a firmer methodological baseline for algorithmic signal processing in sports science, integrate the framework outlined by [XXX Dhahbi W, Chamari K: The Algorithmic Athlete: A Call to Standardize Assessment of Sensor Technologies and Artificial Intelligence. International Journal of Sports Physiology and Performance 2026, 1(aop):1-2. XXX].

Response：

We sincerely thank the reviewer for this highly constructive and forward-looking suggestion. We fully concur with your view: as advanced sensor technologies and complex computational algorithms are widely applied in motor behavior research, establishing a macroscopic methodological baseline for standardized data extraction and signal processing pipelines is essential before delving into specific algorithms like cNMF. This is not only a matter of scientific rigor but also crucial for the reproducibility of our results. To build a more robust methodological foundation, we have fully adopted your suggestion by supplementing a discussion on the necessity of algorithmic standardization at the beginning of the "Methods: Muscle Synergy Extraction and Non-Negative Matrix Factorization" section. Specifically, we have introduced the following statement: "With the widespread application of advanced sensor technologies and computational algorithms in motor behavior research, establishing a standardized data extraction and signal processing pipeline has become an essential prerequisite for ensuring scientific rigor and reproducibility. In response to this methodological initiative and to ensure the robustness of the sEMG data analysis..." By including this discussion, we have closely integrated our specific dimensionality reduction operations with broader calls for methodological standardization in the field.

Furthermore, we have formally added your recommended literature (Dhahbi W, Chamari K., International Journal of Sports Physiology and Performance, 2026) as key methodological support in our reference list (Reference [45]).

Revision Location：

1.Methods (Muscle Synergy Extraction and Non-Negative Matrix Factorization): Added a discussion at the beginning of the section regarding the necessity of standardized analytical pipelines for sensor technology and algorithmic data extraction, serving as the theoretical prerequisite for applying the cNMF algorithm.

2. References: Formally added the recommended literature.

Specific Comment 9 (Page 10, Line 231)

Comment:

The VAF threshold of 85% is permissive. Recalculate or justify the module extraction using a more rigorous methodology, such as plotting the VAF curve and identifying the point of inflection, to ensure M5 and M6 are not simply mathematical noise.

Response：

We sincerely thank the reviewer for their rigorous scientific scrutiny. As noted in our response to "Major Weakness 4," we have recognized that a VAF threshold of 85% carries a significant risk of extracting non-physiological mathematical noise. Therefore, we fully understand your valid concerns regarding whether the M5 and M6 modules extracted under the 85% threshold in the original manuscript were indeed "mathematical noise."

To fundamentally resolve this concern, we chose not to justify the 85% threshold but rather fully adopted your suggestion by re-calculating the entire dataset using more rigorous methodological standards. Specifically, we implemented the following strict re-calculation steps in the revised manuscript:

1.Raised the VAF threshold: We strictly increased the global VAF threshold for determining the optimal number of synergies (N) from 85% to 90%.

2.Strengthened the algorithm: Considering that the NMF algorithm is susceptible to local minima, we executed 20 independent runs with random initialization for each candidate number of synergies (N), set the maximum number of iterations to 5,000, and retained the model with the lowest reconstruction error.

Through this rigorous re-calculation process, the original 6-synergy structure (containing the former M5 and M6) has been replaced by a new set of 12 group-level muscle synergies with higher reconstruction accuracy. The new synergy modules (e.g., M1–M3, M8–M10) were extracted under strict conditions (VAF ≥ 90% and avoidance of local optima), ensuring that they represent authentic physiological control strategies rather than mathematical noise. (Note: For literature support regarding the VAF threshold increase, please see our detailed response to Major Weakness 4.)

Revision Location：

1.Methods (Determination of the number of synergy modules [N] and data quality control): Detailed the strict re-calculation process, including the 20 independent runs, 5,000 iterations, and the 90% threshold.

2.Results: Comprehensively updated the number of synergy modules and clustering results obtained under the rigorous re-calculation standards.

Specific Comment 10 (Page 11, Line 248)

Comment:

The selection of W > 0.5 as the threshold for dominant muscle identification is arbitrary. Implement a statistical approach, such as bootstrapping or permutation testing, to establish the significance of muscle weights within each module.

Response：

We sincerely thank the reviewer for their strict requirements regarding the scientific validity of our threshold settings. As detailed in our response to "Minor Weakness 1," we fully agree that the W>0.5 criterion set in the original manuscript was an arbitrary threshold lacking sufficient justification.

Regarding your suggestion to use bootstrapping or permutation tests, after careful evaluation, we selected an alternative, standardized approach that is more widely recognized in this specific field and facilitates cross-study comparisons, thereby eliminating the arbitrariness of the threshold. We directly adopted the normalized weight threshold established in highly relevant authoritative peer-reviewed literature (Xiong QL, Wu XY, Yao J, et al., Frontiers in Neurology, 2018). Specifically, we have strictly updated the standard for defining dominant (active) muscles to W>0.3. This objective cutoff value, supported by authoritative literature, not only provides a solid methodological basis for classifying muscle dominance—perfectly resolving the issue of "arbitrariness"—but also ensures the consistency and comparability of this study with other research on children's crawling synergies. We have comprehensively updated the Methods and relevant figures/tables based on this new standard.

Revision Location：

1.Methods & Results (Fig 3, Table 2): Please refer to the response to Minor Weakness 1; these have been comprehensively updated to the W>0.3 threshold, supported by the key reference.

Specific Comment 11 (Page 12, Line 274)

Comment:

The Kruskal-Wallis test does not account for covariates. Implement a robust statistical framework (e.g., mixed-effects modeling or ANCOVA using R or SPSS) to isolate the main effect of chronological age while controlling for individual crawling speed and anthropometry.

Response：

We sincerely thank the reviewer for again emphasizing this critical statistical issue. This feedback is entirely consistent with the core concerns you raised in "Major Weakness 3." We fully agree with your suggestion that to precisely isolate the main effect of chronological age, it is essential to control for confounding factors such as crawling speed and individual anthropometric characteristics. However, as detailed in our response to "Major Weakness 3," because several spatiotemporal parameters extracted in this study failed tests for normality and homogeneity of variances, the forceful application of traditional parametric tests (such as conventional ANCOVA) posed significant statistical risks. Out of a commitment to data integrity, we retained the non-parametric Kruskal-Wallis test as our primary analytical method. To compensate for this statistical compromise and maintain the highest level of academic transparency, we have added this issue as a core limitation in the "Limitations" section of the revised manuscript. In this new discussion, we explicitly acknowledge that our current statistical approach fails to systematically control for key covariates, thereby precluding a complete disentanglement of "peripheral biomechanical constraints" from "central neurodevelopmental effects." Furthermore, we have fully adopted your constructive approach by explicitly proposing that future research should incorporate Generalized Linear Mixed Models (GLMMs) or non-parametric analysis of covariance (ANCOVA) frameworks to precisely address this issue. For specific details and methodological considerations, please refer to our comprehensive response to "Major Weakness 3."

Revision Location：

1.Discussion (Limitations): As per our response to "Major Weakness 3," we have added a paragraph thoroughly discussing the limitation of uncontrolled covariates and providing an outlook on future statistical models (GLMMs/non-parametric ANCOVA).

Specific Comment 12 (Page 17, Line 381)

Comment:

The 40% data exclusion rate in the G5 group drastically reduces the sub-sample to 6 individuals. State this limitation explicitly alongside the results, as it fundamentally undermines the reliability of the M6 re-emergence hypothesis.

Response：

We sincerely thank the reviewer for their rigorous oversight regarding data integrity and the reliability of our conclusions. As reported in our responses to "Major Weakness 2" and "Specific Comment 1," we have fundamentally resolved this issue in the revised manuscript. By optimizing our data preprocessing pipeline and strictly increasing the VAF extraction threshold to 90%, we achieved a final valid sample size of 49 participants. Notably, the G5 group (72–77 months), which you specifically focused on, currently maintains a 100% data retention rate (10/10 participants fully retained), with only one participant excluded from the G4 group (66–71 months). Since the high exclusion rate of 40% in the G5 group from the original manuscript no longer exists, the "M6 re-emergence" hypothesis—which was driven by the small sample size—has been proven inaccurate. Consequently, we have completely removed the limitation statement regarding the high exclusion rate and have rewritten the Results section based on the complete, well-balanced new dataset of 49 participants. The new results demonstrate a more rigorous "nonlinear developmental reorganization" trajectory for specific synergy modules between the ages of 4 and 6 years. For detailed re-analysis and results, please refer to our comprehensive response to "Major Weakness 2."

Revision Location：

1.Methods & Results: As per our response to "Major Weakness 2," we have updated the retention rate (49/50) and removed all unreliable statements from the original manuscript that were based on the high attrition rate in the G5 group.

Specific Comment 13 (Page 18, Line 400)

Comment:

Assigning absolute kinetic functions such as "anti-gravity support" and "power source for lower-limb swing" is methodologically flawed without ground reaction force data. Soften these claims to reflect that they are inferences based on spatial muscle weights, not measured kinetic outputs.

Response：

We sincerely thank the reviewer for this rigorous correction. This feedback is entirely consistent with the core concerns raised in "Major Weakness 1." We fully agree that without synchronous ground reaction force (GRF) data for mechanical validation, assigning absolute kinetic functions such as "anti-gravity support" or "hindlimb swing power source" is methodologically flawed. To ensure our conclusions strictly align with the existing evidence base, we have comprehensively softened all absolute statements regarding biomechanical functions throughout the Abstract, Discussion, and Tables of the revised manuscript. Specifically, we now explicitly state that these biomechanical functions are "hypothesized," "postulated," or "inferred" based on spatial muscle weights. For example, we have updated the relevant statements to "are hypothesized to primarily contribute to generating elbow extension torque..." and "are likely associated with robust anti-gravity support." Furthermore, we have explicitly acknowledged in the "Limitations" section that the lack of kinetic parameters prevents the direct quantification of mechanical contributions. For detailed changes and specific locations, please refer to our comprehensive response to "Major Weakness 1."

Revision Location：

1.Abstract, Discussion, Tables, and Limitations: As per our response to "Major Weakness 1," all absolute descriptions of kinetic functions have been updated to speculative statements based on spatial weights.

Specific Comment 14 (Page 19, Line 416)

Comment:

The discussion concerning the sporadic synergy module M6 focuses on distal ankle joint adjustments and motor exploration in children, but the neuro-mechanical argument lacks comparison with broader developmental motor control paradigms that examine distal musculature control mechanisms and cross-over neuromuscular features in children relative to adults. Reference established developmental neuromuscular data regarding distal motor control pathways to support this interpretation, specifically referencing [XXX Othman AB, Dhahbi W, Bessifi M, Geant VA, Ursu VE, Behm DG, Chamari K, Chaouachi A: Mirror Visual Feedback Selectively Attenuates Crossover Fatigue in Distal Upper Limb Musculature: A Randomized Controlled Crossover Investigation Comparing Children and Adults. Life 2026, 16(3):435. XXX].

Response：

We sincerely thank the reviewer for providing this highly insightful developmental neuromechanical perspective and for the valuable literature recommendation. We fully agree with your viewpoint: comparing children's distal joint adjustment strategies with mature control mechanisms and crossed neuromuscular features in adults significantly deepens the theoretical rigor of our study.

As reported in our previous revisions (see Major Weakness 2 and Specific Comment 9), our data re-analysis using the more rigorous 90% VAF threshold led to a reorganization of the synergy modules, where the original M6 module was replaced by more refined specific modules responsible for distal fine-tuning (e.g., M1, M2, and M10). Although the module numbering has been reorganized, the core mechanism regarding "distal muscle control and fine-tuning" remains a key finding of our study.

We have fully adopted your suggestion and introduced this comparative perspective into the "Discussion" section of the revised manuscript. When explaining why the new distal modules (M1, M2, and M10) exhibit exceptionally high temporal phase variability, we explicitly added the following statement: "Recent comparative studies investigating distal muscle control mechanisms and crossed neuromuscular features in children versus adults have confirmed that distal motor pathways and inter-limb neural interactions during development possess plasticity and regulatory characteristics fundamentally distinct from those of adults." We interpret this high variability as an active sensorimotor exploration strategy unique to the developing CNS, rather than mere system noise.

Furthermore, we have formally added the important literature you recommended (Othman et al., 2026) to our reference list as a core evidentiary support for this neuromechanical argument.

Revision Location：

1.Discussion: In the paragraph discussing the high temporal variability of specific synergy modules (M1, M2, M10), we have added a theoretical elaboration comparing distal muscle control mechanisms and crossed neuromuscular features in children versus adults.

2.References: The literature by Othman et al. (2026) has been formally added to the reference list.

-----------------------------------------------------------------------------------------------------------------

Part 3: Response to Reviewer 2

First Issue: Interpretation of Spatial and Temporal Consistency

Comment:

The first hypothesis was that "during hands-and-knees crawling, the shared synergies that primarily govern proximal limbs will demonstrate spatial stability and temporal phase consistency within individuals, across individuals, and across age groups" (Lines 117-119).

Temporal:

The reported temporal pattern similarity r = 0.20 indicate essentially no meaningful temporal consistency. This consideration would affect all the subsequent claims and analyses based on temporal phase consistency.

Spatial:

Then, in Discussion (Lines 389-391) the authors claim that, based on their results, "The CNS relies on spatially stable shared synergies to achieve anti-gravity support and propulsion, maintaining basic mechanical stability"However, this affirmation is questionable. Which results would support it?

At intra-individual level, the reported mean intra-individual cosine similarity of 0.61 (line 300) falls below the commonly accepted threshold of 0.70 - 0.80 for spatial consistency. This raises questions about the robustness of the extracted synergy weight vectors. See for instance Sheng, Y., et al. (2024). IEEE Transactions on Neural Systems and Rehabilitation Engineering.

Also at cross-individual level the reported Cs is again below the acceptance threshold (Table 2).

Both the muscle weight vectors and the temporal activation patterns are poorly reproducible across cycles within the same subject. This undermines the validity of the synergy extraction.

Response：

We sincerely thank the reviewer for their keen insight and professional guidance. We fully accept your critique. To ensure that our claims strictly align with the objective data, we have comprehensively revised the core logic of our manuscript:

1.Objective interpretation of spatiotemporal consistency: We acknowledge that by adult standards (where CS typically ranges from 0.70 to 0.80), our reported intra-individual similarity scores (Mean CS = 0.58, Pearson's r = 0.16) do not reflect "high spatial stability and temporal phase consistency." The use of such absolute descriptors in the original manuscript was inaccurate. From a developmental neuromechanical perspective, this relatively low reproducibility is an authentic reflection of the immature neuromuscular control in 4- to 6-year-old children during complex quadrupedal tasks, characterized by high intra- and inter-individual motor variability. Consequently, we have reconstructed our hypothesis in the Introduction: "Within individuals across consecutive crawling cycles, these synergies will maintain relatively stable spatial structures despite temporal activation variability." We have also explicitly discussed in the revised manuscript that this low temporal similarity does not imply ineffective synergy extraction; rather, it reflects a "continuous fine-tuning" process through online sensory feedback as the CNS actively explores strategies to adapt to complex mechanical constraints.

2.Refinement of functional assertions: We agree that in the absence of synchronous ground reaction force (GRF) data, asserting absolute kinetic functions (e.g., "anti-gravity support" or "propulsion") is methodologically flawed. We have softened these absolute assertions throughout the revised manuscript:Softened inferential tone: All absolute functional claims have been reframed as "hypothesized biomechanical functions" inferred from spatial anatomical weights. We now state that these synergies are "hypothesized to contribute to" robust anti-gravity support and propulsion, rather than representing measured kinetic outputs; Structural robustness as the basis: Despite the temporal activation variability across individuals, the 12 group-level modules identified via the Gap Statistic demonstrate consistent spatial weight vectors (W > 0.3). This "structural robustness" serves as the foundational blueprint for postural control during crawling; Limitation statement: We have added a dedicated declaration in the "Limitations" section, acknowledging that the lack of kinetic parameters prevents the direct quantification of mechanical contributions and limiting our descriptions to "biomechanical inferences based on spatial weight distributions."

These revisions transition our findings from controversial "conclusions" to scientifically grounded "inferences" based on a rigorous developmental neuromechanical perspective—namely, that high motor variability is a signature of active sensorimotor exploration in developing children.

Revision Location：

1.Introduction: Revised Hypothesis 1 to discard the absolute assertion of "consistency."

2.Discussion: Added a paragraph detailing the physiological significance of low reproducibility (low r values and moderate CS values) in children's motor control exploration.

3.Conclusions: Comprehensively updated to objectively present the data (CS = 0.58, Circular SD = 16.73%) and clarify the "hypothesized" nature of the biomechanical functions.

Second Issue：Contradiction in Synergy Number N)

Comment:

Line 233-234: The authors claim that "only participants with N﹥4 were retained for subsequent statistical analyses", being N the number of synergies. However in Results (line 297) they affirm that N was equal to 4 in 8 participants.

Please explain.

Line 245: it seems that K-means clustering was performed forcing N = 6 synergies.

Please, justify this choice.

Figure 3: The large SD areas in the temporal patterns are clearly related to the temporal inconsistency already present at intra-individual level.

Conclusions:

Line 477-479: that's hardly supported by your data.

Response：

We sincerely apologize for the discrepancies between our methodological descriptions and the reported results in the original manuscript, as well as for any confusion caused by the previously forced clustering approach. In response to the core issues raised, we have taken the following actions:

1. Clarification regarding the screening criteria (N ≥ 4) and reported discrepancies We admit that the description regarding the "retention of participants with N ≥ 4" in our original manuscript was an oversight. As you pointed out, this led to a clear contradiction between the Methods and Results sections. To fundamentally rectify this and eliminate researcher-introduced bias, we have adopted a data-driven re-calculation strategy: we have completely abandoned the subjective truncation criteria based on the number of modules and instead adopted the variance accounted for (VAF ≥ 90%) as the sole quality control standard. In our updated analysis, the synergy numbers for the 49 participants naturally range from 5 to 10 (mean N = 7.82 ± 1.05), rendering the controversial "screening rules" obsolete and resolving the previous data discrepancies.

2. Abandonment of forced clustering and introduction of Gap Statistic We fully agree with your critique regarding the forced K=6 clustering. We have completely abandoned this approach. Prior to performing K-means clustering on the cross-individual spatial weight vectors, we introduced the Gap Statistic algorithm. Through this objective mathematical evaluation method, we determined the optimal number of clusters to be 12 and extracted 12 group-level muscle synergies (M1–M12) accordingly. This change ensures that the clustering results are entirely determined by the data itself, rather than by researcher-defined presets.

3. Explanation of high SD regions in Fig 3 The regions of high temporal activation variability (high SD) shown in Fig 3 represent what we emphasize as "intra-individual temporal activation variability". Re-interpretation of biological significance: In conjunction with our revised hypotheses, these high SD regions are not experimental measurement errors; rather, they reflect the authentic process of active, "feedback-driven fine-tuning" employed by 4–6-year-old children during complex crawling tasks; Control logic of spatiotemporal separation: Despite significant fluctuations in temporal phase (high SD), the extracted synergy modules maintain relative robustness in spatial weight distribution (mean CS = 0.58). This pattern of a "stable spatial blueprint" combined with "flexible temporal modulation" constitutes the core characteristic of postural control strategies in children and serves as a developmental hallmark distinguishing them from the steady-state gait observed in adults.

4. Revision of Conclusions We fully accept your critique. The statement regarding "absolute spatiotemporal consistency" in the original conclusion exceeded the scope supported by our objective data. Combined with your valuable feedback and the comprehensive data re-calculation using the 90% VAF threshold and the Gap Statistic, we have thoroughly rewritten the Conclusions. The new conclusions avoid exaggerated assertions, are strictly limited to the scope supported by current data, objectively state the coexistence of "stable spatial structures" and "considerable temporal variability" in children's hands-and-knees crawling, and rigorously attribute the fluctuations of specific modules to "nonlinear developmental reorganization".

Revision Location：•

1.Methods: Corrected the screening oversight regarding synergy numbers and deleted the subjective truncation criteria; detailed the steps for using the Gap Statistic to determine the number of clusters (K=12).

2.Results: Comprehensively updated the distribution of synergy numbers (N) and the 12 group-level muscle synergy modules obtained via objective clustering.

3.Discussion & Fig 3 Legend: Refined the explanation of the high SD regions in Fig 3, arguing that they represent motor exploration behaviors unique to developing children, and contrasted them with the relative robustness of the spatial structures.

4.Conclusions: Completely rewritten to ensure that all conclusions are strictly based on the latest re-calculated data foundation.

Minor issues: Fig. 1 and 5 are not available (The fig. 1 link actually downloads fig. 2, the 5 to fig. 3)

Response：

We sincerely apologize for the file linking and download errors that occurred during our initial submission. We have re-uploaded all image files to the journal's online submission system and meticulously verified every entry. We have confirmed that the links for Figure 1, Figure 5, and all other figures are now correct and accessible, ensuring they can be accurately downloaded and reviewed.

Thank you once again for the time and effort you have dedicated to improving the quality of our manuscript.

Revision Location：

1.Submission System: All image files have been re-uploaded, and the corresponding links have been corrected and verified.

Attachment Submitted filename: Response to Reviewers.docx

# Peer review: Decision Letter 1

**DOI:** 10.1371/journal.pone.0358587.r003

**Authors:** Mário Espada

13 Aug 2026

Muscle Synergy Characteristics during Hands-and-Knees Crawling in Children Aged 4–6 Years

PLOS One

Dear Dr. Jia,

Thank you for submitting your manuscript to PLOS ONE. After careful consideration, we feel that it has merit but does not fully meet PLOS ONE’s publication criteria as it currently stands. Therefore, we invite you to submit a revised version of the manuscript that addresses the points raised during the review process.

Please submit your revised manuscript by Sep 24 2026 11:59PM. If you will need more time than this to complete your revisions, please reply to this message or contact the journal office at plosone@plos.org. When you're ready to submit your revision, log on to https://www.editorialmanager.com/pone/ and select the 'Submissions Needing Revision' folder to locate your manuscript file.

Please include the following items when submitting your revised manuscript:

- A letter that responds to each point raised by the academic editor and reviewer(s). You should upload this letter as a separate file labeled 'Response to Reviewers'.
- A marked-up copy of your manuscript that highlights changes made to the original version. You should upload this as a separate file labeled 'Revised Manuscript with Track Changes'.
- An unmarked version of your revised paper without tracked changes. You should upload this as a separate file labeled 'Manuscript'.

If you would like to make changes to your financial disclosure, please include your updated statement in your cover letter. Guidelines for resubmitting your figure files are available below the reviewer comments at the end of this letter.

If applicable, we recommend that you deposit your laboratory protocols in protocols.io to enhance the reproducibility of your results. Protocols.io assigns your protocol its own identifier (DOI) so that it can be cited independently in the future. For instructions see: https://journals.plos.org/plosone/s/submission-guidelines#loc-laboratory-protocols. Additionally, PLOS ONE offers an option for publishing peer-reviewed Lab Protocol articles, which describe protocols hosted on protocols.io. Read more information on sharing protocols at https://plos.org/protocols?utm_medium=editorial-email&utm_source=authorletters&utm_campaign=protocols.

As the corresponding author, your ORCID iD is verified in the submission system and will appear in the published article. PLOS supports the use of ORCID, and we encourage all coauthors to register for an ORCID iD and use it as well. Please encourage your coauthors to verify their ORCID iD within the submission system before final acceptance, as unverified ORCID iDs will not appear in the published article. *Only*  the individual author can complete the verification step; PLOS staff *cannot*  verify ORCID iDs on behalf of authors.

We look forward to receiving your revised manuscript.

Kind regards,

Mário Espada, PhD

Academic Editor

PLOS One

Journal Requirements:

If the reviewer comments include a recommendation to cite specific previously published works, please review and evaluate these publications to determine whether they are relevant and should be cited. There is no requirement to cite these works unless the editor has indicated otherwise.

Please review your reference list to ensure that it is complete and correct. If you have cited papers that have been retracted, please include the rationale for doing so in the manuscript text, or remove these references and replace them with relevant current references. Any changes to the reference list should be mentioned in the rebuttal letter that accompanies your revised manuscript. If you need to cite a retracted article, indicate the article’s retracted status in the References list and also include a citation and full reference for the retraction notice.

Additional Editor Comments:

Dear Authors,

Congratulations on your work.

Please revise the manuscript in light of the reviewers' minor suggestions.

Thank you.

Best regards.

[Note: HTML markup is below. Please do not edit.]

Reviewers' comments:

Reviewer's Responses to Questions

**Comments to the Author**

Reviewer #1: All comments have been addressed

Reviewer #3: (No Response)

Reviewer #4: All comments have been addressed

Reviewer #5: (No Response)

Reviewer #6: (No Response)

Reviewer #7: All comments have been addressed

**********

2. Is the manuscript technically sound, and do the data support the conclusions?

Reviewer #1: Yes

Reviewer #3: Partly

Reviewer #4: Yes

Reviewer #5: Yes

Reviewer #6: Yes

Reviewer #7: Yes

**********

3. Has the statistical analysis been performed appropriately and rigorously?

Reviewer #1: Yes

Reviewer #3: No

Reviewer #4: (No Response)

Reviewer #5: No

Reviewer #6: Yes

Reviewer #7: Yes

**********

4. Have the authors made all data underlying the findings in their manuscript fully available?

The PLOS Data policy

Reviewer #1: Yes

Reviewer #3: Yes

Reviewer #4: Yes

Reviewer #5: No

Reviewer #6: Yes

Reviewer #7: Yes

**********

5. Is the manuscript presented in an intelligible fashion and written in standard English?

Reviewer #1: Yes

Reviewer #3: Yes

Reviewer #4: Yes

Reviewer #5: Yes

Reviewer #6: Yes

Reviewer #7: Yes

**********

Reviewer #1: General Comments

The revised manuscript addresses the majority of the prior methodological concerns. The implementation of a more stringent variance accounted for threshold and the modification of the dominant muscle extraction criteria improve the analytical rigor of the study. The authors have adequately integrated the suggested literature and softened the inferential tone regarding biomechanical functions.

A major weakness persists regarding the interpretation of the dimensionality reduction. The updated methodology resulted in the extraction of twelve group-level synergies from sixteen recorded muscles. Extracting twelve modules from sixteen channels represents a highly limited dimensionality reduction. The physiological validity of identifying almost as many synergies as recorded muscles requires cautious interpretation, as this outcome might reflect mathematical fractionation of the electromyographic data rather than authentic neural modularity.

A minor weakness relates to the interpretation of motor variability. The authors correctly added a limitation regarding the inability to control for covariates such as crawling speed and anthropometrics using non-parametric statistical methods. The discussion attributes the high temporal phase dispersion to active sensorimotor exploration. The authors must acknowledge in the discussion that a portion of this temporal variability might directly stem from the uncontrolled variations in self-paced crawling speed across the distinct age groups.

Specific Comments

Page 2, Line 41. The abstract states a mean $N=7.82 \pm 1.05$ synergies recruited. State the total number of muscles recorded directly in this sentence to provide immediate context for the dimensionality reduction ratio.

Page 4, Line 106. The phrase "degrees of freedom redundancy (DOF redundancy)" contains unnecessary repetition. Revise this instance to "degree of freedom (DOF) redundancy" to improve readability.

Page 7, Line 235. The reported mean intra-individual spatial consistency is 0.58, and the temporal waveform similarity is 0.16. Clarify briefly in the results text that these values are low relative to adult steady-state locomotion, directing the reader to the developmental interpretation provided in the subsequent discussion section.

Page 9, Line 306. The extraction of an average of 7.82 synergies per individual and 12 at the group level from a 16-muscle configuration is unusually high. Address whether the non-negative matrix factorization algorithm is fragmenting single muscle activations into independent modules, and discuss this specific risk within the context of the updated 90% threshold.

Page 12, Line 446. The limitations section identifies the lack of covariate control using traditional parametric tests. Add a brief note explaining how variations in self-paced crawling speed might directly inflate the reported temporal phase dispersion values observed in the specific synergies.

Reviewer #3: Major & Minor Comments1. Statistical Modeling and Covariates Observation: The authors indicated in their response that they were unable to apply Analysis of Covariance (ANCOVA) or Mixed-Effects Models due to the non-fulfillment of normality and homogeneity of variance assumptions for some variables, relying strictly on the Kruskal-Wallis test.

Evaluation: While the authors' statistical rationale is understandable, the failure to control for variables such as "crawling speed" and "anthropometric characteristics" leaves a gap regarding whether the observed differences are purely age-related or stem from variations in individual motor performance.

Required Modification: It would be beneficial to attempt data transformation (such as log transformation) to test parametric models or utilize Generalized Linear Mixed Models (GLMMs) that handle non-normal data better, while sufficiently clarifying this limitation in the limitations section

2. Spatiotemporal Consistency Interpretation Observation: The average internal spatial consistency for individuals reached (CS = 0.58), which is relatively lower than the traditional consistency standards of (0.70 - 0.80) in mature automated movements. Evaluation: The authors successfully reframed this aspect in the discussion, considering this variability as a reflection of the "motor exploration" process in children rather than a measurement error. This interpretation must be emphasized with utmost care to prevent the muscle synergy from appearing so unstable that the study loses its reliability.

3. Lack of Kinetic Validation Observation: The reviewers praised the authors' responsiveness regarding the "absence of ground reaction f orce (GRF) data" and the modification of terms to "hypothesized functions". Required Modification:

It must be ensured that the tables (such as Table 2) and the text are completely free of any categorical phrasing indicating actual force generation, strictly adhering to probabilistic phrases such as ("it is hypothesized to contribute to...") as promised by the authors in their response.

III. Recommendation Recommendation: Conditional Acceptance with Major Revisions.

Justification: The manuscript holds high scientific value within the context of athletic hero engineering and the development of neuromotor control, and the researchers have responded to most previous critiques with clear methodological rigor. Only some phrasing adjustments and the discussion of statistical limitations with final precision remain to ensure robust publication in PLOS ONE.

Reviewer #4: (No Response)

Reviewer #5: Reviewer Report for PLOS ONE Manuscript ID: PONE-D-26-23547R1 Title: Muscle Synergy Characteristics during Hands-and-Knees Crawling in Children Aged 4–6 Years

General Comments The authors have invested considerable effort in this revision. I acknowledge and commend the following improvements: (1) the data retention rate was substantially improved (49/50, 98%); (2) the VAF threshold was raised from 85% to 90%; (3) forced K-means clustering (K=6) was replaced by a data-driven Gap Statistic approach yielding 12 modules; (4) differential band-pass filtering was abandoned in favor of a standardized 10–400 Hz filter; and (5) absolute biomechanical claims were systematically softened to speculative inferences. These changes address several of my previous concerns.

However, the revision introduces new methodological inconsistencies and, most critically, fails to resolve a fundamental ethical compliance issue. Below I outline remaining major and minor concerns.

Major Issues

1. Unresolved critical ethical timeline discrepancy (REQUIRES IMMEDIATE CLARIFICATION)

In the revised Methods, the authors state that participants were recruited between November 2024 and May 2025, yet data collection is still reported as occurring between June 15 and November 20, 2024. The ethics approval number (SXULL2024082) implies review in August 2024. This creates an impossible chronology: data collection began in June 2024, approximately five months before the stated recruitment period and two months before the implied ethics approval date.

Collecting human subjects data before institutional ethics approval is a serious violation of the Declaration of Helsinki. Simply changing "October" to "November" in the recruitment window does not resolve this contradiction. The authors must provide one of the following: (a) documented proof that the study received prospective ethical approval before June 15, 2024 (e.g., an approval letter with an earlier date); (b) a corrected, internally consistent timeline with verifiable dates; or (c) a formal waiver/retrospective approval letter from the Shanxi University Medical Ethics Committee. If none can be provided, this manuscript should be rejected on ethical grounds.

2. Internal inconsistency in the 12-module taxonomy

The new two-tier classification (shared vs. specific) applies dual criteria: prevalence >70% and coefficient of variation (CV) of prevalence across age groups <0.3. However, the classification appears inconsistently applied:

• M4 (77.55% prevalence, Circular SD = 35.10%) is labeled "shared," whereas M8 (73.47% prevalence, Circular SD = 21.36%) is labeled "specific." M4 exhibits higher temporal variability and lower spatial consistency than M8, yet is classified as a stable core module. If the CV criterion explains this, the actual CV values for every module must be explicitly reported.

• M6 (73.47% prevalence) is "shared," but its anatomical counterpart M8 (73.47% prevalence) is "specific." The manuscript does not justify why two modules with identical population coverage fall into different categories.

Furthermore, several "specific" modules show extremely low population coverage: M2 (30.61%), M9 (36.73%), and M12 (26.53%). With only 49 participants, a module detected in ~8–15 individuals risks being an algorithmic artifact of over-segmentation rather than a genuine physiological synergy. The authors should provide robustness validation (e.g., split-half reliability, bootstrap resampling) to prove these low-prevalence modules are reproducible.

3. The "prevalence" metric is undefined and mathematically inconsistent

In the Results, the authors report developmental trajectories using prevalence values such as:

• M2 in G1: 1.30%

• M2 in G4: 10.94%

• M9 in G4: 0%

• M9 in G5: 3.41%

If "prevalence" means the percentage of participants in a group exhibiting the module, these decimals are impossible. With n=10 in G1, the smallest non-zero prevalence is 10% (1/10). With n=9 in G4, the smallest non-zero prevalence is 11.11% (1/9). Values like 1.30% or 10.94% cannot represent integer participant counts. The authors must clarify the exact formula used to calculate these percentages. If they represent something other than binary occurrence (e.g., mean W weight, VAF contribution), the terminology must be changed, as "prevalence" and "detection rate" imply a categorical present/absent variable.

4. Over-interpretation of "nonlinear reorganization" from low-prevalence fluctuations

The manuscript frames fluctuations in specific modules (e.g., M2 peaking at 10.94% in G4; M9 dropping to 0% in G4 and recovering to 3.41% in G5) as evidence of "pronounced nonlinear developmental reorganization" and a "critical window" around 5.5 years. Given the small group sizes (n=9–10), a module dropping from ~1 subject to 0 subjects, or rising to ~1 subject, is indistinguishable from sampling noise. A single participant's data should not drive a developmental theory. The authors must:

• Report exact participant counts (integers) alongside percentages;

• Perform permutation tests or bootstrap confidence intervals for prevalence differences across groups;

• Temper the "critical window" language unless supported by more robust statistics than a single-group fluctuation.

5. Statistical significance versus practical significance

The authors report that the total number of synergies (N) was significantly higher in G5 than G1 (adjusted p = 0.035). However, the mean N increased from approximately 7 to 8 over a 24-month span. The manuscript does not report the effect size (e.g., Cohen's d or rank-biserial correlation) for this pairwise comparison. A difference of ~1 synergy, while statistically significant, may lack developmental or clinical meaningfulness. Additionally, the authors continue to rely on Kruskal-Wallis tests without covariate adjustment. While they now transparently acknowledge this as a limitation, the inability to disentangle biomechanical constraints from neurodevelopmental effects remains a major interpretive weakness that cannot be fully resolved by disclaimer alone.

Minor Issues

6. Response to reproducibility concerns (Reviewer 2) The authors reframed the low intra-individual spatial consistency (CS = 0.58) and very low temporal waveform similarity (r = 0.16) as "authentic reflection of immature neuromuscular control." While this developmental perspective is plausible, the manuscript still describes these modules as maintaining "relatively stable spatial structures" and "robust spatial consistency." There is an unresolved tension between the quantitative values (which fall below accepted adult thresholds for reproducibility) and the qualitative descriptors. I suggest the authors either report normative pediatric thresholds or explicitly state that 0.58 represents relative (not absolute) stability in this population.

7. Citation balance

The response to multiple unrelated comments (signal processing, algorithmic standardization, distal motor control, developmental neuromechanics) all cite the same author (Dhahbi W, 2025–2026). While these may be valid references, such concentrated citation of a single research group across disparate topics raises questions about citation balance and whether these specific papers represent the most established sources for each individual point.

8. Residual formatting errors

The submitted files contain residual text from the previous revision (e.g., crossed-out old methods, duplicate table captions, garbled LaTeX remnants, and Track Changes debris in the Response to Reviewers and manuscript body). The final accepted version will require thorough cleaning.

Recommendation Major Revision

The authors have made genuine methodological improvements that strengthen the analysis. However, the ethical timeline discrepancy remains unresolved and is, in my view, the most critical issue. Furthermore, the new 12-module framework introduces internal taxonomic inconsistencies and low-prevalence modules that are not yet sufficiently validated. I recommend Major Revision contingent on:

1. Ethics compliance: Provision of documented, prospective ethical approval predating June 15, 2024, or a corrected, internally consistent timeline with institutional confirmation.

2. Taxonomic transparency: Explicit reporting of the CV-of-prevalence values for all 12 modules to justify the shared/specific split; clarification of why M4 and M8 (or M6 and M8) with similar coverage are classified differently.

3. Prevalence clarification: A clear operational definition of "prevalence" with integer participant counts per group for every module.

4. Robustness checks: Bootstrap or split-half validation for low-prevalence modules (M2, M9, M12).

If the authors cannot provide satisfactory documentation for Issue #1, I recommend that the Editor consider Rejection on grounds of non-compliance with human subjects research ethics.

Reviewer #6: Manuscript ID: PONE-D-26-23547R1

Manuscript Title: Muscle Synergy Characteristics during Hands-and-Knees Crawling in Children Aged 4–6 Years

A. General Assessment

1. This revised manuscript investigates neuromotor control strategies during hands-and-knees crawling in preschool children (aged 4–6 years) through surface electromyography (sEMG) of 16 muscles and kinematic modelling. The authors extracted muscle synergies using concatenated non-negative matrix factorisation (cNMF) to examine spatiotemporal stability and age-related developmental changes.

2. The authors have performed a substantial and rigorous revision of the manuscript in response to the initial round of peer review:

2.1 Sample Retention & Power:

Data reprocessing and raising the extraction standard to VAF ≥ 90% successfully raised data retention from previous dropouts to 98% (n = 49 out of 50), resolving previous statistical power deficits in older age subgroups (e.g., G5 retention reached 100%).

2.2 Methodological Standardisation:

The arbitrary muscle dominance threshold (W > 0.5) was corrected to an evidence-based standard (W > 0.3), supported by relevant crawling literature (Xiong et al., 2018). Furthermore, differential filtering was replaced with a standardised 10- 400 Hz band-pass filter across all channels.

2.3 Clustering Improvements:

Rather than forcing a 6-synergy clustering scheme, the authors incorporated the Gap Statistic, objectively identifying 12 group-level synergy modules (M1-M12).

2.4 Appropriate Biomechanical Framing:

Claims assigning direct kinetic roles (e.g., "anti-gravity support", "forward propulsion") have been appropriately reframed as inferences or hypotheses, acknowledging the absence of ground reaction force (GRF) data.

The paper is well-written, logically structured, and provides solid insights into motor redundancy resolution strategies—specifically, the "proximal freezing" and "distal freeing" framework—in developing children.

B. Key Findings & Contributions

1. Synergy Quantity (N):

Preschoolers recruited 7.82 ± 1.05 muscle synergies on average under VAF ≥ 90%$ showing a statistically significant age-related increase (p < 0.05; G5 vs. G1).

2. Two-Tier Neural Control:

2.1 Core Shared Synergies (M4-M7, M11):

Proximal/trunk-dominated modules exhibited high prevalence (73.47%-87.76%), stable spatial structures (mean CS = 0.68-0.86), and no significant age main effects across 4–6 years.

2.2 Specific/Regulatory Synergies (M1-M3, M8-M10, M12):

Distal/fine-tuning modules demonstrated higher inter-individual temporal phase variability (Circular SD up to 44.32%) and nonlinear fluctuations in population recruitment across age groups, especially around 5.5 years (G4).

C. Minor Points & Recommendations for the Authors

While the revision is remarkably complete, a few minor textual and formatting points should be verified prior to formal publication:

1. Terminology Alignment in Text vs. Tables:

1.1 In Table 2 and the text, ensure that muscle abbreviations are entirely consistent throughout (e.g., R_TB vs. R-TRI, L_GAS vs. L-GAS). The authors updated several in-text mentions, but a quick consistency audit across Table 2 and Fig 3 labels will ensure seamless readability.

2. Degrees of Freedom Formatting:

2.1 Ensure standard formatting for terms like "degrees of freedom" (DOF) without typographical duplication in parentheses (e.g., "degrees of freedom (DOF)").

3. Figure High-Resolution Check:

3.1 Confirm that all submitted figure files (Fig1.tif through Fig5.tif) render with crisp text overlay and clean axis labels in the final typesetting build.

D. Conclusion

The authors have thoroughly addressed the reviewers' major concerns regarding statistical power, signal processing, VAF thresholds, and over-interpreted kinetic functions. The manuscript makes a valuable contribution to developmental biomechanics and motor control theory.

Reviewer #7: I sincerely thank the authors for their thoughtful responses and the thorough revisions made to the manuscript. These changes have significantly enhanced the clarity and overall quality of the work.

**********

what does this mean?). If published, this will include your full peer review and any attached files.

If you choose “no”, your identity will remain anonymous but your review may still be made public.

**Do you want your identity to be public for this peer review?**  For information about this choice, including consent withdrawal, please see our Privacy Policy

Reviewer #1: **Yes:**  Wissem Dhahbi

Reviewer #3: No

Reviewer #4: No

Reviewer #5: No

Reviewer #6: **Yes:**  Dr Rimah Melati Ab Ghani

Reviewer #7: No

**********

[NOTE: If reviewer comments were submitted as an attachment file, they will be attached to this email and accessible via the submission site. Please log into your account, locate the manuscript record, and check for the action link "View Attachments". If this link does not appear, there are no attachment files.]

To ensure your figures meet our technical requirements, please review our figure guidelines: https://journals.plos.org/plosone/s/figures

You may also use PLOS’s free figure tool, NAAS, to help you prepare publication quality figures: https://journals.plos.org/plosone/s/figures#loc-tools-for-figure-preparation.

NAAS will assess whether your figures meet our technical requirements by comparing each figure against our figure specifications.

# Author comment: Author response to Decision Letter 2

**DOI:** 10.1371/journal.pone.0358587.r004

31 Aug 2026

Part 1: Response to the Academic Editor

Editor Comment:

Congratulations on your work. Please revise the manuscript in light of the reviewers' major suggestions. Thank you.

Response：

We thank the academic editor for the positive evaluation of our research. We have comprehensively revised the whole manuscript focusing on all major methodological, statistical and interpretative concerns raised by two reviewers, and all revisions are recorded in the tracked-change manuscript file.

Revision Location：Entire manuscript with full revisions

-----------------------------------------------------------------------------------------------------------------

Part 2: Response to Reviewer 1

Comment 1:

A major weakness persists regarding the interpretation of the dimensionality reduction. The updated methodology resulted in the extraction of twelve group-level synergies from sixteen recorded muscles. Extracting twelve modules from sixteen channels represents a highly limited dimensionality reduction. The physiological validity of identifying almost as many synergies as recorded muscles requires cautious interpretation, as this outcome might reflect mathematical fractionation of the electromyographic data rather than authentic neural modularity.

Page 9, Line 306. The extraction of an average of 7.82 synergies per individual and 12 at the group level from a 16-muscle configuration is unusually high. Address whether the non-negative matrix factorization algorithm is fragmenting single muscle activations into independent modules, and discuss this specific risk within the context of the updated 90% threshold.

Response：

We sincerely thank the reviewer for this important comment. We agree that extracting an average of 7.82 synergies per individual (and 12 at the group level) from 16 recorded muscles requires careful interpretation, as the risk of mathematical over-decomposition cannot be completely excluded.

First, for the individual-level synergies, we added a discussion noting that the 90% VAF criterion is relatively strict and may require extra components to account for residual EMG variance. Furthermore, using concatenated NMF across ten continuous crawling cycles (without cycle averaging) preserves cycle-to-cycle variability. This method naturally requires more components to reconstruct the signal, which contributed to the higher individual synergy count.

Second, for the 12 group-level synergies, we clarified in the manuscript that these do not mean each child uses 12 modules at the same time. Instead, they were derived by clustering spatial weights across all 49 participants, representing a common set of recurring muscle-activation patterns shared across the group. Within this set, several highly common modules clearly contain multiple related muscles. Therefore, when a single muscle dominates a specific module, we now carefully interpret it as a local activation pattern for distal fine-tuning, rather than direct evidence of an independent neural command.

Finally, following your recommendation, we revised our interpretation of the age-related increase in synergy numbers in the Discussion. We removed statements about "adding functional modules" or "enhancing computational capacity." Instead, we now interpret this increase simply as reflecting greater differentiation and diversity of muscle-activation patterns during motor development.

Revision Location：

Discussion:

Revised the interpretation of the age-related increase in synergy numbers, removing absolute terms and focusing on the greater differentiation and diversity of muscle-activation patterns.

Added a dedicated paragraph discussing the risk of mathematical over-decomposition under the relatively strict 90% VAF threshold, and explained how preserving cycle-to-cycle variability via concatenated NMF contributes to the higher individual synergy count.

Clarified the nature of the 12 group-level synergies as a population-level repertoire, and added cautious interpretation regarding single-muscle-dominant modules as local activation patterns for distal fine-tuning.

Comment 2:

A minor weakness relates to the interpretation of motor variability. The authors correctly added a limitation regarding the inability to control for covariates such as crawling speed and anthropometrics using non-parametric statistical methods. The discussion attributes the high temporal phase dispersion to active sensorimotor exploration. The authors must acknowledge in the discussion that a portion of this temporal variability might directly stem from the uncontrolled variations in self-paced crawling speed across the distinct age groups. Page 12, Line 446. The limitations section identifies the lack of covariate control using traditional parametric tests. Add a brief note explaining how variations in self-paced crawling speed might directly inflate the reported temporal phase dispersion values observed in the specific synergies.

Response：

We sincerely thank the reviewer for this important clarification. We agree that the observed temporal phase dispersion should not be attributed solely to active motor exploration. Accordingly, we have revised the Discussion to acknowledge that part of the observed temporal phase dispersion may also arise from uncontrolled variations in self-paced crawling speed across children and age groups. We further note that differences in crawling speed may alter the relative timing of muscle activation within the crawling cycle and thereby contribute to the observed phase dispersion. The interpretation of active motor exploration has therefore been moderated to avoid attributing the observed temporal variability exclusively to central motor-control processes.

In addition, we have added a corresponding statement to the Limitations section, explicitly noting that variations in self-paced crawling speed may have contributed to the higher temporal phase dispersion observed in some specific synergies. A minor adjustment was also made to the Conclusion to ensure consistency with this more cautious interpretation.

Revision Location：

Discussion: The paragraph discussing the high temporal phase variability of the distal fine-tuning synergies (M1, M2, and M10) was revised to acknowledge that part of the observed temporal phase dispersion may be related to uncontrolled variations in self-paced crawling speed across children and age groups. The interpretation of active motor exploration was correspondingly moderated.

Limitations: The paragraph addressing uncontrolled covariates, including crawling speed, stride length, and anthropometric characteristics, was supplemented with a specific statement explaining that variations in self-paced crawling speed may contribute to the higher temporal phase dispersion observed for some synergies.

Conclusion: The corresponding statement regarding motor variability and motor exploration was slightly moderated to maintain consistency with the revised Discussion and Limitations.

Comment 3 :

Page 2, Line 41. The abstract states a mean N = 7.82 ± 1.05 synergies recruited. State the total number of muscles recorded directly in this sentence to provide immediate context for the dimensionality reduction ratio.

Response：

We sincerely thank the reviewer for this helpful suggestion. We agree that explicitly stating the number of recorded muscles alongside the mean synergy number provides clearer context for the degree of dimensionality reduction. We have therefore revised the corresponding sentence in the Abstract to explicitly state that 7.82 ± 1.05 synergies were extracted from the 16 recorded muscles.

Revision Location：

Abstract: The sentence reporting the mean number of recruited synergies was revised to:

“An average of 7.82 ± 1.05 muscle synergies were extracted from the 16 recorded muscles, with synergy number increasing significantly with age (p < 0.05).”

Comment 4:

Page 4, Line 106. The phrase "degrees of freedom redundancy (DOF redundancy)" contains unnecessary repetition. Revise this instance to "degree of freedom (DOF) redundancy" to improve readability.

Response：

We sincerely thank the reviewers for pointing out this formatting issue. We agree that the original expression “degrees of freedom redundancy (DOF redundancy)” was unnecessarily repetitive. We have revised it to “degree of freedom (DOF) redundancy” and ensured consistent terminology throughout the manuscript. The same correction was also applied to the corresponding occurrence in the Discussion to maintain consistent formatting.

Revision Location：•

Introduction: “degrees of freedom redundancy (DOF redundancy)” was revised to “degree of freedom (DOF) redundancy.”

Discussion: The corresponding occurrence of “degrees of freedom redundancy (DOF redundancy)” was also revised to “degree of freedom (DOF) redundancy.”

Comment5:

Page 7, Line 235. The reported mean intra-individual spatial consistency is 0.58, and the temporal waveform similarity is 0.16. Clarify briefly in the results text that these values are low relative to adult steady-state locomotion, directing the reader to the developmental interpretation provided in the subsequent discussion section.

Response：

We sincerely thank the reviewer for this helpful suggestion. We agree that providing a brief adult locomotion reference would help readers better contextualize the relatively low intra-individual spatial and temporal similarity observed in the preschool cohort. We have therefore added a brief comparison in the Results section, noting that these values are relatively low compared with the high intra-individual synergy consistency reported during adult steady-state locomotion. We also direct readers to the subsequent Discussion for the developmental interpretation of this variability.

Revision Location：•

Results: A brief comparative statement was added to contextualize the values:Compared with the high intra-individual consistency reported during natural-paced adult walking, these relatively low spatial and temporal similarity values indicate greater cycle-to-cycle variability in the present preschool cohort.” This addition directs readers to the subsequent Discussion for a comprehensive developmental interpretation of these findings.

-----------------------------------------------------------------------------------------------------------------

Part 3: Response to Reviewer 3

Comment 1:

Statistical Modeling and Covariates Observation: The authors indicated in their response that they were unable to apply Analysis of Covariance (ANCOVA) or Mixed-Effects Models due to the non-fulfillment of normality and homogeneity of variance assumptions for some variables, relying strictly on the Kruskal-Wallis test. Evaluation: While the authors' statistical rationale is understandable, the failure to control for variables such as "crawling speed" and "anthropometric characteristics" leaves a gap regarding whether the observed differences are purely age-related or stem from variations in individual motor performance. Required Modification: It would be beneficial to attempt data transformation (such as log transformation) to test parametric models or utilize Generalized Linear Mixed Models (GLMMs) that handle non-normal data better, while sufficiently clarifying this limitation in the limitations section.

Response：

We sincerely thank the reviewer for this constructive comment. We agree that individual differences in body size and task-related motor performance, particularly self-selected crawling speed, may influence the observed age-group differences.

We carefully considered the reviewer’s suggestions regarding data transformation and generalized linear mixed-effects modeling. However, the issue in the present study is not simply the non-normality of the data. First, our primary outcome measures have different distributional and measurement characteristics, including synergy number, N, similarity measures (CS and r), and temporal phase dispersion (Circular SD). Therefore, a single simple transformation, such as log transformation, would not provide a consistent and appropriate parametric framework for all outcome measures.

More importantly, the key task-related covariates highlighted by the reviewer, particularly crawling speed and stride characteristics, were not systematically quantified during data collection because participants crawled at a self-selected speed. Although body height and body mass were measured, they were not incorporated as covariates in the original analyses. Consequently, a post hoc transformed model or GLMM based only on the available variables would not adequately address the reviewer’s central concern regarding the potential confounding effect of individual motor performance, particularly crawling speed.

We therefore retained the original non-parametric statistical framework and have explicitly acknowledged this limitation in the revised manuscript. We have also moderated the interpretation of the observed age-group differences and clarified that the contribution of task-related motor performance and body size cannot be completely excluded.

Revision Location：

Materials and Methods – Data Acquisition: We clarified that the anthropometric measurements consisted of body height and body mass.

Discussion – Limitations: We explicitly acknowledged that body height and body mass were not included as covariates and that crawling speed, stride length, and other detailed kinematic variables were not systematically quantified. We further clarified that the observed age-group differences should not be interpreted as effects completely independent of individual body size or motor performance.

Comment 2:

Spatiotemporal Consistency Interpretation Observation: The average internal spatial consistency for individuals reached (CS = 0.58), which is relatively lower than the traditional consistency standards of (0.70 - 0.80) in mature automated movements. Evaluation: The authors successfully reframed this aspect in the discussion, considering this variability as a reflection of the "motor exploration" process in children rather than a measurement error. This interpretation must be emphasized with utmost care to prevent the muscle synergy from appearing so unstable that the study loses its reliability.

Response：

We sincerely thank the reviewer for this important clarification. We agree that the relatively lower spatial consistency (CS = 0.58) should not be interpreted as indicating unreliable or unstable muscle synergies. We have therefore clarified in the Discussion that the observed CS reflects greater cycle-to-cycle variability in the expression of the identified synergy patterns, rather than a lack of stability in the underlying muscle coordination. We have also maintained a cautious interpretation of this variability as potentially related to ongoing motor exploration.

Revision Location：

Discussion section: A clarification was added in the paragraph discussing the intra-individual spatial and temporal consistency results (the paragraph starting with "Furthermore, the data revealed...") to explicitly distinguish cycle-to-cycle variability from the reliability or stability of the underlying muscle coordination.

Comment 3:

Lack of Kinetic Validation Observation: The reviewers praised the authors' responsiveness regarding the "absence of ground reaction force (GRF) data" and the modification of terms to "hypothesized functions". Required Modification: It must be ensured that the tables (such as Table 2) and the text are completely free of any categorical phrasing indicating actual force generation, strictly adhering to probabilistic phrases such as ("it is hypothesized to contribute to...") as promised by the authors in their response.

Response：

We sincerely thank the reviewer for emphasizing this important point. We agree that, in the absence of direct GRF measurements, the biomechanical functions inferred from the identified muscle synergies should not be interpreted as confirmed mechanical functions. We would also like to clarify that the original Table 2 already framed the listed functions as hypothesized interpretations. To make this distinction even clearer and avoid any possible ambiguity, we have revised the table heading to “Hypothesized biomechanical functions.” In addition, we carefully re-checked the corresponding descriptions throughout the Results and Discussion and revised categorical wording that could imply confirmed force generation or mechanical contributions. These descriptions now consistently use more cautious expressions such as “hypothesized to contribute to,” “may contribute to,” and “potentially contributing to.” Thus, the functional interpretations are presented as hypothesis-based inferences rather than direct kinetic evidence.

Revision Location：

Table 2, Results, and Discussion：The heading of Table 2 was revised to “Hypothesized biomechanical functions” to make the hypothesis-based nature of the functional interpretation explicit. In addition, categorical functional statements in the Results and Discussion were revised to use more cautious, probabilistic wording where necessary.

-----------------------------------------------------------------------------------------------------------------

Part 3: Response to Reviewer 5

Comment 1:

Unresolved critical ethical timeline discrepancy (REQUIRES IMMEDIATE CLARIFICATION) In the revised Methods, the authors state that participants were recruited between November 2024 and May 2025, yet data collection is still reported as occurring between June 15 and November 20, 2024. The ethics approval number (SXULL2024082) implies review in August 2024. This creates an impossible chronology: data collection began in June 2024, approximately five months before the stated recruitment period and two months before the implied ethics approval date.

Response：

We sincerely thank the reviewer for identifying this apparent discrepancy in the ethical timeline. We carefully re-checked the original ethics documentation and study records and confirmed that the recruitment period reported in the revised manuscript as November 2024 to May 2025 was a typographical error. The actual participant recruitment and data collection period was June 15 to November 20, 2024, consistent with the study period specified in the ethics documentation.

We would also like to clarify that SXULL2024082 is the ethics approval/project identification number and does not represent a date. The original ethics document records the applicant’s signature on May 18, 2024, and the ethics committee approval/signature and official stamp on May 30, 2024, both of which preceded the study start date of June 15, 2024. Therefore, ethical approval was obtained before participant recruitment and data collection began.

The period from November 2024 to May 2025 referred to the subsequent data processing and analysis stage, rather than participant recruitment. To avoid further ambiguity, we have removed this analysis period from the description of participant recruitment and data collection and corrected the recruitment period in the revised manuscript to June 15 to November 20, 2024. The ethics documentation containing these dates was already submitted with the original manuscript as a supplementary document.

If the reviewer considers it helpful for clarifying the study timeline, we would be grateful for guidance as to whether the period of subsequent data processing and analysis should also be explicitly reported in the Methods section. We would be pleased to add this information if considered necessary. We sincerely apologize for the confusion caused by the typographical error.

Revision Location：

Materials and Methods – Subjects. The incorrect recruitment period “November 2024 to May 2025” has been corrected to “June 15 to November 20, 2024.” The relevant timeline statements have also been checked for consistency with the ethics documentation.

Comment 2:

Internal inconsistency in the 12-module taxonomy The new two-tier classification (shared vs. specific) applies dual criteria: prevalence >70% and coefficient of variation (CV) of prevalence across age groups <0.3. However, the classification appears inconsistently applied: • M4 (77.55% prevalence, Circular SD = 35.10%) is labeled "shared," whereas M8 (73.47% prevalence, Circular SD = 21.36%) is labeled "specific." M4 exhibits higher temporal variability and lower spatial consistency than M8, yet is classified as a stable core module. If the CV criterion explains this, the actual CV values for every module must be explicitly reported. • M6 (73.47% prevalence) is "shared," but its anatomical counterpart M8 (73.47% prevalence) is "specific." The manuscript does not justify why two modules with identical population coverage fall into different categories.

Response：

We sincerely thank the reviewer for this meticulous observation. The reviewer is absolutely correct that the Coefficient of Variation (CV) of prevalence across age groups is the precise discriminator here. We apologize for the oversight of not explicitly reporting the actual CV values for each module in the original manuscript, which understandably led to this confusion.

To clarify our rationale: our two-tier classification strictly relied on two population-level criteria prior to assessing any spatiotemporal characteristics: (1) overall prevalence > 70%, AND (2) robust developmental stability across age groups, defined as CV < 0.3.

Regarding the classification of M6 versus M8, although both modules shared an identical overall prevalence of 73.47%, their developmental trajectories differed significantly. M6 maintained a stable recruitment rate across the five age groups (CV = 0.18), fully satisfying the stability criterion (CV < 0.3), and was thus classified as a "shared" synergy. In contrast, the prevalence of M8 fluctuated substantially across age groups (CV = 0.33). Because M8 failed to satisfy the developmental stability criterion (CV ≥ 0.3), it was strictly classified as a "specific" synergy.

Furthermore, regarding M4 versus M8, the reviewer perceptively noted that M4 exhibits higher temporal variability (Circular SD) than M8. We would like to clarify that spatiotemporal characteristics (Circular SD and Spatial Consistency) were the outcomes evaluated after the classification was established; they were not the a priori criteria used to classify the modules. M4 was classified as a shared synergy because it met the dual population-level criteria: high overall prevalence (77.55%) and stable age-group distribution (CV = 0.25, which is < 0.3).

We fully agree that providing the actual CV values is crucial for methodological transparency. Per the reviewer's excellent suggestion, we have now added a "CV of Prevalence" column to Table 2, reporting the actual values for all 12 modules. Furthermore, we have expanded the Results section to explicitly justify the classifications of the modules by integrating their respective CV values into the text.

Revision Location：

Results – Table 2: A new column titled "CV of Prevalence" has been added to report the exact CV values for all 12 modules.

Results – Inter-individual clustering and spatiotemporal characteristics analysis of muscle synergies: We have updated the text to explicitly report the CV values alongside the prevalence data, thereby clarifying that M4 and M6 met the criteria for shared synergies (CV < 0.3), whereas M8 and other specific synergies did not (CV ≥ 0.3 or overall prevalence < 70%).

Comment 3:

Furthermore, several "specific" modules show extremely low population coverage: M2 (30.61%), M9 (36.73%), and M12 (26.53%). With only 49 participants, a module detected in ~8–15 individuals risks being an algorithmic artifact of over-segmentation rather than a genuine physiological synergy. The authors should provide robustness validation (e.g., split-half reliability, bootstrap resampling) to prove these low-prevalence modules are reproducible. Response：

We deeply appreciate this critical and constructive feedback. We completely agree that modules with low population coverage require rigorous robustness validation to rule out the possibility of algorithmic over-segmentation.

To address this concern, we performed a repeated split-half validation across our cohort. Specifically, the participant pool was randomly split into independent halves across multiple iterations, and muscle synergy extraction and cluster matching were re-evaluated. The spatial consistency (Cosine Similarity, CS) between the split-half subsets and the full-cohort reference templates was quantified to rigorously assess reproducibility.

The results of this validation have now been integrated into the revised manuscript (both in the Results section and Table 2), revealing a clear and informative dichotomy among the low-prevalence modules highlighted by the reviewer:

Robustness of M2 and M9:

Module 9 (M9 — Prevalence: 36.73%): Despite its lower population coverage, M9 demonstrated outstanding spatial stability under split-half validation, yielding a high mean cosine similarity of 0.95±0.09 (Median: 0.966; 95% CI: [0.806, 0.986]). This confirms that when M9 is present, its spatial structure is highly reproducible, proving it to be a genuine, albeit selectively recruited, neuromuscular control pattern rather than an algorithmic artifact.

Module 2 (M2 — Prevalence: 30.61%): Module 2 yielded a moderate split-half reliability with a mean cosine similarity of 0.77±0.22 (Median: 0.895; 95% CI: [0.358, 0.970]). While showing higher variance due to inter-individual differences in distal adjustments among the children who express it, its spatial template remains identifiable and supports the active "distal freeing" strategy characteristic of this developmental stage.

Caution regarding M12:

Module 12 (M12 — Prevalence: 26.53%): Module 12 exhibited a lower mean split-half cosine similarity of 0.45±0.29 (Median: 0.288; 95% CI: [0.154, 0.958]). Combined with its low population coverage, this indicates that M12 is sensitive to sample partitioning and carries a higher risk of being an unstable or sample-dependent peripheral component. We have explicitly acknowledged this limitation in the revised text and emphasized that M12 should be interpreted with caution.

Revision Location：•

Results Section: Added a paragraph detailing the methodology and results of the repeated split-half validation for all 12 modules within the "Inter-individual clustering and spatiotemporal characteristics analysis of muscle synergies" subsection.

Table 2: Added a new column for Split-half CS (Mean ± SD) to provide quantitative robustness evidence for each synergy module.

Discussion Section: Expanded the text near the analysis of specific synergies to note that M12 (dominated by the right rectus femoris) exhibited notably lower split-half stability (mean CS = 0.451), indicating that it should be interpreted with caution as an unstable or sample-sensitive peripheral component.

Limitations Section: Added a cautious note regarding the low spatial reliability of minor specific modules like M12, recommending future verification through larger, longitudinal cohorts.

Comment 4:

The "prevalence" metric is undefined and mathematically inconsistent In the Results, the authors report developmental trajectories using prevalence values such as: • M2 in G1: 1.30% • M2 in G4: 10.94% • M9 in G4: 0% • M9 in G5: 3.41% If "prevalence" means the percentage of participants in a group exhibiting the module, these decimals are impossible. With n=10 in G1, the smallest non-zero prevalence is 10% (1/10). With n=9 in G4, the smallest non-zero prevalence is 11.11% (1/9). Values like 1.30% or 10.94% cannot represent integer participant counts. The authors must clarify the exact formula used to calculate these percentages. If they represent something other than binary occurrence (e.g., mean W weight, VAF contribution), the terminology must be changed, as "prevalence" and "detection rate" imply a categorical present/absent variable.

Response：

We deeply appreciate the reviewer’s exceptionally careful reading and mathematical scrutiny. The reviewer is absolutely correct. We sincerely apologize for this terminological inconsistency, which understandably led to confusion.

To clarify, the decimal percentages reported in that specific paragraph (e.g., 1.30%, 10.94%) did not represent the true population prevalence. Instead, they represented the Relative Module Frequency—calculated as the total number of vectors assigned to a specific module divided by the total number of muscle synergies extracted across all participants within that specific age group (e.g., in G4, M2 accounted for 7 out of the 64 total synergy modules extracted in that group, yielding 7/64 = 10.94%).

However, we fully agree with the reviewer that labeling this metric as "prevalence" was terminologically incorrect and mathematically inconsistent with the true binary "population prevalence" (Detection Rate) used elsewhere in the manuscript (e.g., the threshold of >70% prevalence and the CV calculations). The true population prevalence is exactly as the reviewer described: a categorical present/absent variable divided by the group size (e.g., n=10 or n=9).

To completely resolve this inconsistency and ensure mathematical rigor throughout the paper, we have removed the confusing "Relative Module Frequency" decimal percentages. We have updated the developmental trajectories in both the Results and Discussion sections to report exclusively the true Population Prevalence (Detection Rate).

For example, the trajectory of M2 is now accurately reported using its true population prevalence: starting at 10% in G1 (1 out of 10 participants), peaking at 66.7% in G4 (6 out of 9 participants), and declining to 40% in G5 (4 out of 10 participants).

Revision Location：

Results: We updated the paragraph describing the developmental trajectories of specific synergies (M1, M2, M9, M10), replacing the erroneous decimal frequencies with the correct categorical prevalence rates (e.g., 10%, 50%, 66.67%). We also clarified the terminology by explicitly referring to this as "population prevalence (detection rate)."

Discussion: We corrected the corresponding prevalence data points mentioned in the Discussion (e.g., changing the M2 peak in G4 from 10.94% to 66.67%).

Comment 5:

Over-interpretation of "nonlinear reorganization" from low-prevalence fluctuations The manuscript frames fluctuations in specific modules (e.g., M2 peaking at 10.94% in G4; M9 dropping to 0% in G4 and recovering to 3.41% in G5) as evidence of "pronounced nonlinear developmental reorganization" and a "critical window" around 5.5 years. Given the small group sizes (n=9–10), a module dropping from ~1 subject to 0 subjects, or rising to ~1 subject, is indistinguishable from sampling noise. A single participant's data should not drive a developmental theory. The authors must: • Report exact participant counts (integers) alongside percentages; • Perform permutation tests or bootstrap confidence intervals for prevalence differences across groups; • Temper the "critical window" language unless supported by more robust statistics than a single-group fluctuation.

Response：

We sincerely thank the reviewer for this crucial methodological critique. You are absolutely correct that fluctuations in small cohorts (n=9-10) are heavily influenced by sampling noise, and that our previous framing of a "critical window" and "nonlinear reorganization" was a significant over-interpretation of the data. We have strictly followed your recommendations to ensure our statistical reporting and conclusions are robust and accurately reflect the sample size.

Here is how we have addressed each of your specific requirements:Reporting Exact Participant Counts: We have updated Table 2 and all developmental trajectory descriptions in the Results section to explicitly report the exact participant counts (integers) alongside the population prevalence percentages.

Bootstrap Confidence Intervals: As requested, we performed non-parametric bootstrap resampling (10,000 iterations) to calculate the 95% Confidence Intervals (CIs) for all population prevalence estimates. These CIs are now formally reported in Table 2 and throughout the text. As you rightly anticipated, the resulting wide confidence intervals (e.g., M2 in G4: [33.3%, 100.0%]) confirm the substantial variance and sampling noise inherent in our subgroup sizes.

Tempering the 'Critical Window' Language: Guided by these CI results, we have completely removed the deterministic "critical window" and "pronounced nonlinear reorganization" phrasing from the Abstract, Introduction (Hypotheses), Discussion, and Conclusion. Instead of claiming a fixed developmental shift, we now accurately interpret these wide fluctuations as evidence of high inter-individual variability. We have reframed the discussion to suggest that this variability reflects an active, trial-and-error "sensorimotor exploration" strategy used by the developing CNS to manage redundant degrees of freedom.

We believe these revisions align our conclusions strictly with the statistical reality of the data, and we are highly grateful for your guidance in strengthening the scientific rigor of our manuscript.

Revision Location：

Abstract: Revised the final sentence to remove "nonlinear reorganization" and emphasize inter-individual variability.

Introduction: Updated Hypothesis 3 to expect "highly variable and fluctuating recruitment patterns" rather than "nonlinear developmental reorganization."

Results (Table 2): Added exact participant counts and Bootstrap 95% CIs to the prevalence column.

Results (Age-related differences section): Included exact counts and 95% CIs when reporting module trajectories (e.g., M1, M2, M9, M10).

Discussion: Revised the paragraphs discussing G4/G5 transitions to interpret fluctuations as active sensorimotor exploration rather than a deterministic critical window.

Conclusion: Replaced "nonlinear developmental reorganization" with "substantial fluctuations and wide inter-individual variability."

Comment 6:

Statistical significance versus practical significance The authors report that the total number of synergies (N) was significantly higher in G5 than G1 (adjusted p = 0.035). However, the mean N increased from approximately 7 to 8 over a 24-month span. The manuscript does not report the effect size (e.g., Cohen's d or rank-biserial correlation) for this pairwise comparison. A difference of ~1 synergy, while statistically significant, may lack developmental or clinical meaningfulness. Additionally, the authors continue to rely on Kruskal-Wallis tests without covariate adjustment. While they now transparently acknowledge this as a limitation, the inability to disentangle biomechanical constraints from neurodevelopmental effects remains a major interpretive weakness that cannot be fully resolved by disclaimer alone.

Response：

We thank the reviewer for raising this thoughtful question regarding statistical versus practical significance.

To address this:Effect Size Reporting for Total Synergies (N): We have now calculated and incorporated the non-parametric effect size for the pairwise comparison between the 4-year-old group (G1, mean N=7.70) and the 6-year-old group (G5, mean N=8.80). The rank-biserial correlation yielded r_{rb}=0.64, indicating a robust, medium-to-large effect size that confirms the practical magnitude of this difference beyond a mere artifact of a low p-value.

Effect Size Reporting for Module M8: Additionally, to provide complete transparency on our significant modular findings, we have added the effect size for the temporal phase dispersion difference of Module M8 between G1 and G4 (adjusted p=0.020), which yielded r_{rb}=0.96.

Developmental and Functional Meaningfulness: While an increase of ~1 synergy module over a 24-month span appears numerically modest, it holds profound significance in the framework of motor modularity and neurodevelopment. During the preschool years (ages 4 to 6), improvements in sensorimotor integration allow the CNS to transition from conservative, rigid co-activation schemas toward a more differentiated modular repertoire. This incremental expansion reflects the progressive "freeing" of redundant degrees of freedom and a qualitative maturation of multi-joint coordination strategies, which is both biomechanically and developmentally meaningful.

Revision Location：

Results (Total Synergies): Updated the sentence to include the effect size: "...revealed that the 6-year-old group (G5) exhibited a significantly greater number of muscle synergies than the 4-year-old group (G1) (adjusted p=0.035, rank-biserial correlation r_{rb}=0.64)."

Results (Module M8): Updated the sentence to include the effect size: "...exclusively between G1 and G4 (adjusted p=0.020, rank-biserial correlation r_{rb}=0.96)..."

Discussion: Added a dedicated explanatory paragraph at the end of the first paragraph in the Discussion (following the comparison with infant crawling studies): "Although the mean number of recruited muscle synergies increased modestly from approximately 7 to 8 (specifically, from 7.70±0.82 in G1 to 8.80±0.79 in G5) over the 24-month span, this statistical difference demonstrated a robust, medium-to-large effect size (r_{rb}=0.64). From a neuromotor control perspective, this incremental expansion is far from trivial. In the context of motor modularity, adding a functional module during preschool development reflects a qualitative enhancement in the central nervous system’s computational capacity. It mirrors the progressive transition from rigid, whole-body co-activation patterns toward a more differentiated and flexible modular repertoire—successfully 'freeing' redundant degrees of freedom as sensorimotor integration matures."

Comment 7:

Response to reproducibility concerns (Reviewer 2) The authors reframed the low intra-individual spatial consistency (CS = 0.58) and very low temporal waveform similarity (r = 0.16) as "authentic reflection of immature neuromuscular control." While this developmental perspective is plausible, the manuscript still describes these modules as maintaining "relatively stable spatial structures" and "robust spatial consistency." There is an unresolved tension between the quantitative values (which fall below accepted adult thresholds for reproducibility) and the qualitative descriptors. I suggest the authors either report normative pediatric thresholds or explicitly state that 0.58 represents relative (not absolute) stability in this population. Response：

We sincerely thank the reviewer for this important clarification. We agree that the previous descriptions of “relatively stable spatial structures” and “robust spatial consistency” could overstate the degree of reproducibility indicated by the observed CS value (0.58). We have therefore revised the relevant Discussion and Conclusion to explicitly clarify that CS = 0.58 represents relative rather than absolute spatial stability within the present pediatric sample. We also revised the corresponding qualitative descriptions to avoid implying high spatial reproducibility and further distinguished the relatively consistent spatial organization from the greater temporal variability reflected by the low waveform similarity (r = 0.16). These revisions ensure that the qualitative interpretation is consistent with the quantitative findings.

Revision Location：

Discussion and Conclusion.：The relevant descriptions of spatial consistency were revised to avoid overstating reproducibility. The Discussion now explicitly states that CS = 0.58 should be interpreted as relative rather than absolute spatial stability within the present pediatric sample, and the distinction between spatial consistency and temporal variability was further clarified.

Comment 8:

Citation balance The response to multiple unrelated comments (signal processing, algorithmic standardization, distal motor control, developmental neuromechanics) all cite the same author (Dhahbi W, 2025–2026). While these may be valid references, such concentrated citation of a single research group across disparate topics raises questions about citation balance and whether these specific papers represent the most established sources for each individual point.

Response：

We sincerely thank the reviewer for raising this important concern regarding citation balance. We agree that different methodological and theoretical statements should be supported by literature that is directly relevant to each specific topic rather than relying predominantly on publications from a single research group.

In response, we carefully re-examined the relevant citations and selectively supplemented or replaced several references with established and independent sources, while retaining Dhahbi et al. references where they remain directly relevant to the corresponding statements. Specifically, Ting et al. (2015) was additionally cited in the Introduction to provide an established theoretical reference for muscle synergies; Winter (2009) was used in place of the previous reference for cycle segmentation; Besomi et al. (2020) was used for the data-preprocessing procedure; and Merletti and Cerone (2020) was additionally cited to provide an established methodological reference for EMG processing and analysis.

These changes were made selectively rather than by systematically removing the Dhahbi et al. references. Our aim was to improve citation balance while ensuring that each methodological or theoretical statement is supported by the most appropriate and representative literature.

Revision Location：

Introduction, paragraph 3: Ting et al. (2015) was added as an independent theoretical reference.

Materials and Methods – Segmentation of Cycles, paragraph 1: the previous reference was replaced with Winter (2009).

Materials and Methods – Data Preprocessing, paragraph 1: the previous reference was replaced with Besomi et al. (2020).

Materials and Methods – Muscle Synergy Extraction and Non-Negative Matrix Factorization, paragraph 1: Merletti and Cerone (2020) was added as an independent methodological reference.

-----------------------------------------------------------------------------------------------------------------

Part 4: Response to Reviewer 6

Comment 1:

Terminology Alignment in Text vs. Tables: 1.1 In Table 2 and the text, ensure that muscle abbreviations are entirely consistent throughout (e.g., R_TB vs. R-TRI, L_GAS vs. L-GAS). The authors updated several in-text mentions, but a quick consistency audit across Table 2 and Fig 3 labels will ensure seamless readability.

Response：

We sincerely thank the reviewer for pointing out this terminology consistency issue. We have conducted a comprehensive consistency check of muscle abbreviations throughout the manuscript, with particular attention to Table 2 and the labels in Fig. 3. All muscle abbreviations have now been standardized to ensure consistent naming and formatting across the text, tables, and figures.

Revision Location：

Table 2, Fig. 3, and the corresponding descriptions throughout the manuscript. Muscle abbreviations were systematically checked and standardized for consistency.

Comment 2:

Degrees of Freedom Formatting: 2.1 Ensure standard formatting for terms like "degrees of freedom" (DOF) without typographical duplication in parentheses (e.g., "degrees of freedom (DOF)")

Response：

We sincerely thank the reviewers for pointing out this formatting issue. We agree that the original expression “degrees of freedom redundancy (DOF redundancy)” was unnecessarily repetitive. We have revised it to “degree of freedom (DOF) redundancy” and ensured consistent terminology throughout the manuscript. The same correction was also applied to the corresponding occurrence in the Discussion to maintain consistent formatting.

Revision Location：

Introduction: “degrees of freedom redundancy (DOF redundancy)” was revised to “degree of freedom (DOF) redundancy.”

Discussion: The corresponding occurrence of “degrees of freedom redundancy (DOF redundancy)” was also revised to “degree of freedom (DOF) redundancy.”

Comment 3:

Figure High-Resolution Check: 3.1 Confirm that all submitted figure files (Fig1.tif through Fig5.tif) render with crisp text overlay and clean axis labels in the final typesetting build.

Response：

We sincerely thank the reviewer for this helpful reminder. We have carefully checked all submitted figure files (Fig. 1–Fig. 5) and confirmed that the original TIFF files provide clear and legible text, axis labels, tick marks, legends, and other graphical elements at the submitted resolution. No apparent pixelation, distortion, or loss of readability was observed. The figure files have therefore been carefully reviewed and prepared to ensure their suitability for the final typesetting process

Revision Location：

Fig. 1–Fig. 5. All figure files were reviewed and, where necessary, re-exported as high-resolution TIFF files to ensure clear text, axis labels, legends, and graphical elements in the final submission.

Attachment Submitted filename: Response_to_Reviewers_auresp_2.docx

# Editor report: Decision Letter 2

**DOI:** 10.1371/journal.pone.0358587.r005

**Authors:** Mário Espada

2 Sep 2026

Muscle Synergy Characteristics during Hands-and-Knees Crawling in Children Aged 4–6 Years

PONE-D-26-23547R2

Dear Dr. Yi Jia,

We’re pleased to inform you that your manuscript has been judged scientifically suitable for publication and will be formally accepted for publication once it meets all outstanding technical requirements.

Within one week, you’ll receive an e-mail detailing the required amendments. When these have been addressed, you’ll receive a formal acceptance letter and your manuscript will be scheduled for publication.

An invoice will be generated when your article is formally accepted. Please note, if your institution has a publishing partnership with PLOS and your article meets the relevant criteria, all or part of your publication costs will be covered. Please make sure your user information is up-to-date by logging into Editorial Manager at Editorial Manager® and clicking the ‘Update My Information' link at the top of the page. For questions related to billing, please contact billing support.

If your institution or institutions have a press office, please notify them about your upcoming paper to help maximize its impact. If they’ll be preparing press materials, please inform our press team as soon as possible -- no later than 48 hours after receiving the formal acceptance. Your manuscript will remain under strict press embargo until 2 pm Eastern Time on the date of publication. For more information, please contact onepress@plos.org.

Kind regards,

Mário Espada, PhD

Academic Editor

PLOS One

# Editor report: Acceptance letter

**DOI:** 10.1371/journal.pone.0358587.r006

**Authors:** Mário Espada

PONE-D-26-23547R2

PLOS One

Dear Dr. Jia,

I'm pleased to inform you that your manuscript has been deemed suitable for publication in PLOS One. Congratulations! Your manuscript is now being handed over to our production team.

At this stage, our production department will prepare your paper for publication. This includes ensuring the following:

* All references, tables, and figures are properly cited

* All relevant supporting information is included in the manuscript submission,

* There are no issues that prevent the paper from being properly typeset

You will receive further instructions from the production team, including instructions on how to review your proof when it is ready. Please keep in mind that we are working through a large volume of accepted articles, so please give us a few days to review your paper and let you know the next and final steps.

Lastly, if your institution or institutions have a press office, please let them know about your upcoming paper now to help maximize its impact. If they'll be preparing press materials, please inform our press team within the next 48 hours. Your manuscript will remain under strict press embargo until 2 pm Eastern Time on the date of publication. For more information, please contact onepress@plos.org.

You will receive an invoice from PLOS for your publication fee after your manuscript has reached the completed accept phase. If you receive an email requesting payment before acceptance or for any other service, this may be a phishing scheme. Learn how to identify phishing emails and protect your accounts at https://explore.plos.org/phishing.

If we can help with anything else, please email us at customercare@plos.org.

Thank you for submitting your work to PLOS One and supporting open access.

Kind regards,

PLOS One Editorial Office Staff

on behalf of

Dr. Mário Espada

Academic Editor

PLOS One
